# Self-supervised multimodal learning for group inferences from MRI data: Discovering disorder-relevant brain regions and multimodal links

**DOI:** 10.1016/j.neuroimage.2023.120485

**Published:** 2023-12-16

**Authors:** Alex Fedorov, Eloy Geenjaar, Lei Wu, Tristan Sylvain, Thomas P. DeRamus, Margaux Luck, Maria Misiura, Girish Mittapalle, R. Devon Hjelm, Sergey M. Plis, Vince D. Calhoun

**Affiliations:** a Tri-Institutional Center for Translational Research in Neuroimaging and Data Science (TReNDS), Georgia State, Georgia Tech, Emory, Atlanta, GA, USA; b Mila - Quebec AI Institute, Montréal, QC, Canada; c Apple Machine Learning Research, Seattle, WA, USA; d Borealis AI, Montréal, QC, Canada

**Keywords:** Deep learning, Multimodal data, Mutual information, Self-supervised learning, Alzheimer’s disease

## Abstract

In recent years, deep learning approaches have gained significant attention in predicting brain disorders using neuroimaging data. However, conventional methods often rely on single-modality data and supervised models, which provide only a limited perspective of the intricacies of the highly complex brain. Moreover, the scarcity of accurate diagnostic labels in clinical settings hinders the applicability of the supervised models. To address these limitations, we propose a novel self-supervised framework for extracting multiple representations from multimodal neuroimaging data to enhance group inferences and enable analysis without resorting to labeled data during pre-training. Our approach leverages Deep InfoMax (DIM), a self-supervised methodology renowned for its efficacy in learning representations by estimating mutual information without the need for explicit labels. While DIM has shown promise in predicting brain disorders from single-modality MRI data, its potential for multimodal data remains untapped. This work extends DIM to multimodal neuroimaging data, allowing us to identify disorder-relevant brain regions and explore multimodal links. We present compelling evidence of the efficacy of our multimodal DIM analysis in uncovering disorder-relevant brain regions, including the hippocampus, caudate, insula, - and multimodal links with the thalamus, precuneus, and subthalamus hypothalamus. Our self-supervised representations demonstrate promising capabilities in predicting the presence of brain disorders across a spectrum of Alzheimer’s phenotypes. Comparative evaluations against state-of-the-art unsupervised methods based on autoencoders, canonical correlation analysis, and supervised models highlight the superiority of our proposed method in achieving improved classification performance, capturing joint information, and interpretability capabilities. The computational efficiency of the decoder-free strategy enhances its practical utility, as it saves compute resources without compromising performance. This work offers a significant step forward in addressing the challenge of understanding multimodal links in complex brain disorders, with potential applications in neuroimaging research and clinical diagnosis.

## 1. Introduction

The brain is a vastly complex organ whose function relies on simultaneously operating multitudes of distinct biological processes. As a result, individual imaging techniques often capture only a single facet of the information necessary to understand a dysfunction or perform a diagnosis. As an illustration, structural MRI (sMRI) captures static but relatively precise anatomy, while fMRI measures the dynamics of hemodynamic response but with substantial noise. Unimodal brain imaging analyses have been shown to potentially yield misleading conclusions (Calhoun and Sui, 2016; Plis et al., 2011), which is unsurprising given fundamental differences in measured information of the modalities, as each modality has its own inherent flaws.

To address the limitations of unimodal analyses, it is natural to turn to multimodal data to leverage a wealth of complementary information, which is key to enhancing our knowledge of the brain and developing robust biomarkers. Unfortunately, multimodal modeling is often challenging, as finding points of convergence between different multimodal views of the brain is a nontrivial problem.

This work proposes the adoption of self-supervised learning (SSL), specifically Deep InfoMax (DIM) (Hjelm et al., 2019), to address the challenge of multimodal modeling by estimating multimodal relationships via mutual information. Thereby allowing us to achieve the following three goals. Our primary goal in addressing multimodal modeling is to understand how to represent multimodal neuroimaging data by exploiting unique and joint information in two modalities. Secondly, we want to understand how to measure the amount of joint information in representations between modalities and how it is interplayed with discriminative performance. As the third goal, we want to understand whether unsupervised multimodal representations capture group discriminative brain regions and the links between different modalities (e.g., T1-weighted structure measurements and resting functional MRI data). The final goal is to understand how to exploit co-learning (Baltrušaitis et al., 2018), especially in cases where one modality is particularly challenging to learn. By achieving these three goals in this work, we address three of the five multimodal challenges (Baltrušaitis et al., 2018): representation, alignment, and co-learning, leaving only generative translation and fused prediction for future work.

We present a general framework for self-supervised multimodal neuroimaging. The proposed approach can capitalize on the available joint information to show competitive performance relative to supervised methods. Our approach opens the door to additional data discovery. It enables characterizing subject heterogeneity in the context of imperfect or missing diagnostic labels and, finally, can facilitate the visualization of complex relationships.

### 1.1. Related work

In neuroimaging, linear independent component analysis (ICA) (Comon, 1994) and canonical correlation analysis (CCA) (Hotelling, 1992) are commonly used for latent representation learning and intermodal link investigation. Joint ICA (jICA) (Moosmann et al., 2008) performs ICA on concatenated representation for each modality. jICA has been extended with multiset canonical correlation (mCCA+ jICA) (Sui et al., 2011) and ICA with spatial CCA (sCCA+ICA) (Sui et al., 2010) which mitigate limitations of ICA, jICA and CCA applied separately. These jICA or jICA-adjacent methods all estimate a joint representation. Another approach, parallel ICA (paraICA) (Liu et al., 2009), simultaneously learns independent components for fMRI and SNP data that maximize the correlation between specific multimodal pairs of columns in the mixing matrix of different modalities. A recent improvement over paraICA, aNy-way ICA (Duan et al., 2020), can scale to any number of modalities and requires fewer assumptions.

Most of the available multimodal imaging analysis approaches, including those mentioned above, rely on linear decompositions of the data. However, recent work suggests the presence of nonlinearities in neuroimaging data that can be exploited by deep learning (DL) (Abrol et al., 2021). Likewise, correspondence between modalities is unlikely to be linear (Calhoun and Sui, 2016). These findings motivate the need for deep nonlinear models. Supervised DL models have proven successful in neuroimaging due to their ease of use. These models show unparalleled performance, mainly in the abundance of training data: a data sample paired with a corresponding label. However, supervised models are prone to the shortcut learning phenomenon (Geirhos et al., 2020); when a model hones in on trivial patterns in the training set that are sufficient to classify the available data but are not generalizable to data unseen at training. Next, it has been shown that supervised models can memorize noisy labels (Arpit et al., 2017), which are commonplace in healthcare (Pechenizkiy et al., 2006; Rokham et al., 2020). Furthermore, supervised methods have also been shown to be data-inefficient (Hénaff et al., 2020) while labels in medical studies are costly and scarce. Finally, in many cases, diagnostic labels are based on self-reports and interviews and thus may not accurately reflect the underlying biology (Rokham et al., 2020). Many of these problems can be addressed with unsupervised learning and, more recently, self-supervised learning (SSL) (Dosovitskiy et al., 2014). In SSL, a model trains on a proxy task that does not require externally provided labels. SSL has been shown to improve robustness (Hendrycks et al., 2019), data-efficiency (Hénaff et al., 2020), and can outperform supervised approaches on image recognition tasks (Caron et al., 2020).

In the early days of the current wave of unsupervised deep learning, common approaches were based on deep belief networks (DBNs) (Srivastava and Salakhutdinov, 2012a; Plis et al., 2014), and deep Boltzmann machines (DBMs) (Srivastava and Salakhutdinov, 2012b; Hjelm et al., 2014; Suk et al., 2014). However, DBNs and DBMs are challenging to train. Later, deep canonical correlation analysis (DCCA) (Andrew et al., 2013) was introduced for multiview unsupervised learning. DCCA (Andrew et al., 2013), and its successor, deep canonically correlated autoencoder (DCCAE) (Wang et al., 2015), are trained in a two-stage procedure. A neural network trains unimodally in the first stage via layer-wise pretraining or an autoencoder. In the second stage, CCA captures joint information between modalities. Due to the need for a decoder, the autoencoding approaches demand high computational and memory requirements for full brain data as most brain segmentation models are still working on 3D patches (Fedorov et al., 2017; Henschel et al., 2020).

Among many self-supervised learning approaches, we are specifically interested in methods that use maximization of mutual information, such as Deep Infomax (DIM) (Hjelm et al., 2019) and contrastive predictive coding (CPC) (Oord et al., 2018). These methods can naturally be extended to modeling multimodal data (Fedorov et al., 2021) compared to other self-supervised pre-text tasks (Misra and Maaten, 2020) (e.g. relative position (Doersch et al., 2015), rotation (Gidaris et al., 2018), colorization (Zhang et al., 2016)). The maximization of mutual information in these methods allows a predictive relationship between representations at different levels as a learning signal for training. Specifically, the learning signal in DIM (Hjelm et al., 2019) is the relationship between the intermediate representation of a convolutional neural network (CNN) and the whole representation of the input. For example, in (CPC) (Oord et al., 2018), this is done between the context and a future intermediate state. Both DIM and CPC have been successfully extended and applied unimodally for the prediction of Alzheimer’s disease from sMRI (Fedorov et al., 2019), transfer learning with fMRI (Mahmood et al., 2019, 2020), and brain tumor, pancreas tumor segmentation, and diabetic retinopathy detection (Taleb et al., 2020). In addition, these models do not reconstruct as part of their learning objective, unlike autoencoders. The reconstruction-free model saves a lot of compute and memory, especially for volumetric medical imaging applications.

In multiview and multimodal settings, self-supervised learning has enabled state-of-the-art results on various computer vision problems via maximization of mutual information between different views of the same image (Bachman et al., 2019; Tian et al., 2019; Chen et al., 2020), and multimodal data (e.g., visual, audio, text) (Miech et al., 2020; Alayrac et al., 2020; Radford et al., 2021; Sylvain et al., 2020a). These SSL approaches capture the joint information between two corresponding distorted or augmented images, image–text, video–audio, or video–text pairs. In the multiview case (Bachman et al., 2019; Tian et al., 2019; Chen et al., 2020), the models learn transformation-invariant representations by capturing the joint information while discarding information unique to a transformation. In the case of multimodal data, the models learn modality-invariant (Saito et al., 2016) representations known as retrieval models. The same ideas have been extended to the multidomain scenario to learn domain-invariant (Feng et al., 2019b) representations. However, when one modality can capture the anatomy and the other can capture brain dynamics, the joint information alone will not be sufficient due to the unique modality-specific information content that each modality measures. We hypothesize that we additionally need to capture unique modality-specific information.

Most of the described multiview, multidomain, and multimodal work can be viewed as a coordinated representation learning (Baltrušaitis et al., 2018). In coordinated representation learning, we learn separate representations for each view, domain, or modality. The representations are coordinated through an objective function by optimizing a similarity measure with possible additional constraints (e.g., orthogonality in CCA). In this case, the objective function mainly captures joint information between the *global* latent representation of modalities that summarize the whole input. However, such a framework only considers a *global*-*global* relationship between modalities. To resolve this limitation, we can consider intermediate representations, namely as *local* representation that captures local information about the input. That would allows us to capture *local-to-local*, *global-to-global*, *local-to-global* multimodal relationships. Previously, augmented multi-scale DIM (AMDIM) (Bachman et al., 2019), cross-modal DIM (CM-DIM) (Sylvain et al., 2019, 2020b), and spatio-temporal DIM (ST-DIM) (Anand et al., 2019) used *local* intermediate representation of convolutional layers to capture multi-scale relationships between multiple views, modalities or time frames. Similarly, multi-scale relationships improve semantic correspondences between two views of the same image in EsViT (Li et al., 2021). Furthermore, from neuroscientific and neurological perspectives, the brain is a complex system with multiple scales of an organization where local changes can influence global changes (Sheng et al., 2017; Kim et al., 2013; Filippi et al., 2020; Liu et al., 2022; Xie et al., 2015), or local changes can influence local changes (Frisoni et al., 2008) between structure and function. Thus, we hypothesize that multi-scale relationships between modalities can also be used to learn representations from multimodal neuroimaging data. To verify this, we extend the coordinated representation learning framework to a multi-scale coordinated representation framework for multimodal data.

### 1.2. Contributions

First, we propose a multi-scale coordinated framework as a family of models. The family of models is inspired by many published SSL approaches based on the maximization of mutual information that we combine in a complete taxonomy. The family of methods within this taxonomy covers multiple inductive biases that can capture joint inter-modal and unique intra-model information. In addition, it covers multi-scale multimodal relationships in the input data.

Secondly, we provide a methodology to evaluate learned representation exhaustively. We thoroughly investigate the models on a multimodal dataset OASIS-3 (LaMontagne et al., 2019) by evaluating performance on two classification tasks, exploring label efficiency and transfer learning to out-of-distribution subset of the data, measuring the amount of joint information in representations between modalities, via Central Kernel Alignment (CKA) (Kornblith et al., 2019) and interpreting the representation in the brain voxel space.

Our results prove that self-supervised models yield useful predictive representations for classifying a spectrum of Alzheimer’s phenotypes. The proposed models can achieve near-supervised performance on the classification tasks, outperform previous autoencoder and CCA-based approaches, and improve label efficiency. Furthermore, the proposed multimodal models can capture higher content of joint information between modalities than CCA-based approaches.

We show that self-supervised models can uncover regions of interest supported by the literature such as the cuneus (Wu et al., 2021), subthalamus hypothalamus (Zhu et al., 2019; Ríos et al., 2022), thalamus (Coupé et al., 2019), insula (Philippi et al., 2020), hippocampus (Yang et al., 2022).

We show that multimodal self-supervised models can uncover multimodal links between anatomy and brain dynamics supported by previous literature such as subthalamus hypothalamus and posterior cingulate cortex (Laxton et al., 2010), posterior cingulate cortex and cerebellum (Zimny et al., 2011), precuneus and cerebellum (Parker et al., 2020), cerebellum and thalamus (Martí-Juan et al., 2023).

## 2. Materials and methods

In this section, we establish the foundation of our framework by presenting an overview of coordinated representation learning, extending this concept to multi-scale coordinated learning, then delving into mutual information, followed by outlining the taxonomy and established baselines.

### 2.1. Overview of coordinated representation learning

To help the reader understand the foundation of our framework, we start with the idea of coordinated representation learning (Baltrušaitis et al., 2018).

Let $x=\left(x^{1},\ldots ,x^{M}\right)$ be an arbitrary sample of M related input modalities collected from the same subject. Taken together, a set of samples comprise a multimodal dataset $D={\left\{\left(x_{i}^{1},\ldots ,x_{i}^{M}\right)\right\}}_{i=1}^{N}$. The modalities of interest are sMRI and rs-fMRI, represented as T1 and fALFF volumes, respectively. Thus, M=2 and $x^{m}\in R^{d_{1}\times d_{2}\times d_{3}}$, a $d_{1}\times d_{2}\times d_{3}$ tensor. While inter-modal relationships can be as complex as a concurrent acquisition of neuroimaging modalities, we pair sMRI and rs-fMRI based on the temporal proximity between sessions of the same subject from the OASIS-3 (LaMontagne et al., 2019) dataset.

Let $D=\left\{\left(x_{1},\ldots ,x_{M}\right)∼P\left(D_{1},\ldots ,D_{M}\right)\right\}$ be a multimodal dataset consisting of N-paired samples $\left(x_{1},\ldots ,x_{M}\right)$ from M unimodal datasets. Then for each sample $x_{m}$, we want to learn d-dimensional representation $z_{m}=E_{m}\left(x_{m}\right)\in R^{d}$ by passing a sample through encoder $E_{m}$. The encoder $E_{m}$ is a nonlinear projection parameterized by a deep neural network. Note that the encoder is individual for each modality m. To learn the parameters of each encoder $E_{m}$, we want to optimize multimodal objective $ℒ\left(P\left(D_{1},\ldots ,D_{M}\right)\right)$. By other means, we constraint the parameters of the encoders by multimodal objective $ℒ\left(z_{1},\ldots ,z_{M}\right)$ to coordinate the representations across modalities, for example, by enforcing higher similarity distance or correlation between representation from different modalities. In this setup, we learn separate latent representations for each modality.

In this work, we parameterize the encoder $E^{m}$ for each modality m with a volumetric deep convolutional neural network. To learn each encoder’s parameters, we optimize an objective function $ℒ=ℒ\left(z^{1},\ldots ,z^{M}\right)$ that incorporates the representation of each modality and coordinates them. The coordination objective encourages each encoder to learn a latent representation for their respective modality informed by the other modalities. This cross-modal influence is learned during training and captured in each encoder’s parameters. Hence, a representation of an unseen subject’s modality will capture cross-modal influences without a contingency on the availability of the other modalities. One common choice concerning cross-modal influences is to coordinate representations across modalities $z^{m}$ by maximizing their similarity metric (Frome et al., 2013) or correlation via CCA (Andrew et al., 2013) between representation vectors.

To summarize, in coordinated representation learning, modality-specific encoders learn to generate representations in a cross-coordinated manner guided by an objective function.

The main limitation of the coordinated representation framework is its exclusive focus on capturing joint information between modalities instead of capturing information exclusive to each modality. Thus, *DCCA* (Andrew et al., 2013) and *DCCAE* (Wang et al., 2015) employ coordinated learning only as a secondary stage after pretraining the encoder. The first stage in these methods focuses on learning modality-specific information via layer-wise pretraining of the encoder in *DCCA* or pretraining of the encoder with an autoencoder (*AE*) in *DCCAE*. On the other hand, deep collaborative learning (DCL) (Hu et al., 2019a) attempts to capture modality-specific information using a supervised objective for phenotypical information with respect to each modality, in addition to CCA.

Another limitation that previous work has not considered is using intermediate representation in the encoder. Intermediate representations have been integral to the success of U-Net architecture in biomedical image segmentation (Ronneberger et al., 2015), brain segmentation tasks (Henschel et al., 2020), achieving state-of-the-art results with self-supervised learning on natural image benchmarks with DIM (Hjelm et al., 2019) and AMDIM (Bachman et al., 2019), and achieving near-supervised performance in Alzheimer’s disease progression prediction, with self-supervised pretraining (Fedorov et al., 2019).

Our work addresses these limitations by proposing a multi-scale coordinated learning framework.

### 2.2. Multi-scale coordinated learning

We re-introduce intermediate representations to introduce multi-scale coordinated learning and explain how they can benefit multi-modal modeling.

Each encoder $E^{m}$ produces intermediate representations. Specifically, if the encoder $E^{m}$ is a convolutional neural network (CNN) with L layers, each subsequent layer l={1,…,L} in the CNN represents a more significant part of the original input data. Furthermore, each of these scales, which corresponds to the depth of a layer, is an increasingly nonlinear transformation of the input and produces a more abstract representation of that input relative to the previous scales. The intermediate representations of layer l are S×o convolutional features ${\left\{c_{l}^{m}\right\}}_{S_{l}}$, where S is the number of locations in the convolutional features of layer l, and o is the number of channels. These features are also often referred to as activation maps within the network. For example, if the input is a 3D cube, the arbitrary feature size s within a layer l of the CNN will be s×s×s. Thus, each intermediate representation will have $S=s^{3}$ locations. Each location in the intermediate representation has a receptive field (Araujo et al., 2019) that captures a specific subset of the input sample. Each intermediate representation thus captures some of the input’s *local* information, while the latent representation $\left(z^{m}\right)$ captures the input’s *global* information.

**Fig. 1.**
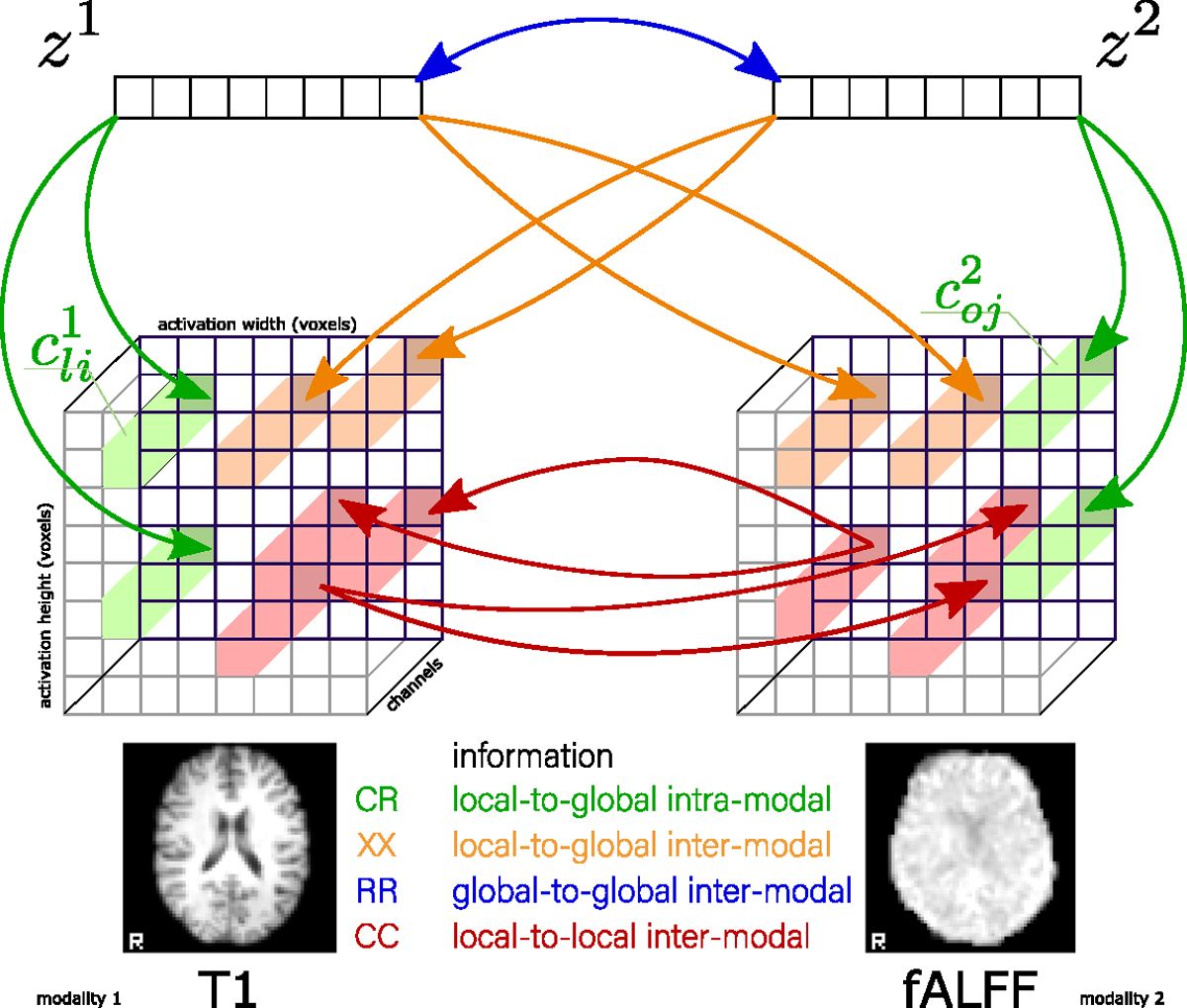
The concept behind the multi-scale coordinated learning based on four principle relationships: *Convolution-to-Representation* (CR), *Cross Convolution-to-Representation* (XX), *Representation-to-Representation* (RR), and *Convolution-to-Convolution* (CC). Each colored vector in the convolution activation map $c_{li}^{1}$ and $c_{oj}^{2}$ corresponds to arbitrary locations i and j in features maps of layers l and o for modalities 1 and 2, respectively. We use “local representation” to denote each location in the convolutional activation map: the c vector spanning the channels. The latent representation vector z is the d-dimensional *global* representation. To avoid clutter, we display only a slice of data but layer activations a volume per channel in our applications.

With two scales and two modalities, we can define multi-scale coordinated learning based on four objectives, schematically shown in Fig. 1. The *Convolution-to-Representation* (CR) objective captures modality-specific information as *local-to-global* intra-model interactions. The *Cross Convolution-to-Representation* (XX) objective captures joint inter-modal *local-to-global* interactions between the *local* representations in one modality and the *global* representation in another modality. The *Representation-to-Representation* (RR) objective captures joint information between *global* inter-modal representations as *global-to-global* interactions. The *Convolution-to-Convolution* (CC) objective captures joint information between *local* inter-modal representations as *local-to-local* interactions.

Thus, we can capture modality-specific information and multimodal relationships at multiple scales. These extensions cover two previously mentioned limitations in the coordinated learning framework. Our extensions also allow us to define a complete taxonomy of models that can be constructed based on these four principal interactions and to show how these compare to or supersede related work.

First, we will define an estimator of mutual information to construct an objective ℒ based on these multi-scale coordinated interactions. This estimator will define each of the four objectives as a mutual information maximization problem that can be used to encourage the interactions between the corresponding representations. Lastly, we explain how one can construct an objective ℒ for a multi-scale coordinated representation learning problem, based on a combination of the four primary objectives between *global* and *local* features, and, additionally, show how these compare to related work.

### 2.3. Mutual information maximization

To estimate mutual information between random variables X and Y, we use a lower bound based on the noise-contrastive estimator (InfoNCE) (Oord et al., 2018).

(1) $$I\left(X;Y\right)\geq I^{\mathrm{InfoNCE}}\left(X;Y\right)=\frac{1}{N}\sum_{i=1}^{N}\log\frac{e^{f\left(x_{i},y_{i}\right)}}{\frac{1}{N}\sum_{j=1}^{N}1_{i\neq j}e^{f\left(x_{i},y_{j}\right)}},$$

where the samples $x_{i}∼X$, $y_{i}∼Y$ construct pairs: $\left(x_{i},y_{i}\right)$ sampled from the joint P(X,Y) (*positive* pair) and ${\left(x_{i},y_{j}\right)}_{i\neq j}$ sampled from the product of the marginals P(X)⊗P(Y) (*negative* pair). The $x_{i}\in R^{d}$ and $y_{i}\in R^{d}$ represent d-dimensional representation vectors, and can be local or *global* representations. The function $f\;:R^{d}\to R$ in Eq. (1) is a scoring function that maps its input vectors to a scalar value and is supposed to reflect the goodness of fit. This functions f is also known as the *critic function* (Tschannen et al., 2020). The encoder is optimized to maximize the critic function for a positive pair and minimize it for a negative pair, such that $f\left(x_{i},y_{i}\right)\gg f{\left(x_{i},y_{j}\right)}_{i\neq j}$. Our choice of the critic function is a scaled dot-product (Bachman et al., 2019) and is defined as:

(2) $$f(x,v)=\frac{x^{⊤}y}{\sqrt{d}}$$

### 2.4. Taxonomy for multi-scale coordinated learning

**Fig. 2.**
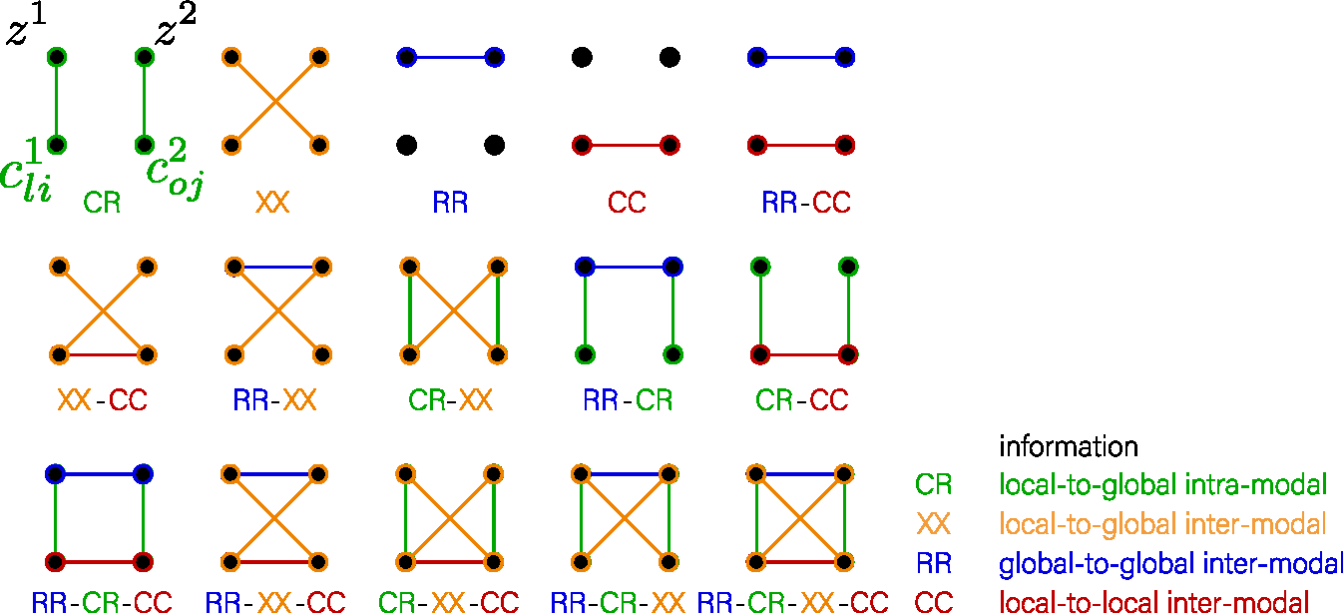
The complete taxonomy of interactions, based on the four principle interactions. The lower dots are the convolutional activations, whereas the upper dots are the global representations. The interactions are defined between 1st modality (left) and second modality (right). The combinations represent the names of the models that are based on these four interactions: *CR*, *XX*, *RR*, and *CC*. The colors follow the colormap from Fig. 1.

Given the mutual information estimator, we can construct four primary objectives and then use those to construct a complete taxonomy of interactions, shown in Fig. 2. Each option within the taxonomy specifies a unique optimization objective ℒ(𝒟). Notably, the first row of the figure shows the principal losses: *CR*, *XX*, *RR*, and *CC*, - that we defined before. The remaining parts of the taxonomy are constructed by adding a composition of the principal losses. For example, the 5th combination *RR-CC* is the sum of the two primary objectives *RR* and *CC*.

To discuss the options in the taxonomy, we first reintroduce some notations. A *local* representation $c_{ld}^{m}$ is any arbitrary location d∈{1,…,S} in the convolutional feature $c_{m}^{l}$ from a convolutional layer l, with S locations. A location is represented as the C-dimensional vector, where C is the number of channels of the convolutional activation map for layer l. The choice of the layer l is a hyperparameter and can be guided by the following intuition. The selected layer l should not be too close to the last layer, nor be the final layer itself. This is because, in such cases, the local and *global* content of the input tend to exhibit high similarity, often sharing nearly identical receptive fields. This similarity can lead to overly simplistic solutions that fail to learn meaningful representations. The concepts of *local* and *global* representations, can effectively serve as a strategy for block-wise pretraining in analogy to layer-wise training. This approach is exemplified in the Greedy Deep InfoMax (GIM) (Löwe et al., 2019). Secondly, the chosen layer l should not be too close to or be the first layer. This will lead to a *local* representation with a tiny receptive field that only captures hyper-local information of the input. As well as using the input data is primarily oriented towards capturing the intensity of pixels and voxels. This approach, however, has been proven ineffective, as highlighted in the DIM study (Hjelm et al., 2019). A significant factor contributing to this ineffectiveness is the inherent and substantial noisiness present at both the pixel and voxel levels within the input data.

The global representation is the encoder’s $E^{m}$ latent representation $z^{m}=E^{m}\left(x^{m}\right)$ that summarizes the whole input. The *global* representation is a d-dimensional vector, where d is also a hyperparameter. With this *global* representation, we also define a d-dimensional space, wherein we compute scores with the critic function f. However, the *local* representation is a C-dimensional vector. To overcome the difference in size, we add *local* projection head ℓ. This projection head takes the C-dimensional *local* representation from layer l in the encoder and projects it to the d-dimensional space to compute scores with f in this d-dimensional space. This projection is also parameterized by a neural network and separate for each modality. In addition, we introduce a *global* projection head g. Both the *local* and *global* projection heads are shown to improve the training performance in DIM (Hjelm et al., 2019) and SimCLR (Chen et al., 2020), respectively.

The first objective (CR), in the top left corner of Fig. 2, trains two independent encoders, one for each modality, with a unimodal loss function that maximizes the mutual information between local $c_{ld}^{m}$ and global $z^{m}$ representations. This objective directly implements the Deep InfoMax (DIM) (Hjelm et al., 2019). The idea behind this approach is to maximize the information between the encoder’s lowest and highest scales. In other words, the local representations are driven to predict the global representation. The objective for an arbitrary layer l is defined as:

(3)

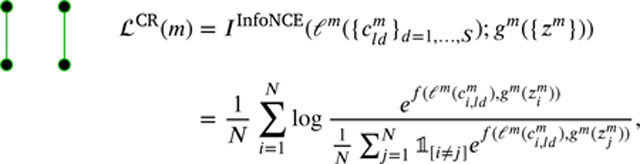


where we only define the following objective ${ℒ}^{CR}(m)$ for a modality m. The objective has to be computed for each modality.

The CR objective can be extended to the multimodal case by measuring the inter-modal mutual information between local $c_{ld}^{m}$ and global $z^{k}$ representations of modality m and k≠m, respectively. We call this multimodal objective Cross Convolution-to-Representation (XX), and it is shown second from the top left in Fig. 2. This objective has previously been used in the context of augmented multiscale DIM (AMDIM) (Bachman et al., 2019), cross-modal DIM (CM-DIM) (Sylvain et al., 2020b), and spatio-temporal DIM (ST-DIM) (Anand et al., 2019). We define it as

(4)

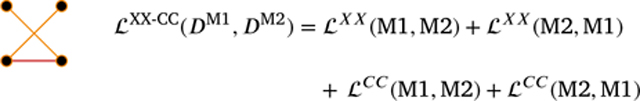


where the objective ${ℒ}^{XX}(m,k)$ is defined for a pair of modalities m and k. The objective has to be computed for all possible pairs of modalities. In the case of symmetric coordinated fusion, the symmetry has to be preserved for modalities m and k by computing both ${ℒ}^{XX}(m,k)$ and ${ℒ}^{XX}(k,m)$, whereas for asymmetric fusion, this is not the case.

The third elementary objective measures mutual information between the global representation of one modality $z^{m}$ and the global representation of another modality $z^{k}$, k≠m. This objective is called Representation-to-Representation (*RR*), shown as the third in the top row of Fig. 2. This interaction has been used in many prior contrastive multiview work (Tian et al., 2019; Chen et al., 2020; He et al., 2020; Caron et al., 2020) and *DCCA* (Andrew et al., 2013). The RR objective is defined as

(5)

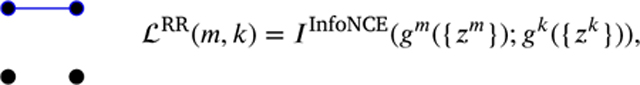


where we the objective ${ℒ}^{RR}(m,k)$ is defined for a pair of modalities m and k.

The fourth elementary objective is similar to *RR*, but only maximizes the mutual information between the two inter-modal local representations $c_{ld}^{m}$ and $c_{of}^{k}$, where d and f are arbitrary locations in layers l, o within modality m and k≠m, respectively. This objective is called Convolution-to-Convolution (CC) and is shown as the fourth from the top left in Fig. 2. The CC objective has been used in AMDIM (Bachman et al., 2019), CM-DIM (Sylvain et al., 2020b), and ST-DIM (Anand et al., 2019). Due to many possible pairs of locations between the activation maps in each encoder, we reduce the computational costs by sampling arbitrary locations, which was proposed in AMDIM (Bachman et al., 2019). Thus, after sampling an arbitrary location from the convolutional activation map for one modality, we compute the objective similarly to *XX*, by treating sampled locations as the global representation.

(6)

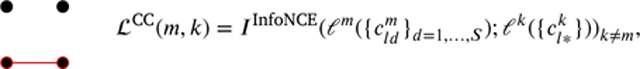


where *∼{1,…,T} is a sampled location from T locations. The objective ${ℒ}^{CC}(m,k)$ is defined for a pair of modalities m and k.

Combining these four primary objectives can construct more complicated objectives, as shown in Fig. 2. For example, the *XX-CC* objective for two modalities (as m=M1 and k=M2) can be written as

(7)

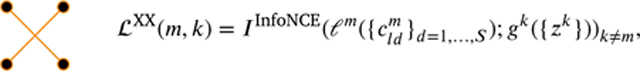


The goal would be to find parameters Θ that maximize ${ℒ}^{XX-CC}$. The objective is repeated with flipped modalities to preserve the symmetry of *XX* and *CC*. Removing the symmetry is intuitively similar to guiding the representations of one modality by the representations of another modality, which may be interesting for future work on asymmetric fusion. The XX-CC objective coordinates representations locally with the *CC* objective on convolutional activation maps and coordinates representations across scales in the encoder with *XX*. The local representations of one modality should be predictive of the global and local representations of the other modality.

### 2.5. Baselines and other objectives

We compare our method to an autoencoder (*AE*), a deep canonical correlation autoencoder (*DCCAE*) (Wang et al., 2015), and a supervised model. Each model type is a high-performing version of the three main categories of alternative approaches to our framework. The *AE* and supervised models are trained separately for each modality, while the *DCCAE* is trained jointly on all modalities. By *Supervised*, we refer to a unimodal model trained to predict a target using cross-entropy loss.

In addition to defining a unified framework that covers multiple existing approaches, our taxonomy contains a novel unpublished approach that combines different combinations of the four objectives. One novel approach combines the *CR* objective with the objective of the *DCCAE*, which we call *CR-CCA*. The *CR* objective allows us to train using modality-specific information, and the *CCA* objective aligns the representations between modalities. This leads to the following objective:

(8)

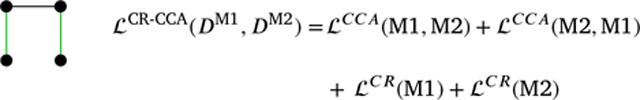



A second novel approach combines the *AE* objective with our *RR* objective to create the *RR-AE* objective. The *AE* objective ensures the learning of modality-specific representations, and the *RR* objective enforces the alignment of representations across modalities, similar to the *CCA* objective in the *DCCAE*. The final objective of the *RR-AE* is as follows.

(9)

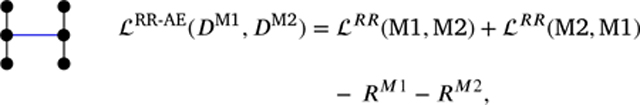


where $R^{M}$ is the mean squared reconstruction error for the AE with an additional decoder D, and modality M:

(10) $$R^{M}=\frac{1}{N}{\sum_{n=1}^{N}\left\|x_{n}^{M}-D^{M}\left(E^{M}\left(x_{n}^{M}\right)\right)\right\|}^{2}$$

**Fig. 3.**
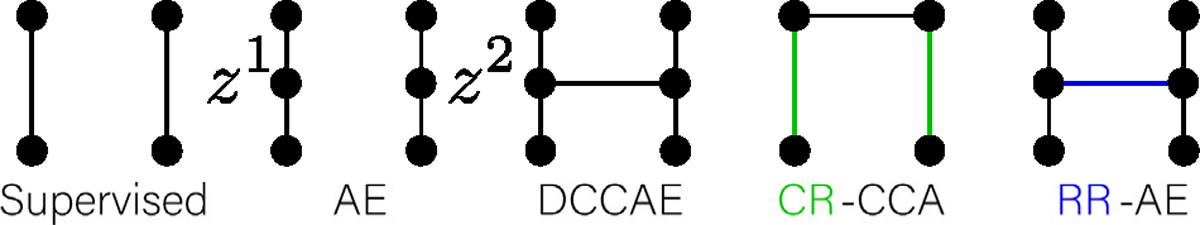
This figure shows schemes for the following models: *Supervised*, autoencoder (*AE*), deep canonical correlation autoencoder (*DCCAE*), the *CR* objective combined with *CCA* (*CR-CCA*), and the *RR* objective combined with an autoencoder (*RR-AE*). The Supervised and *CR-CCA* objectives follow a similar structure as schemes represented in our taxonomy; see Fig. 2. The AE-based models (*AE*, *DCCAE*, *RR-AE*) are represented by a 3-dot scheme where the middle dot is a representation, the lower dots are the input itself, and the upper dots are reconstructions of the input.

The baseline schemes are shown in Fig. 3. The AE-based models (*AE*, *DCCAE*, *RR-AE*) are represented by a 3-dot scheme where the middle dot is a representation, the lower dots are the input itself, and the upper dots are reconstructions of the input.

## 3. Experimental details

In this section, we provide a detailed description of the dataset, outline the implementation framework, and discuss the evaluation of the learned representation.

### 3.1. Dataset

In this study, we validate our method using the OASIS-3 (LaMontagne et al., 2019) dataset, a multimodal neuroimaging dataset comprising multiple Alzheimer’s disease phenotypes. Each subject within this dataset is represented by a T1 volume and a fractional amplitude of low-frequency fluctuation (fALFF) (Zou et al., 2008) volume, generated from T1w and resting-state fMRI (rs-fMRI) images. The T1 volume accounts for brain anatomy, while the fALFF volume captures resting-state dynamics. Both T1 and fALFF volumes have proven to be informative in studying Alzheimer’s disease (He et al., 2007) but also other conditions, such as chronic smoking (Wang et al., 2017).

#### 3.1.1. Preprocessing

The T1w images underwent brain masking using BET in FSL (Jenkinson et al., 2002) (v 6.0.20), linear transformation to MNI space, and subsampling to 3 mm following preprocessing. We discarded 15 T1w images due to their failure to pass the initial visual quality assessment. The rs-fMRI was registered to the first image using MCFLIRT in FSL (Jenkinson et al., 2002) (v 6.0.20), with specific parameters detailed in the original text. We computed the fALFF maps within the 0.01 to 0.1 Hz power band using REST (Song et al., 2011). Both modalities have a final volume size of 64 × 64 × 64.

We registered the volumes in the MNI space to simplify our method’s analysis and interpretability. However, we minimized data preprocessing to retain as much information from the original data as possible for the neural network to learn. Additionally, we subsampled to 3 mm to reduce computational demands, with applications on 1 mm earmarked for future work.

#### 3.1.2. Demographics and labels

The dataset’s largest cohort comprises non-Hispanic Caucasian subjects (84%). We selected 826 (70% HC, 15% AD, 15% unlabeled) Non-Hispanic Caucasian subjects. The OASIS-3 (LaMontagne et al., 2019) dataset contains a considerable number of subjects who are neither classified as AD nor readily identified as controls. These subjects belong to one of 21 diagnostic categories, such as cognitive impairment, frontotemporal dementia (FTD), Diffuse Lewy body disease (DLBD), and vascular dementia from preclinical cohorts, and followed longitudinal progression. We combined all such subjects into a distinct third class. In addition, to Non-Hispanic Caucasians, we used 134 African American subjects (100 HC, 34 AD) as an out-of-distribution hold-out set for transfer learning with linear evaluation.

#### 3.1.3. Dataset splits

We partitioned the 826 subjects into 5 stratified folds and 1 holdout test sets. The final subsets consist of 580–582 subjects (2828–2944 pairs) as training, 144 – 146 subjects (653 – 769 pairs) as validation, 100 subjects (424 pairs) as hold-out test. Our splits ensure that each subject is only present in one of the subsets. The details of the splits are shown in Table 1. We matched the scans of each modality based on the closest available date for each subject, resulting in a total of 4021 pairs for 826 subjects. The number of pairs exceeds the number of subjects because some subjects have multiple scans. We utilized all the pairs during pre-training but limited our final evaluation to one pair of images per subject. We opted to evaluate only one pair per subject to ensure that performance will not be biased towards subjects with more pairs. For the 2-way classification, long-tailed phenotypic data were excluded, while for the 3-way classification, we incorporated unlabeled data as a long-tailed phenotypic third class. We use an entire dataset of Non-Hispanic Caucasian subjects to perform model introspection for interpretability, but we still limit analysis to one pair per subject for the same reasons.

### 3.1.4. Data augmentation and additional preprocessing

Before introducing the images into the neural network, we normalized the intensities of the T1 and fALFF images using min–max rescaling to the unit interval ([0, 1]). During pre-training, we augmented the dataset by incorporating random crops of size 64, obtained after applying reflective padding of size eight to all sides. The preprocessing and augmentation choices were determined based on evaluations of the supervised baseline.

We also explored histogram standardization, z-normalization, random flips, and a balanced data sampler (Hermans et al., 2017). However, these alternatives did not yield significantly different results. Consequently, we employed a simple min–max rescaling and random cropping approach to minimize computational costs.

### 3.2. Framework

**Fig. 4.**
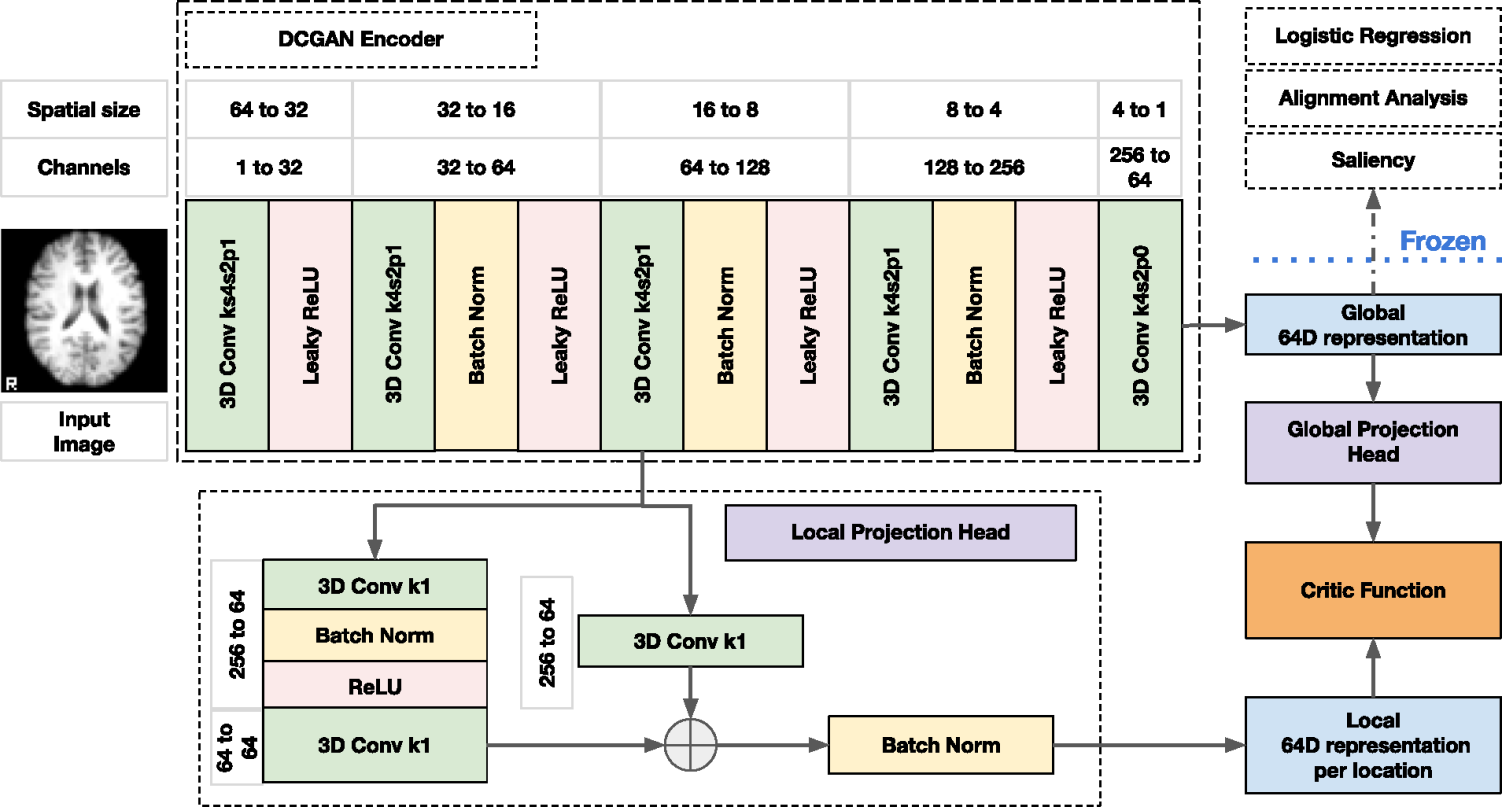
The Figure show the learning framework for the CR-based objective with the T1 image. It includes an encoder with DCGAN (Radford et al., 2015) architecture, local and global projection heads, and the computation of the critic function. The evaluation of the representation is performed with a frozen pre-trained encoder. First, with logistic regression, we evaluate the downstream performance. Secondly, with alignment analysis, we explore the multimodal properties of the representation. Finally, we interpret the representation in the brain space with saliency gradients.

Our experimental framework is depicted in Fig. 4, encompassing the preliminary pre-training, subsequent evaluation of the classification tasks, alignment of the representations, and analysis of the representations through the lens of saliency in brain space.

#### 3.2.1. Architecture

For our encoder, we choose the architecture from the *deep convolutional generative adversarial networks* (DCGAN) (Radford et al., 2015). This architecture provides a simple, fully convolutional structure and has a specialized decoder which is essential for the performance of generative and autoencoding approaches. We used volumetric convolutional layers for the experiments with neuroimaging OASIS-3 dataset. Most of the hyperparameters we left as in the original work (Radford et al., 2015). We swapped the last tanh activation functions in the decoder with a sigmoid because the intensities of the input images are scaled into the unit interval. The last layer projects activations from the previous layer to the final 64-dimensional representation vector that we named *global*. All convolutional layers are initialized with Xavier uniform (Glorot and Bengio, 2010) and gain related to the activation function (Paszke et al., 2019). Each modality has its encoder with DCGAN architecture.

The local projection head is needed to project *local* representation to a 64-dimensional channel space to ensure that the critic scores between the *global* and *local* representations will be computed in the same space. For a *local projection head* ℓ, we choose an architecture similar to AMDIM (Bachman et al., 2019) but uses volumetric convolutional layers. The projection head represents one ResNet block from a third *l* = 3 layer of DCGAN architecture with feature size 128 × 8 × 8 × 8. One direction in the block consists of 2 convolutional layers (kernel size 1, number of output and hidden channels 64, Xavier uniform initialization (Glorot and Bengio, 2010)). The second direction consists of one convolutional layer (kernel size 1, number of output channels 64, initialization as identity). The projection heads are individual for each modality, and we added them only if the model uses convolutional features in the objective.

The global projection head is needed to improve the representation. As it has been shown (Chen et al., 2020), the last layer of the neural network can develop a lower rank condition which is beneficial to the optimization of the objective but can be destructive to the representation. For a *global projection head g*, we follow SimCLR (Chen et al., 2020). We perform a hyperparameter search for the number of hidden layers in the projection head for each model that can use the projection head (except *Supervised*, *AE*, *CC*). We have considered cases: without a projection head, with a linear projection head, and a projection head with 1-, 2-, or 3-hidden layers. The number of output dimensions in the projection layers equals 64.

#### 3.2.2. Optimization and regularization for pre-training

We perform the training of the models on OASIS-3 dataset with RAdam (Liu et al., 2019) optimizer with learning rate (lr=4e−4). The pre-training step in our framework has been performed for 500 epochs. For each trained model, we saved 10 checkpoints based on the best validation loss.

In addition, following AMDIM (Bachman et al., 2019), we add regularization to InfoNCE objective by penalizing the squared scores computed by the critic function as $\lambda f(x,y{)}^{2}$ with λ=4e-2, and clipping the scores by $c\;tanh(\frac{s}{c})$ with c=20.

### 3.3. Evaluation

It is important to exhaustively validate the representations the model learns, which in the proposed framework is done in three steps. The first step evaluates the representations using classification tasks with logistic regression. This step uses the features extracted by the encoder from the input. This evaluation aims to ensure the discriminative power of the pre-trained features. In the second step, we compute the similarity between representations to measure how much joint information has been captured by the model. The third and last step consists of two analyses to explore the relationship between the latent space and the brain space to assess voxel-wise group differences based on saliency gradients for each of the *d* dimensions of the representation.

#### 3.3.1. Classification evaluation

After the pre-training step, to evaluate the discriminative performance of the representations captured by the model, we train logistic regression on frozen representations from the last layer of the encoder. Note that most self-supervised learning algorithms evaluate the discriminative power of representations with a linear evaluation protocol based on linear probes (Alain and Bengio, 2016). However, we chose to use logistic regression due to faster training times.

**Fig. 5.**
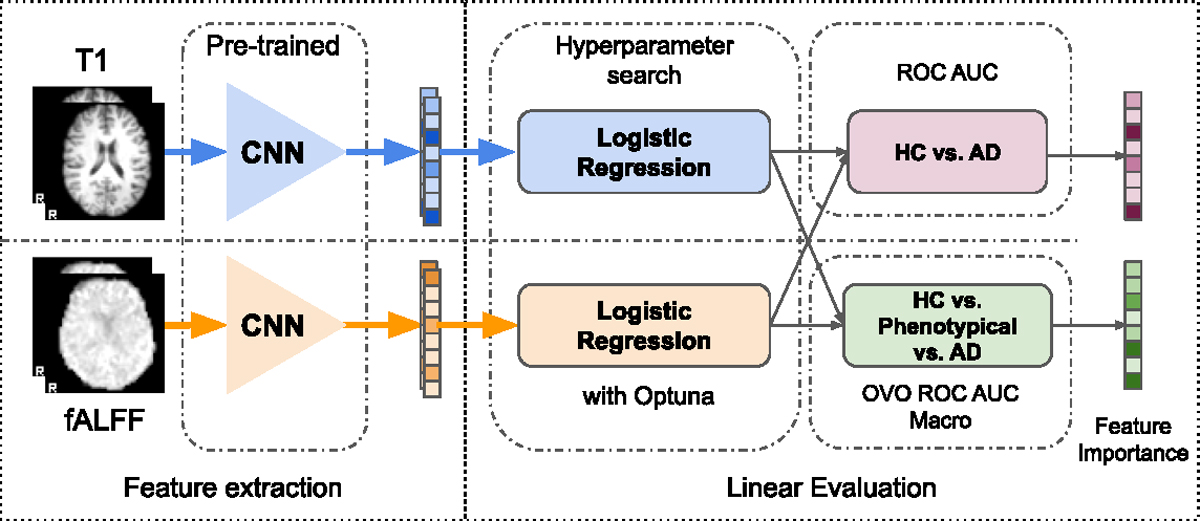
The schematic process of linear evaluation with logistic regression. In the first stage, we perform feature extraction using a pre-trained encoder. In the second stage, we search for a hyperparameter based on a validation set using Optuna (Akiba et al., 2019). After finding parameters, we compute metrics for each task and save importance scores of the features (beta values of the logistic regression) for further analysis.

The process of linear evaluation with logistic regression is shown in Fig. 5. We perform two classification tasks to evaluate the discriminative performance of representations learned by the various objectives in our taxonomy. The first task is a binary classification of Alzheimer’s Disease (AD) vs. Healthy Cohort (HC). The second task is a ternary classification with an additional long-tailed phenotypical class. The first task is easier than the second one because the latter has an added class.

After extracting the representations with a pre-trained encoder, the logistic regression is trained on *global* representation *z*. We use logistic regression (from scikit-learn Pedregosa et al., 2011) to perform classification tasks. The hyperparameters of the logistic regression were optimized with Optuna (Akiba et al., 2019) for 500 iterations. The selections of the hyperparameters are performed based on the validation subset. The search space for hyperparameters is defined as follows: inverse regularization strength *C* is sampled log-uniformly with interval [1e−6,1e+3], for the elastic net penalty, the mixing parameter is uniformly sampled from unit interval [0,1]. The logistic regression is trained using *SAGA* solver (Defazio et al., 2014). We use a ROC AUC and one-vs-one (OVO) ROC AUC Macro (Hand and Till, 2001) as a scoring function for a hyperparameter search for binary and ternary classification, respectively. The OVO strategy for ROC AUC metrics in multiclass classification is computed as the average AUC of all possible pairwise combinations of classes. In addition, it is insensitive to class imbalance for macro averaging (Pedregosa et al., 2011). Classification is performed separately for each modality by training logistic regression on the representations extracted from that modality using the corresponding convolutional encoder.

#### 3.3.2. Alignment analysis

**Fig. 6.**
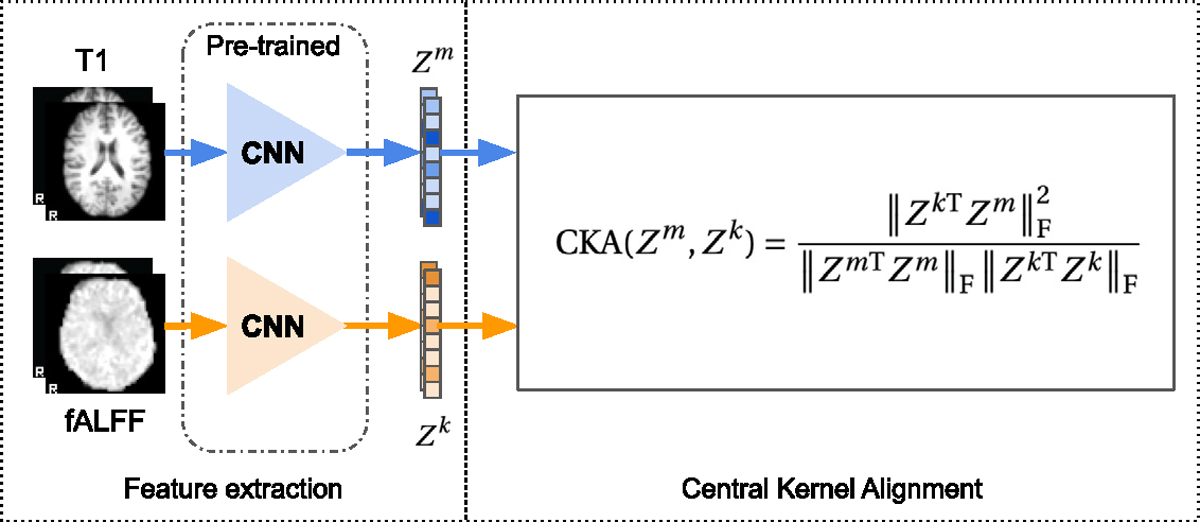
The schematic process of computing Central Kernel Alignment (CKA). In the first stage, we perform feature extraction using a pre-trained encoder. In the second stage, we stack features in matrices n×d where d is the dimensionality of the representation vector, and n is the number of samples.

The alignment analysis is added to estimate the joint information content of the representation between modalities as a measure of the inductive bias of the training objective. We use central kernel alignment (CKA) (Kornblith et al., 2019) to evaluate the alignment between representations of different modalities. CKA is effective (Kornblith et al., 2019) as a method to identify the correspondence between representations of networks with different initializations, compared to CCA-based similarity measures (Raghu et al., 2017; Morcos et al., 2018). CKA is considered to be a normalized version of the Hilbert–Schmidt Independence Criterion (HSIC) (Gretton et al., 2005). The CKA measure for a pair of modalities m and k is defined as:

(11) $$\mathrm{CKA}\left(Z^{m},Z^{k}\right)=\frac{{∥Z^{kT}Z^{m}∥}_{F}^{2}}{{∥Z^{m\;T}Z^{m}∥}_{F}{∥Z^{k\;T}Z^{k}∥}_{F}},$$

where d is the dimension of the latent representation, Z is a n×d matrix of stacked global d-dimensional representation z for n samples, $∥\cdot {∥}_{F}$ is the Frobenius norm. The schematic process of computing CKA is shown in Fig. 6.

Our results are only evaluated using CKA since we find it (Fedorov et al., 2021) to be the most robust to noise, which reinforces findings in previous literature (Kornblith et al., 2019; Nguyen et al., 2021) that suggest the same.

#### 3.3.3. Saliency explanation of the representation in brain space

To explain the representations in brain space, we adapt the integrated gradients algorithm (Sundararajan et al., 2017). We want to understand the representations rather than the saliency of a specific label. Hence we propose a straightforward adaptation. Instead of using a target variable, we compute gradients with respect to each dimension of the representation. This is done by setting the specific dimension in the vector to 1 and all other dimensions to 0.

#### 3.3.4. Model selection

We save the top 10 checkpoints during training based on the validation loss. In contrast, most self-supervised studies save checkpoints based on the classification performance using a proxy classifier during training. We use only the loss and do not rely on the labels. Selecting the best ten checkpoints is a valid strategy as we ensure that our models converge within 500 epochs (see learning curves https://wandb.ai/noidea/neuroimage-fusion/). Moreover, there is no guarantee that lower self-supervised loss leads to better classification performance.

For each modality, we extract representation separately. However, the checkpoint is the same for both modalities in multimodal models. Because otherwise, the alignment measure with CKA will be between two unrelated models. For unimodal models, the checkpoints are paired based on the saving order to ensure that the model trained relatively with the same number of epochs.

**Fig. 7.**
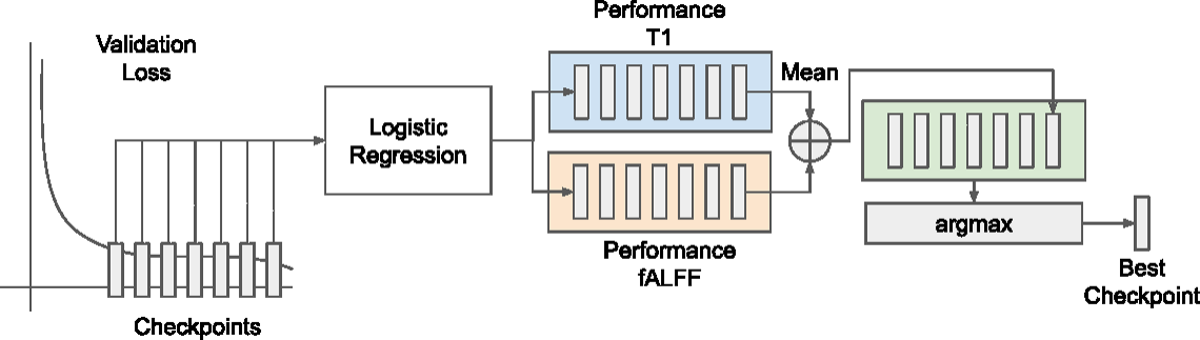
The schematic process of best checkpoint selection.

After extracting features, we train the logistic regression for each checkpoint by tuning its hyperparameters via optuna (Akiba et al., 2019) on a validation set. After calculating each modality’s logistic regression performance score, we computed the average performance across two modalities for that checkpoint. Then we select the best checkpoint (epoch) as a maximum over checkpoint’s performance (e.g., the ROCAUC or OVO ROCAUC Macro (based on the task)). The procedure is shown in Fig. 7.

Once we select the best checkpoint, we compute mean over folds to select the hyperparameter set of the model (e.g., projection head) based on a validation set. Finally, we report the hold-out test set performance of the model.

## 4. Results

In this section, we present our findings derived from two classification tasks within the OASIS-3 dataset, with an emphasis on examining the comparative performance of self-supervised methodologies. The *Supervised* model serves as the upper bound performance when labels are accessible during training. We have implemented baseline models such as unimodal models *AE* and *CR* (Fedorov et al., 2019), along with multimodal models *DCCAE* (Wang et al., 2015), *RR* (SimCLR) (Chen et al., 2020), and *XX-CC* (AMDIM) (Bachman et al., 2019). Moreover, we delve into the inductive biases of various multimodal objectives using the Centered Kernel Alignment (CKA) metric to evaluate the joint information contained within the cross-modality representations. Subsequent to this, we highlight the group differences between the supervised models and the top-performing self-supervised models established initially. Lastly, we explore the multimodal associations between representation T1 and fALLF.

### 4.1. Linear classification performance

**Fig. 8.**
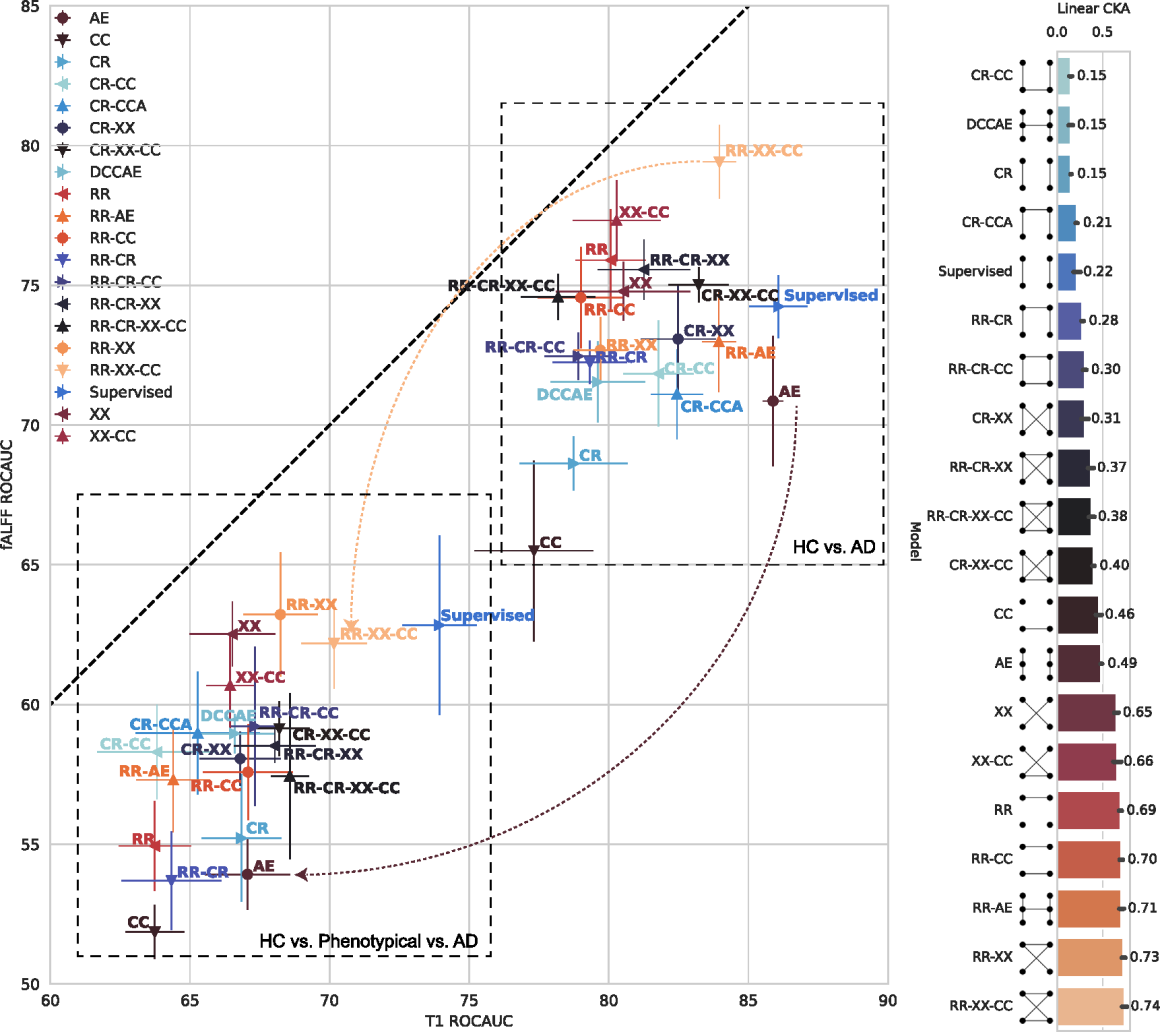
The figure shows ROC AUC performance of logistic regression on a hold-out test set for binary classification (top right corner) and ternary classification (bottom left corner) tasks. On the right side, the barplot shows CKA on a hold-out test set across the subject for ternary classification. The color map of the legend corresponds to ranking with CKA. Markers correspond to the mean of the ROC AUC, and error bars correspond to the standard error. The X- and Y -axis correspond to the ROC AUC on T1 and fALFF modalities. The ROC AUC was measured as a one-versus-one (OVO) macro metric for ternary classification. Two classification tasks are shown on the same plot to visualize the generalizability of the learned representation in tasks with different difficulties. The dashed line represents a diagonal of the balanced performance between T1 and fALFF. The CKA shows an alignment of the representation between modalities as a measure of joint information. Lower CKA values mean less joint information between representations, and higher CKA values — more joint information. The brown arrow shows how the relative ranking of the *AE* model falls on a different task. The pale orange arrow shows the consistency of the relative ranking for the *RR-XX-CC* model.

The classification results on a hold-out test set are shown for both tasks in Fig. 8. The performance is shown with a mean and standard error of the ROC AUC and one-versus-one (OVO) ROC AUC Macro (average) (Hand and Till, 2001) metrics for binary and ternary classification tasks, respectively. Additionally, we report CKA as the measure of joint information between representations of different modalities for each model. In Fig. 8, CKA was computed on all subjects for three classes from the hold-out test set.

The best-performing model for binary classification for T1 modality is Supervised with 86.1 ± 1.0%. However, three models are comparable based on the statistical comparison (Table 2). Two of them are multimodal *RR-XX-CC* 84.0 ± 0.6% and *RR-AE* 84.0 ± 0.6%, and one of them unimodal *AE* 85.9 ± 0.3%.

The best-performing model on ternary classification for T1 modality is Supervised 73.9±1.3%. However, there are a couple of self-supervised multimodal models that are very close where the *p*-value is only *p* < 0.1 (Table 4). However, the strongest self-supervised multimodal model is *RR-XX-CC* with 70.2 ± 1.2% ROCAUC.

The best-performing model for binary classification for fALFF modality is *RR-XX-CC* with 79.4 ± 1.3%. Based on statistical comparison (Table 3) it shares the first place with *XX-CC* 77.3 ± 1.4% and *RR* 75.9 ± 1.8%.

On ternary classification for fALFF modality, the best performing model is *RR-XX* 63.2 ± 2.2%. Based on statistical comparison (Table 5) it is comparable with *Supervised* 62.9±3.2%, *XX* 62.5±1.2%, *RR-XX-CC* 62.2±1.6%. The significance comparison test marks many other models in Table 5. We hypothesize that the main reason for many comparable models is a high variance of the model’s performance in this task which can be explained by the difficulties of training models on fALFF with limited data.

The unimodal autoencoder (*AE*) model can perform well on the simple binary classification task and T1 as it achieves the best performance as an unsupervised model 85.9±0.3%. However, the performance significantly drops (brown arrow in Fig. 8) on the harder ternary classification task with long-tailed third class for T1, achieving 67.1±1.5%, which is worse than multimodal models such as *RR-XX-CC* (70.2±1.2%). Unimodal models such as *CR* and *AE* are outperformed by most of the multimodal models, especially on fALFF. A multimodal extension of *AE* with *CCA* as *DCCAE* or *RR-AE* can improve performance on classification tasks. However, these models also struggle with consistency of the results as *AE*.

*CCA*-based models (*CR-CCA*, *DCCAE*) consistently outperformed most of the proposed self-supervised decoder-free models on 2-way and 3-way classification tasks. Therefore we could achieve more robust performance with the proposed models while reducing the computational cost and the number of parameters in the models by not requiring the decoder for each modality.

Overall, the proposed self-supervised models, specifically *RR-XX-CC*, perform robustly on both tasks and retain their ranking relative to the other models. Significantly, these models outperform previous unsupervised unimodal and multimodal models. In addition, these results support the benefits of multimodal learning as co-learning, which could be seen as a regularization effect.

Judging by the higher CKA alignment measure (0.63 – 0.74), these models capture joint information between modalities. While there are other models—*RR-AE*, *RR-CC*, and *RR*—that achieve higher CKA alignment, they are not as robust. We hypothesize that the joint information alone is not the answer to the problem, but the architecture of the model is essential. Note that the *XX*, *XX-CC*, *RR-XX*, and *RR-XX-CC* models capture the local–global relationship across modalities. While the *RR-AE*, *RR-CC*, and *RR* models only capture joint information on global–global or local–local representation level. Thus, given the empirical evidence in Fig. 8, the local–global relationship *XX* is an essential building block for multimodal data because it allows us to capture complex multi-scale relationships between modalities. Importantly, CCA-based approaches such *DCCAE* and *CR-CCA* fail to capture high alignment per the CKA measure.

By taking into account the classification performance (near supervised performance on T1 on both tasks, best performance on fALFF for binary classification, and near best performance on ternary classification), consistency of the performance (pale orange arrow in Fig. 8) and the amount of captured joint information, we select *RR-XX-CC* for further analysis in this manuscript.

#### 4.1.1. Label efficiency and transfer learning

Here, we explore the label efficiency (Jin et al., 2023) of the models with binary classification tasks performed specifically on Non-Hispanic Caucasian and African American subjects. The model was exclusively trained on Non-Hispanic Caucasians, meaning the African American dataset represents an application of transfer learning to a subset that is out of distribution.

To refresh, SSL models are trained in two stages: initial pre-training on the unlabeled datasets followed by linear evaluation on the labeled dataset. To evaluate the label efficiency (Jin et al., 2023) we vary the labeled dataset in the second stage by using only a subset of the labels. We train *Supervised* model using that subset of the labels, while self-supervised does not need labels, hence trained on the full unlabeled training dataset. Because of the ability to train models on unlabeled data, SSL has access to more samples than the Supervised. Hence comparing the performance of the SSL with respect to Supervised can only show the label efficiency, but not the data efficiency. As data efficiency would require to train SSL on similar smaller subsets.

Guided by the above, we train our SSL models on the whole training subset, while we train Supervised only on the subset with the available labels. We control the number of labels in the training subset in each fold by the percentage of the subjects while ensuring stratified label distribution. Specifically, we use 10%, 25%, 50%, 75%, 100%. After pretraining all models, we follow the same linear evaluation protocol with Logistic Regression, where the encoder is frozen and serves as a feature extractor.

**Fig. 9.**
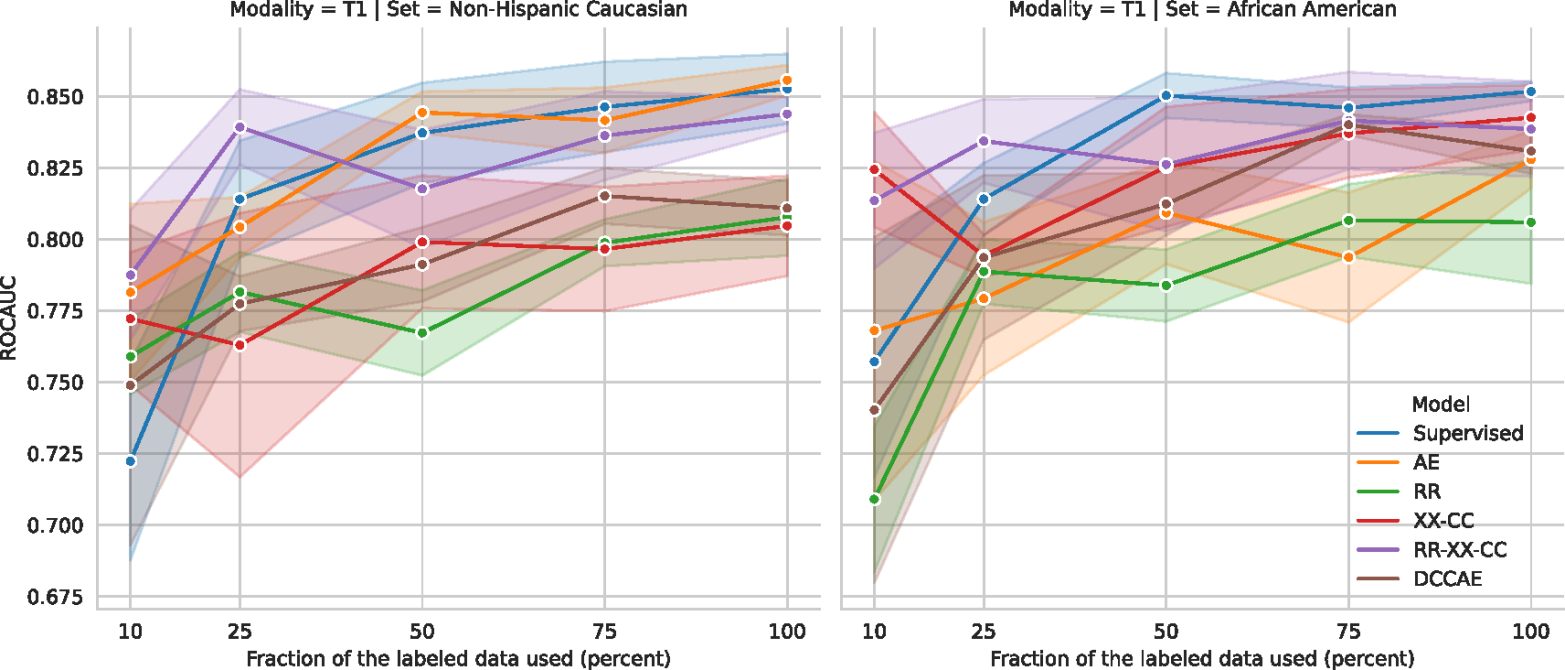
Label efficiency on T1 of the six best models (*Supervised*, self-supervised baselines, and best proposed) on binary classification on the hold-out test set with Non-Hispanic Caucasian subjects and for transfer learning to the out-of-distribution set with African American subjects. The percent corresponds to the number of subjects with labels: 10%,25%,50%,75%,100%. The performance is measured with ROCAUC using mean and standard error.

**Fig. 10.**
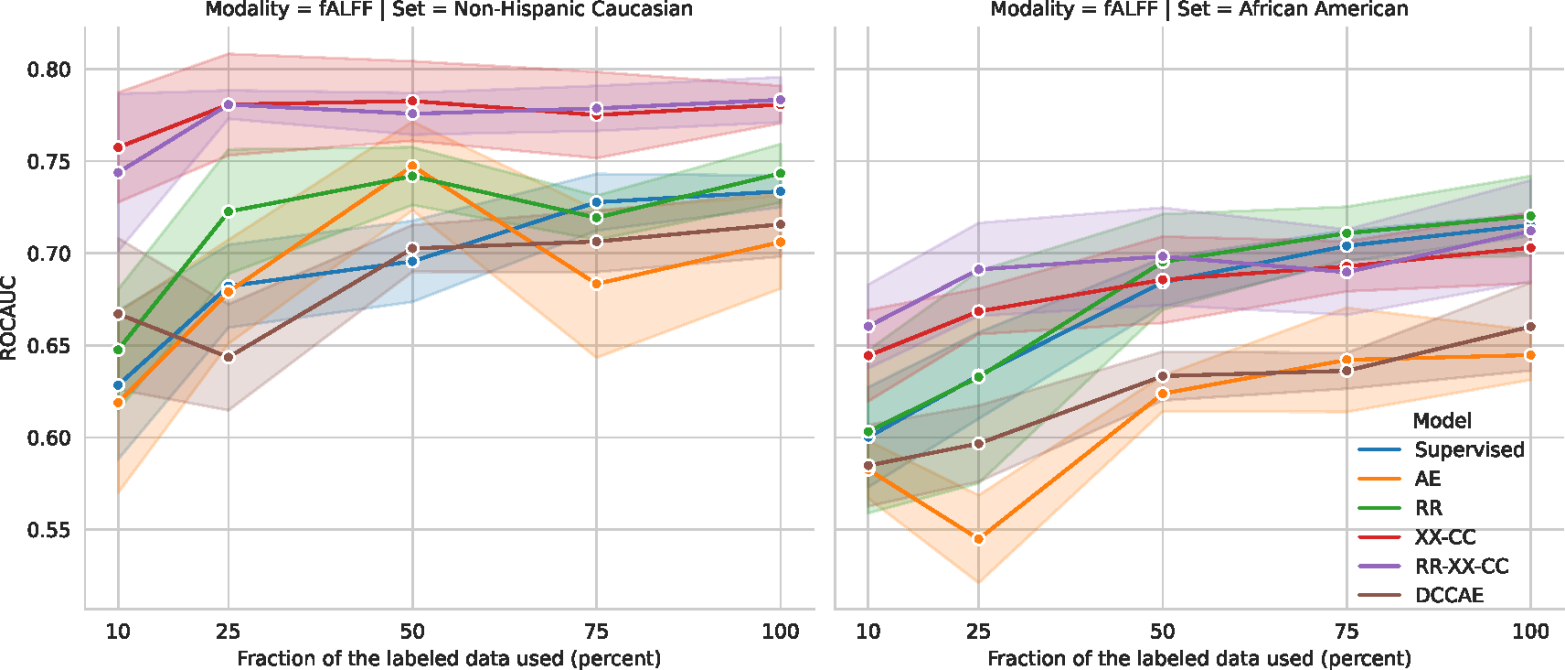
Label efficiency on fALFF of the six models (*Supervised*, self-supervised baselines, and best proposed) on binary classification on the hold-out test set with Non-Hispanic Caucasian subjects and out-of-distribution set with African American subjects. The percent corresponds to the number of subjects with labels: 10%,25%,50%,75%,100%. The performance is measured with ROCAUC using mean and standard error.

The results are shown in Fig. 9 on T1 and Fig. 10, and the detailed metric values are shown in Table 6 for T1 and in Table 7 for fALFF.

For the label-efficiency, the *Supervised* model starts to catch up with the best self-supervised model *RR-XX-CC* after being trained with more than 25% of the labeled data on T1. While on fALFF, the *Supervised* cannot catch the performance of the multimodal models *RR-XX-CC* and *XX-CC*. This result shows the benefit of multimodal learning for fALFF.

For transfer learning, all models drop performance on fALFF with a maximum difference of 13.4%, while on T1, the models mostly perform comparably, and the maximum difference is 5.5%. If we conduct the Wilcoxon signed-rank test with the alternative hypothesis that “the performance of Non-Hispanic Caucasian subjects is greater than that of African American subjects”, we find significant drops in performance for the fALFF modality. A star marks a significant drop in performance. The drop on fALFF shows that the models cannot generalize to a different race, while it seems T1 almost does not have this issue. Furthermore, these results show that self-supervised and supervised learning cannot guarantee out-of-distribution generalization for free. Hence additional methodologies are needed. At least, the models should be able to access the data from a different race.

### 4.2. Interpretability

#### 4.2.1. Explaining group differences between HC and AD

In this subsection, we explain the performance of the models by analyzing the saliency maps. As a point of interest and comparison, we selected the models in the previous section *Supervised* and *RR-XX-CC*. We use these two models to generate saliency maps and interpret what the models have learned.

**Fig. 11.**
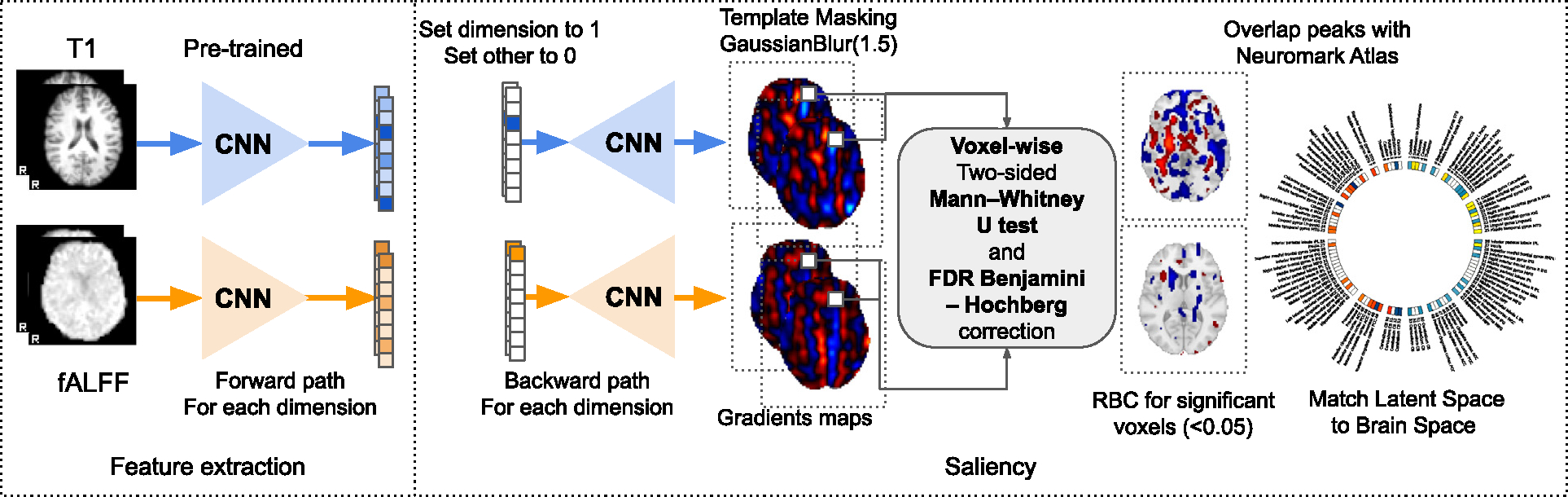
The schematic process for explaining group differences. First, we extract representations and compute gradients for each dimension of the representation vector. After that, we apply Gaussian Blur with σ=1.5 and brain mask. Then we perform a voxel-wise two-sided Mann–Whitney U test and correct p-values via FDR Benjamini–Hochberg correction with 0.05. We compute RBC as effect size to get final values for each voxel, and after selecting significant (α=0.05) voxels, we achieve RBC maps (total 64 maps (1 map for each of the 64 dimensions)). Finally, we overlap RBC maps with the Neuromark atlas by taking the *sign*(*max*(*abs*)) value found within ROI.

**Fig. 12.**
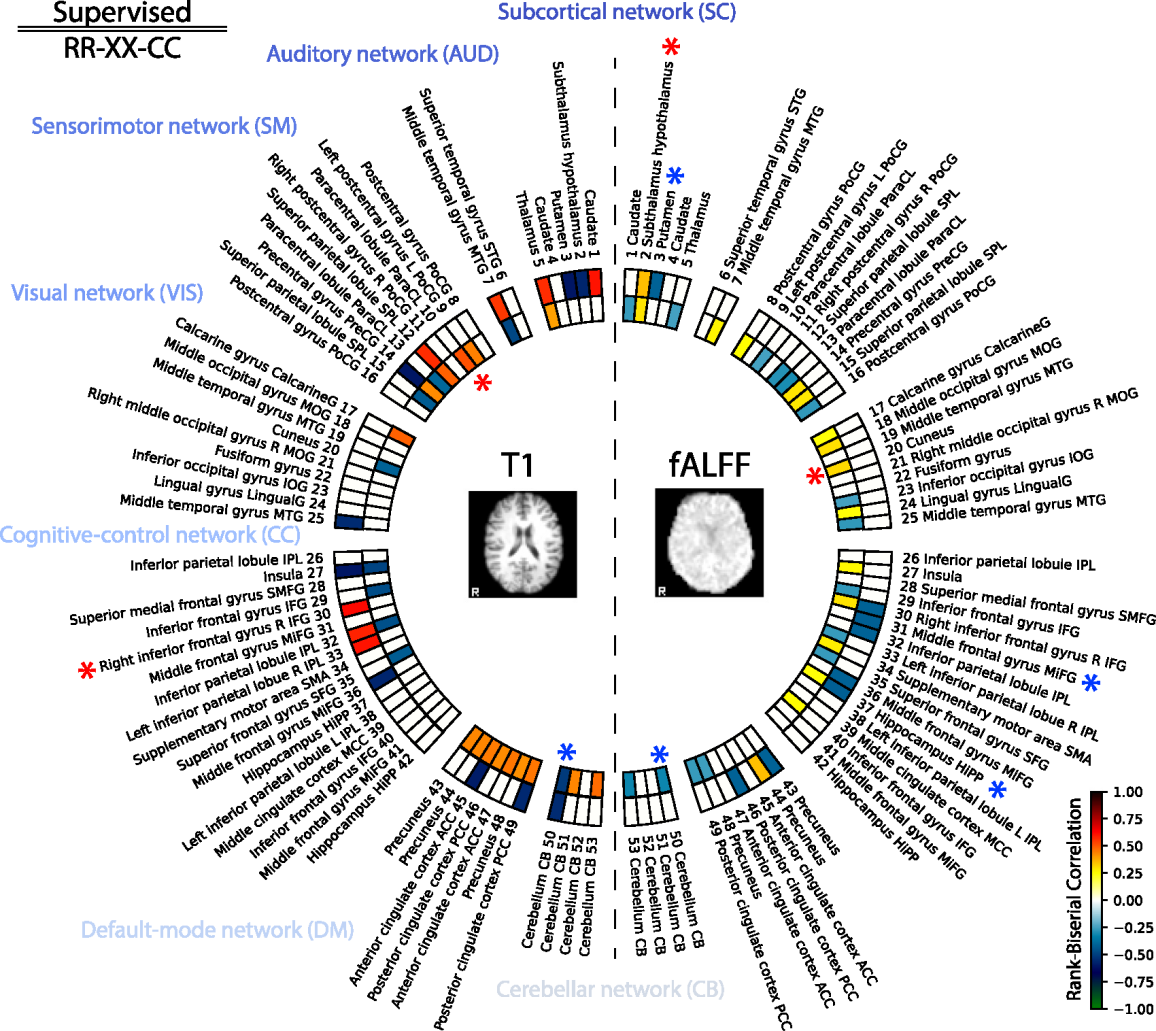
The figure shows the near absolute maximum (quantile 0.999) Peak RBC values of the saliency for regions in the Neuromark atlas for the dimensions with the highest positive and negative importance (Betas in Logistic Regression) derived from the best fold on binary classification. The left side shows values for T1 data, and the right side shows the maps found for fALFF. The outer circle shows values for the *Supervised* model and the inner circle for the *RR-XX-CC* model. Stars mark regions with the highest positive (red) and negative (blue) RBC value. The exact values are shown in Table 8.

For each selected model, we compute integrated gradients (Sundararajan et al., 2017) along each dimension in the 64-dimensional representation and keep both the positive and the negative parts of the gradients. After computing saliency gradients for each dimension of the latent representation, we apply brain masking and smooth them with a Gaussian filter (*σ* = 1.5). Then, we perform a voxel-wise two-sided test using the Mann–Whitney U-Test, with the Rank Biserial Correlation (RBC) used to quantify the effect size. We study interpretability based on these effect size maps. We call them RBC maps. The positive value of the RBC suggests that the neural network is more sensitive in that location for subjects with Alzheimer’s disease, while the negative values — less sensitive. The final RBC maps are achieved by selecting the significant voxels with a *p* < 0.05. However, before, we performed FDR Benjamini–Hochberg correction with a *p* < 0.05, as we ran multiple tests for each voxel. Lastly, we match the RBC maps with the ROIs defined in the template from the Neuromark pipeline (Du et al., 2020). We call this template the Neuromark atlas in the rest of the text. To create the Neuromark atlas, 53 spatial ICA components from the Neuromark template are used. To match RBC maps to Neuromark, we select the peak RBC value within spatial overlap for each ROI map in the atlas. The schematic framework for this analysis is shown in Fig. 11. The final results are summarized for all dimensions in Fig. 12.

As depicted in Fig. 12, a comparative analysis is presented between the *Supervised* model and the multimodal self-supervised model *RR-XX-CC*. The insight from this comparison is the evident dominance of the *Supervised* model in terms of peak values, represented by brighter colors, thus demonstrating a stronger contrast compared to *RR-XX-CC*. This investigation implies that the *Supervised* models exhibit more confident behavior due to higher RBC values. In contrast, the self-supervised model, *RR-XX-CC*, exhibits lower RBC and smoother behavior across brain regions. This is presumably because self-supervised models are designed to capture as much information as possible from the input images (Hjelm et al., 2019) to perform instance discrimination (Wu et al., 2018). The saliency maps of the *Supervised* model exhibit sparse yet potent peaks, which can be attributed to the model’s usage of labels to learn its representations.

For the *Supervised* model, *Putamen 3* (Coupé et al., 2019) displays the lowest RBC value of −0.607 on T1. *Right inferior frontal gyrus R IFG 30* (Chen et al., 2023; Hallam et al., 2020) exhibits the highest RBC value of 0.619 on T1. Other noteworthy peak RBC values include: *Subthalamus hypothalamus 2* (Zhu et al., 2019; Ríos et al., 2022) with a value of −0.564. *Precentral gyrus PreCG 14* (Behfar et al., 2020) at −0.604. *Thalamus 5* (Coupé et al., 2019) at 0.566. *Superior parietal lobule SPL 12* (Prawiroharjo et al., 2020; Corriveau-Lecavalier et al., 2020) at 0.569.

The *Supervised* model has the highest RBC value on fALFF, which is observed in *Subthalamus hypothalamus 2* (Zhu et al., 2019; Ríos et al., 2022) at 0.346. *Middle frontal gyrus MiFG 31* (Chen et al., 2023) and *Hippocampus HiPP 28* (Yang et al., 2022) both display the lowest value at −0.415.

In *RR-XX-CC* model for T1 modality, *Cerebellum CB 50* (Kolbeinsson et al., 2020) has lowest peak RBC at −0.508. *Paracentral lobule ParaCL 10* (Jang et al., 2017) exhibits the highest RBC value at 0.511. Other significant peak RBC values are: *Thalamus 5* (Coupé et al., 2019) at 0.398, *Left postcentral gyrus L PoCG 9* (Zhang et al., 2020) at 0.442, *Insula 27* (Philippi et al., 2020) at −0.488.

Interestingly, *RR-XX-CC* has high localization for T1 in the defaultmode networks that have been claimed to distinguish Alzheimer’s disease from healthy aging (Greicius et al., 2004). In addition, the sensorimotor network in both T1 and fALFF is studied for mild cognitive impairment and Alzheimer’s disease (Agosta et al., 2010; Sendi et al., 2021).

On fALFF, the highest saliency value is seen in *Cuneus 20* (Wu et al., 2021) at 0.308. *Cerebellum CB 50* (Kolbeinsson et al., 2020) displays lowest RBC value of −0.316. Additionally, other important peak RBC values include: *Subthalamus hypothalamus 2* (Zhu et al., 2019; Ríos et al., 2022) at 0.294, *Superior parietal lobule SPL 15* (Prawiroharjo et al., 2020; Corriveau-Lecavalier et al., 2020) at 0.283, *Right inferior frontal gyrus R IFG 30* (Chen et al., 2023; Hallam et al., 2020) at 0.289.

**Fig. E.20.**
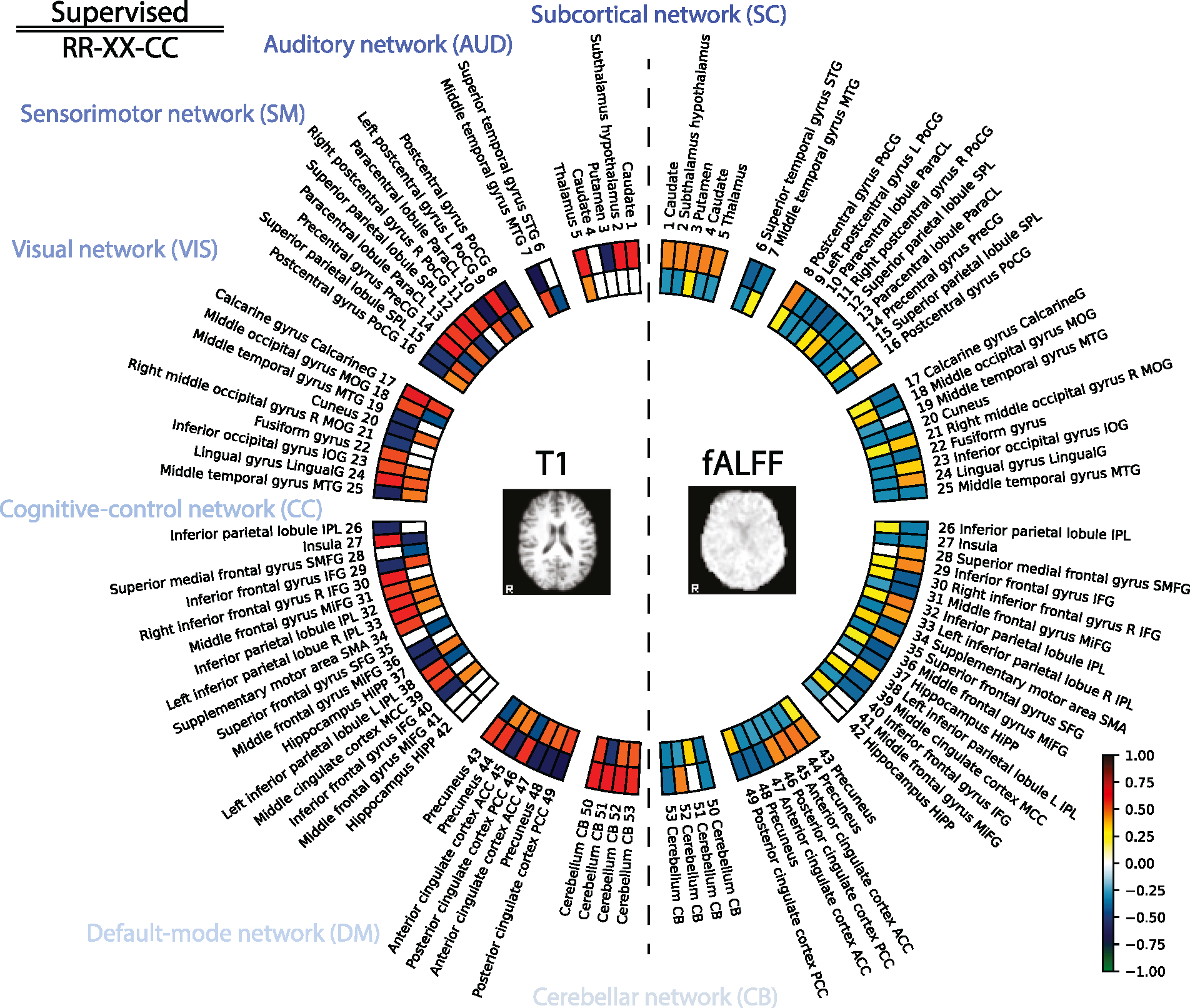
The figure shows the near absolute maximum (quantile 0.999) Peak RBC values of the saliency for regions in the Neuromark atlas for the dimensions with non-zero importance (Betas in Logistic Regression) derived from the best fold on binary classification. The left side shows values for T1 data, and the right side shows the maps found for fALFF. The outer circle shows values for the *Supervised* model and the inner circle for the *RR-XX-CC* model. Stars mark regions with the highest positive (red) and negative (blue) RBC value. The exact values are shown in Table E.16.

This gives a more detailed overview of each region found by the *Supervised* and *RR-XX-CC* models in both the T1 and fALFF modalities. This analysis is limited to the dimensions with highest and lowest importance. To see analysis across all the dimensions with non-zero importance see Fig. E.20 and Table E.16. Hence, further investigation and cross-referencing with related studies would help to understand the implications of these data.

#### 4.2.2. Exploring multimodal links

**Fig. 13.**
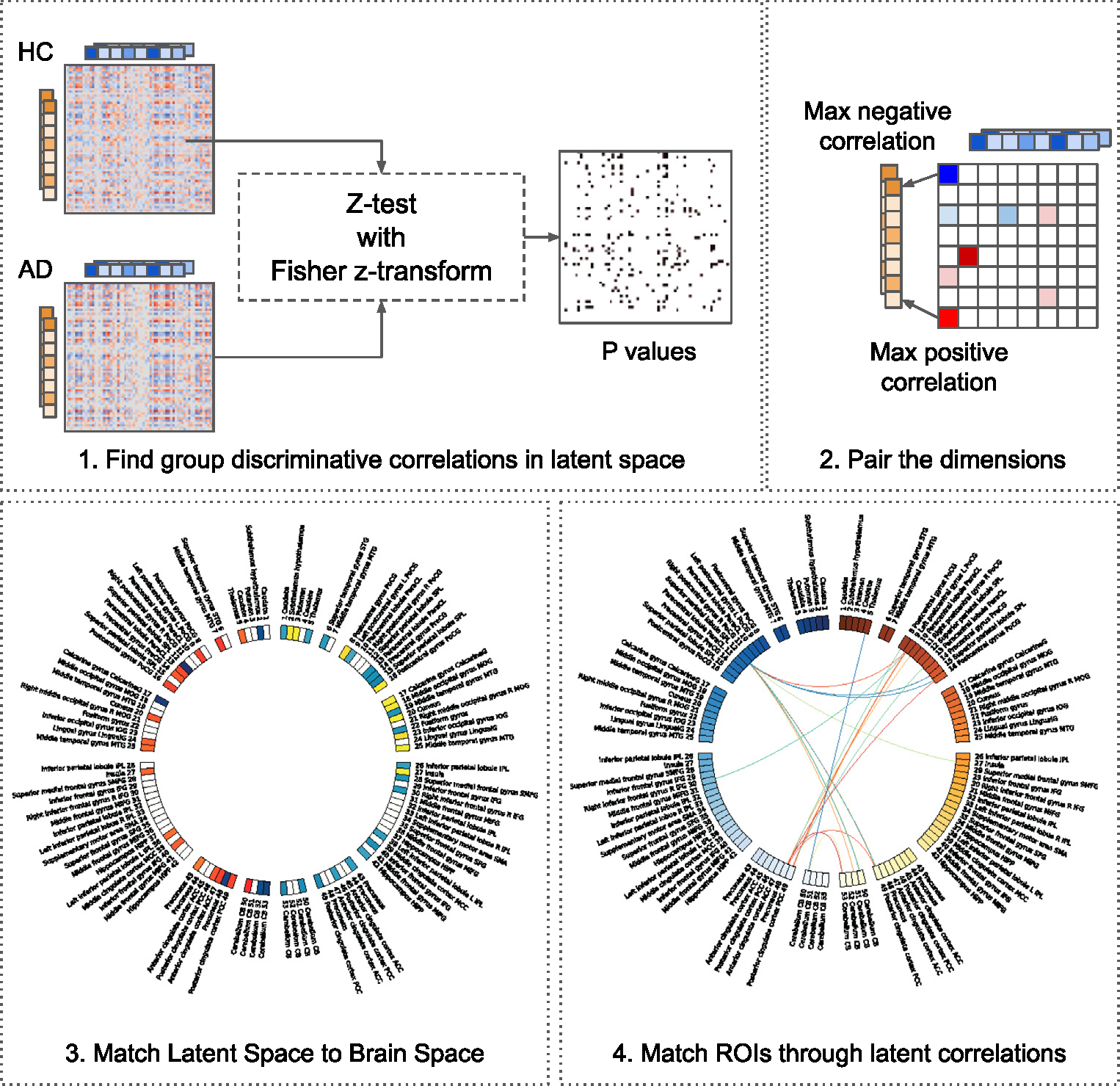
The schematic process for exploring multimodal links. First, we compute the Spearman’s correlation matrix for each group: HC and AD. We apply FDR Benjamini–Hochberg correction to select significant correlations within the group. After that, we perform Z-test with Fisher z-transform to find group-discriminative correlations in the latent space. Based on these results, we select significant correlations (α<0.05, shown as black in the figure, white are non-significant correlations). Then for each dimension, we find pairs of dimensions with the highest positive and negative correlation. The third step has been done in the previous section. Hence we reuse our result. However, we select only the ROI with the highest RBC peak value for analysis for the dimensions in the selected pair. Finally, we match top ROI labels in one modality via latent correlations with top ROIs in the other modality.

This section explores multimodal links between the T1 and fALFF modalities. To perform this analysis, we compute an asymmetric correlation matrix between all pairs of dimensions in 64-dimensional *global* representation of the T1 and fALFF. We computed the Spearman correlation, applied Benjamini/Hochberg FDR Benjamini–Hochberg correction for the two-sided hypothesis, and used *p* < 0.05 to select the links. We perform this separately for each of the groups: HC and AD. After that, given the correlation matrix for each group, we perform a z-test with Fisher z-transformed correlations to find significant group-discriminative correlations. Then, we pair dimensions across modalities. To create a pair, for each dimension in one modality, we find the dimension in the other modality with the highest positive and negative significant correlation. Since each dimension has already been assigned to multiple ROIs, we keep only the top ROI based on the peak RBC value for analysis. Finally, we connect the top ROI in one modality with the top ROI in another via an edge with a correlation value between dimensions as a weight. This methodology allows us to explore multimodal links in brain space through the latent space. We repeat the same procedure for each dimension from 64 dimensions and each modality. The schematic process is shown in Fig. 13.

**Fig. 14.**
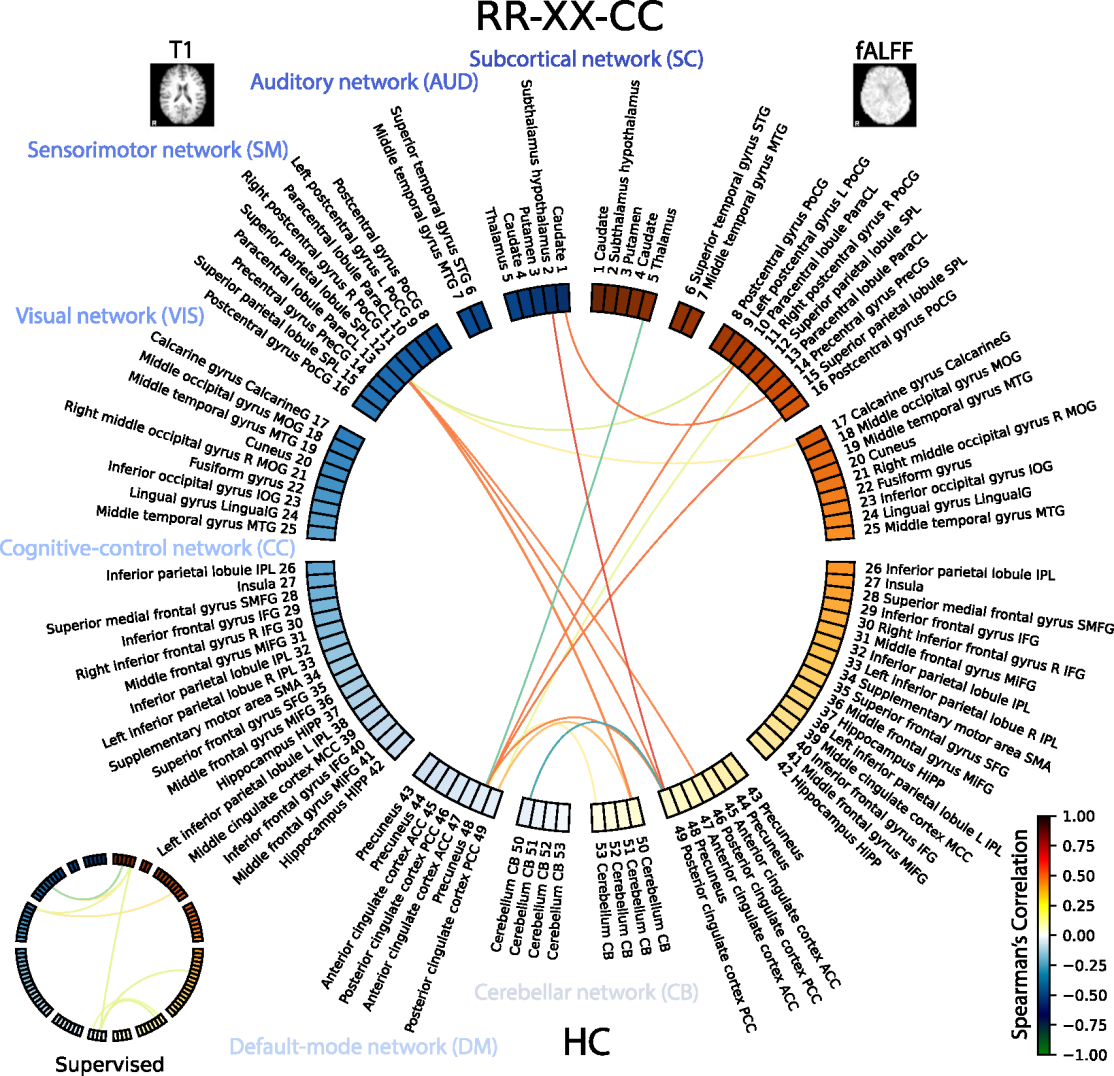
The Figure shows multimodal links for controls between T1 and fALFF of ROIs in the Neuromark atlas for the *RR-XX-CC* model, and for the *Supervised* model in the left bottom corner. The ROIs for T1 are shown on the left side with shades of blue, and the ROIs for fALFF are shown on the right side with shades of orange. The edge weights are defined by the correlation between dimensions in the representation vector between T1 and fALFF and colored according to the color bar. We show only significant edges based on a statistical z-test on Fisher z-transformed correlation coefficients (Hinkle et al., 1988). The *Supervised* models have very weak correlations (<0.39) compared to the multimodal *RR-XX-CC* model. The figure shows only the top 64 edges.

**Fig. 15.**
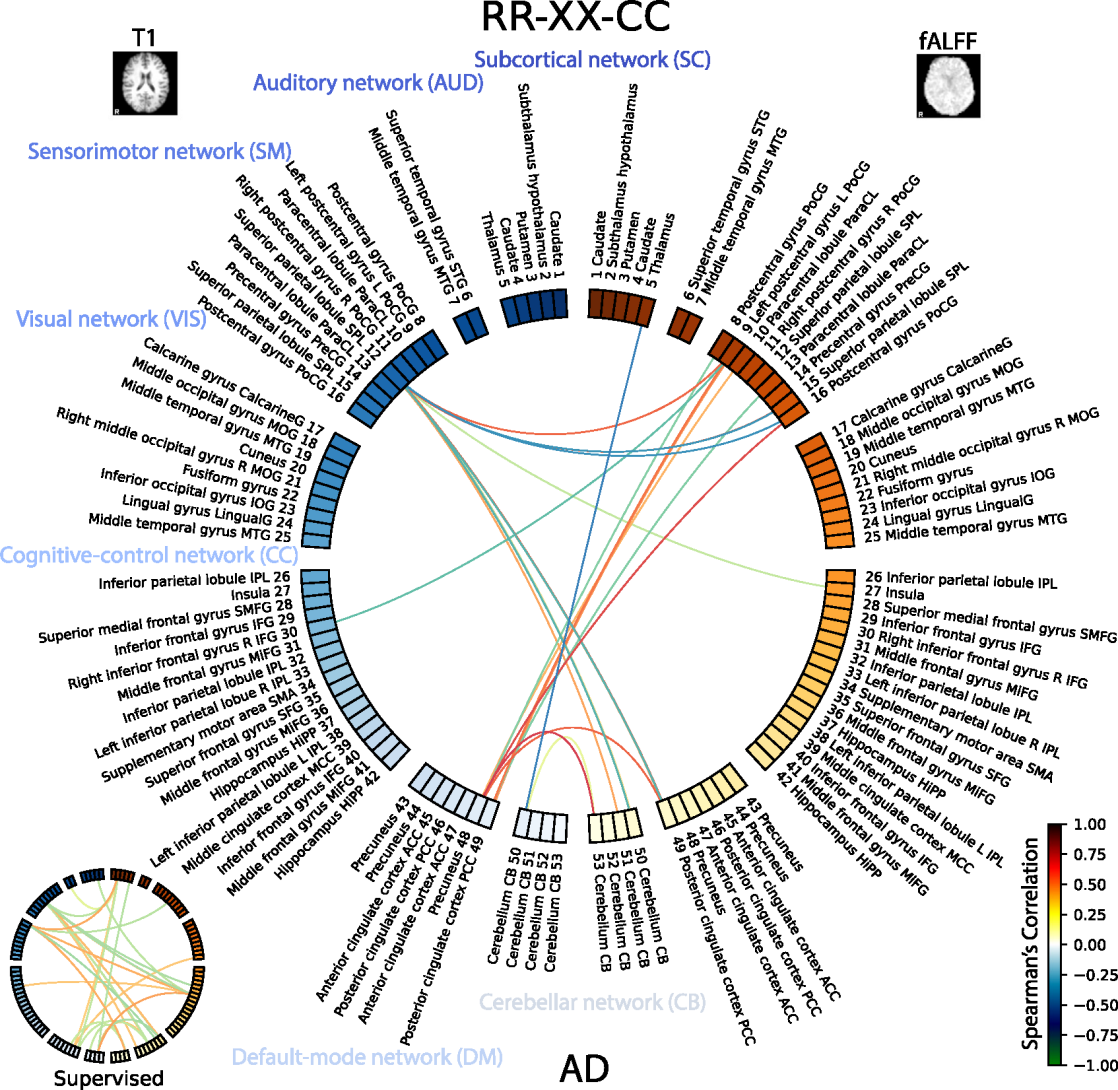
The Figure shows multimodal links for subjects with Alzheimer’s disease between T1 and fALFF of ROIs in the Neuromark atlas for the *RR-XX-CC* model, and for the *Supervised* model in the left bottom corner. The ROIs for T1 are shown on the left side with shades of blue, and the ROIs for fALFF are shown on the right side with shades of orange. The edge weights are defined by the correlation between dimensions in the representation vector between T1 and fALFF and colored according to the color bar. We show only significant edges based on a z-test from Fisher z-transformed correlation coefficients (Hinkle et al., 1988). The *Supervised* models have very weak correlations (<0.43) compared to the multimodal *RR-XX-CC* model. The figure shows only the top 64 edges.

The final summarization of the multimodal relationships is shown in Fig. 14 for healthy subjects and Fig. 15 for subjects with Alzheimer’s disease. Note, we show the top 64 edges with maximum by absolute value weights, and, specifically, we only focus on the self-supervised multimodal model *RR-XX-CC*. However, we also show the same diagram for the *Supervised* model in a restricted way. Because the correlation of the *Supervised* is much lower, and the model is learning representation unimodally, thus the relationships are more likely to be spurious. The Fig. 14 and Fig. 15 shows ROIs for T1 on the left side with blue hues and fALFF on the right side with red hues for HC and AD groups, respectively. Additionally, we group ROIs by functional networks defined in the Neuromark atlas.

The highest positive correlation was pinpointed by the *RR-XX-CC* model between the Subthalamus hypothalamus 2 (T1) and the Posterior cingulate cortex PCC 49 (fALFF) (Laxton et al., 2010), yielding a correlation coefficient of Spearman’s *r*(592) = 0.689*,p* < 0.05, as portrayed in Fig. 14 and Table 9. Conversely, the lowest correlation was discovered between the Posterior cingulate cortex PCC 49 (T1), and Cerebellum CB 50 (fALFF) (Zimny et al., 2011) with a Spearman correlation of *r*(592) = −0.686*,p* < 0.05. The other edges are described in Table 9.

In the detailed analysis in Fig. 15 and Table 10 on AD subjects for the *RR-XX-CC* model, the highest positive correlation is highlighted between Precuneus 48 (T1) and Cerebellum CB 53 (fALFF) (Parker et al., 2020), as depicted in Fig. 15. This association is quantified with a Spearman’s correlation coefficient of Spearman’s *r*(145) = 0.797*,p* < 0.05. On the other end, the most negative correlation was observed between Cerebellum CB 50 (T1) and Thalamus 5 (fALFF) (Martí-Juan et al., 2023), characterized by a Spearman’s *r*(145) = −0.822*,p* < 0.05. The other edges are described in Table 10.

These intricate neural associations brought to light by the *RR-XX-CC* model in the context of AD underscore the model’s capability in extracting meaningful relationships, offering a rich tapestry of insights for further neuroscientific exploration.

### 4.3. Hardware, reproducibility, and code

The experiments were performed using an NVIDIA V100. The code is implemented mainly using PyTorch (Paszke et al., 2019) and Catalyst (Kolesnikov, 2018) frameworks. The code is available at github. com/Entodi/fusion for reproducibility and further exploration by the scientific community.

## 5. Discussion

### 5.1. Multi-scale coordinated self-supervised models

Based on the classification performance and interpretability analysis, the proposed self-supervised multimodal multi-scale coordinated models can capture useful representation and multimodal relationships in the data. Compared to existing unimodal (*CR* and *AE*) and multimodal (*DCCAE*) counterparts, these models achieve higher discriminative performance in ROC-AUC on downstream tasks. While some of them can capture higher joint information content between modalities as measured by the CKA. Furthermore, these models can produce representations that, compared to the *Supervised* model, show competitive performance on T1 and outperform fALFF.

We show strong empirical evidence that the *XX* is the important relationship to encourage when high discriminative performance is the goal. This result is an evidence of the importance and existence of multi-scale local-to-global multimodal relationships in the functional and structural neuroimaging data, which other relationships cannot capture. However, in addition to *XX*, the *RR* objective is needed in the training objective to achieve higher alignment between models.

However, not all multimodal variants from the taxonomy of Fig. 2 result in robust and useful representations. Specifically, our experiments show that the *CC* relationship should not be used separately from other objectives, as the *CC* will optimize only the layers below the chosen one because the last layer will behave as a random projection. We show it only for a complete picture of achievable classification performance with all objectives in the taxonomy.

The CCA-based objectives did not show the good results expected in a multi-view scenario (Wang et al., 2015). However, our taxonomy revealed that *DCCA* is related to *SimCLR* (Chen et al., 2020), which led us to develop the *RR* model. While CCA maximizes correlations, *SimCLR* maximizes cosine similarity between representations of different modalities. However, the *SimCLR* objective has one more important difference: it performs an additional discrimination step on cosine similarity scores. Thus, it does two things: maximizes the similarity between modalities and simultaneously performs additional discrimination of pairs based on similarity. This task is more challenging because it needs to capture richer information to classify pairs of representations from different modalities based on similarity. In addition, the CCA-objective is prone to numerical instability (Wang et al., 2015). *RR* does not have such issues. We recommend using “softer” loss functions based on mutual information estimators with deep neural networks and not the “exact” solutions based on linear algebra in DCCAE (Wang et al., 2015).

While *AE* imposes additional computational complexity due to the decoder, it does not show increased classification accuracy over non-autoencoder methods. Specifically, the *AE* model struggles to deal with ternary classification tasks. Multimodal models from our proposed taxonomy have a reduced computational burden because they do not contain a volumetric decoder. These findings concur with the poor performance of autoencoders on datasets with natural images (Hjelm et al., 2019). To achieve greater performance, we hypothesize that autoencoders may require encoders and decoders of higher capacity. However, this will considerably increase the difficulty of training large volumetric models.

#### 5.1.1. Comparing self-supervised models on group discriminative regions

**Fig. 16.**
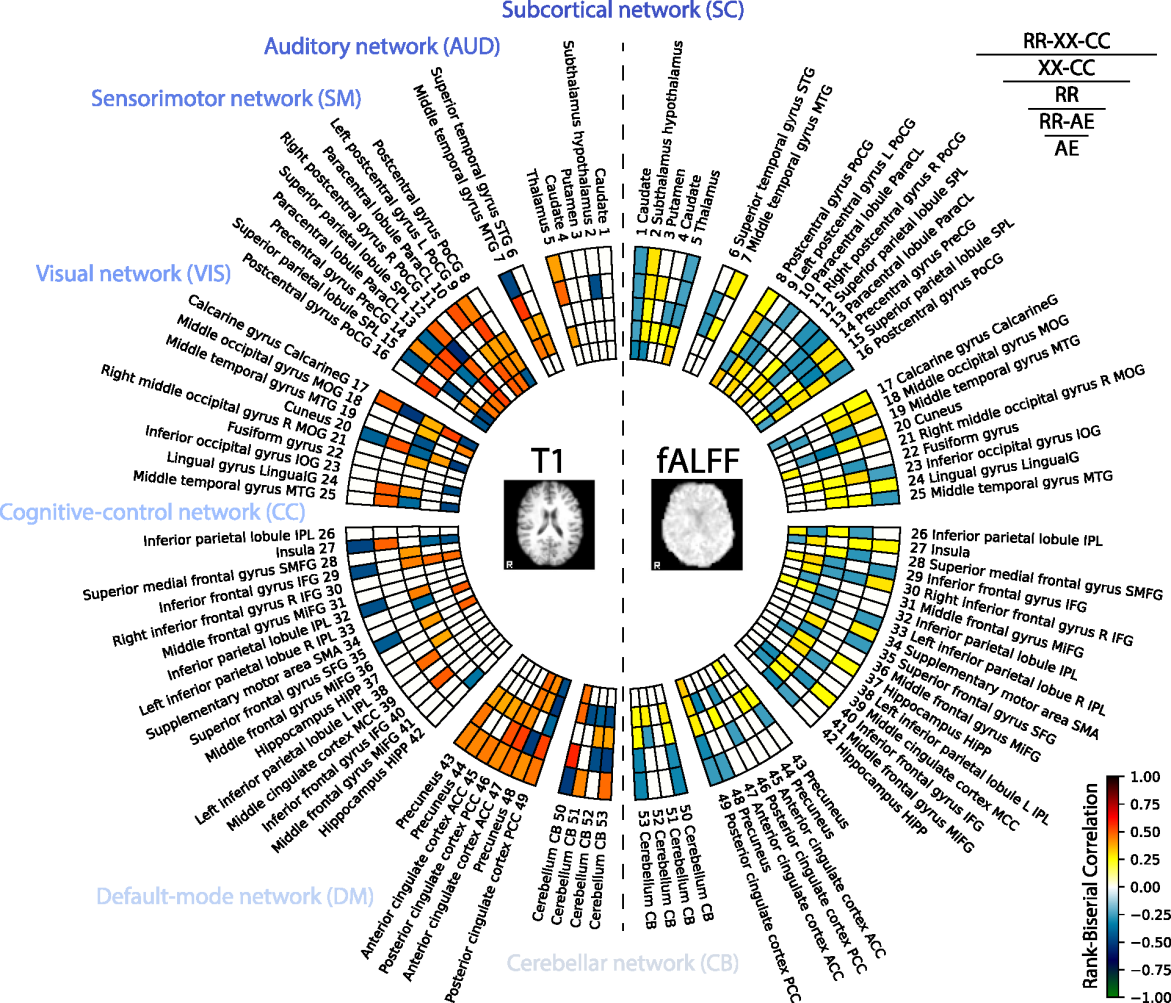
The figure shows the near absolute maximum (quantile 0.999) peak RBC values of the saliency for regions in the Neuromark atlas for the dimensions with the highest positive and negative importance (Betas in Logistic Regression) derived from the best fold on binary classification. The circles represent one of the self-supervised algorithms. The order from outer to inner is the following: *RR-XX-CC*, *XX-CC*, *RR*, *RR-AE*, and *AE*. The models are chosen based on significance in Tables 2 and 3. It can be seen that mostly self-supervised models agree on the regions. However, *AE* seems very different from the other four algorithms.

**Fig. F.21.**
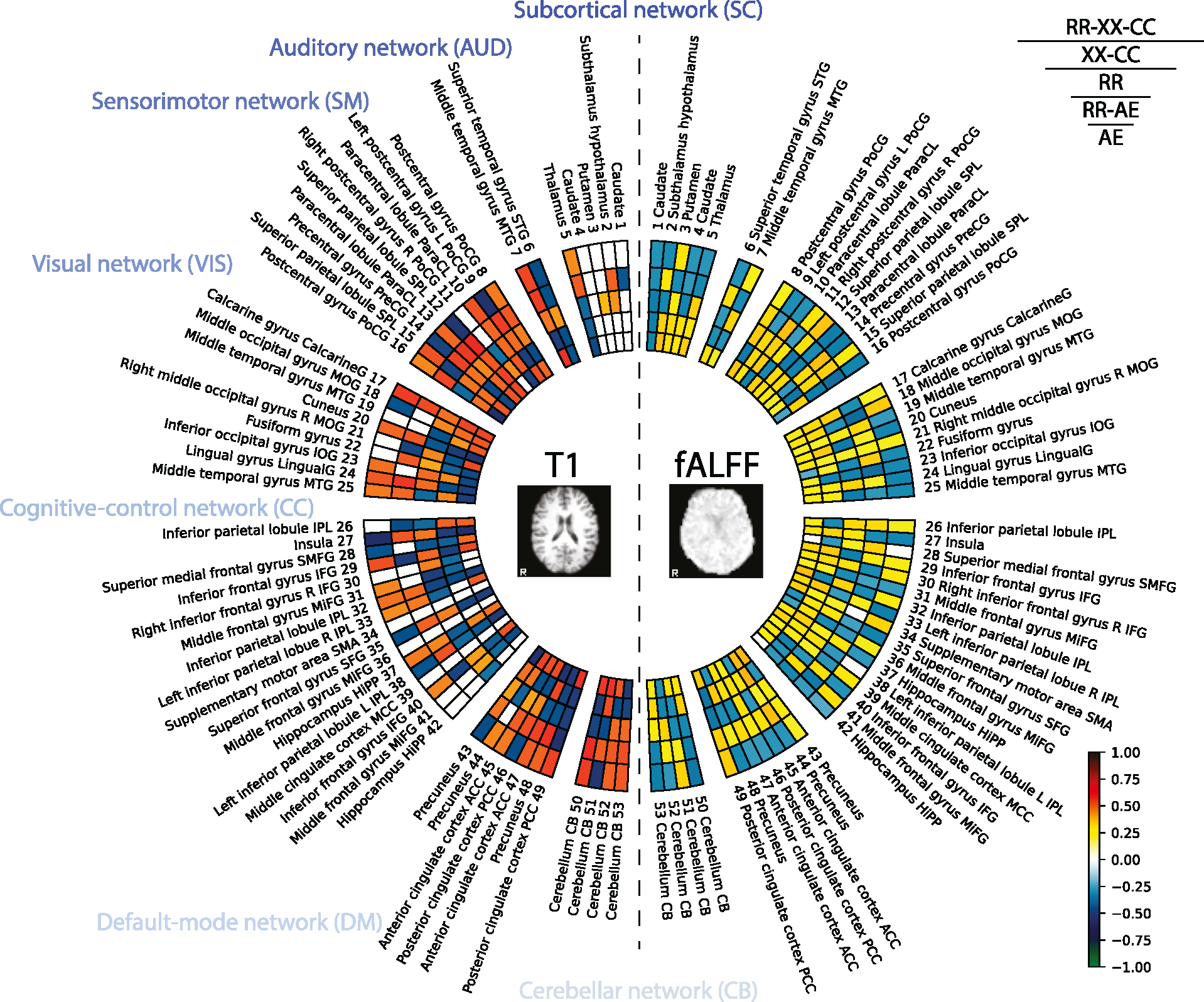
The figure shows the near absolute maximum (quantile 0.999) peak RBC values of the saliency for regions in the Neuromark atlas for the dimensions with non-zero importance (Betas in Logistic Regression) derived from the best fold on binary classification. The circles represent one of the self-supervised algorithms. The order from outer to inner is the following: *RR-XX-CC*, *XX-CC*, *RR*, *RR-AE*, and *AE*. The models are chosen based on significance in Tables 2 and 3. It can be seen that mostly self-supervised models agree on the regions. However, *AE* seems very different from the other four algorithms.

For a comprehensive analysis, we also ran group discriminative analyses for multimodal *XX-CC*, *RR*, *RR-AE*, and unimodal *AE*, given their competitive performance in Tables 2 and 3. The results are displayed in Fig. 16, which shows the dimensions with the highest positive and negative importance in Logistic Regression. To see analysis across all the dimensions with non-zero importance see Fig. F.21 in Appendix.

Based on Fig. 16, self-supervised algorithms share similarities in some regions. However, *RR-XX-CC* and *XX-CC* find similar regions, compared to other models. Especially, *AE* finds unique regions compared to multimodal models, while its combination with the *RR* objective, *RR-AE*, finds regions similar to *RR*.

This analysis in addition to its best performance on both tasks (see Table 2, Table 3, Table 4, and Table 5) further reinforces our choice to explore *RR-XX-CC*.

#### 5.1.2. Comparing models on the multimodal latent structure

In this part, we study the inductive biases of the models concerning the multimodal latent structure. The multimodal latent structure and CKA alignment have not been explored in the literature for multimodal models. Previously, multi-view methods such as DCCAE (Wang et al., 2015) have only computed captured correlation for multi-view image datasets, such as two-view MNIST.

**Fig. 17.**
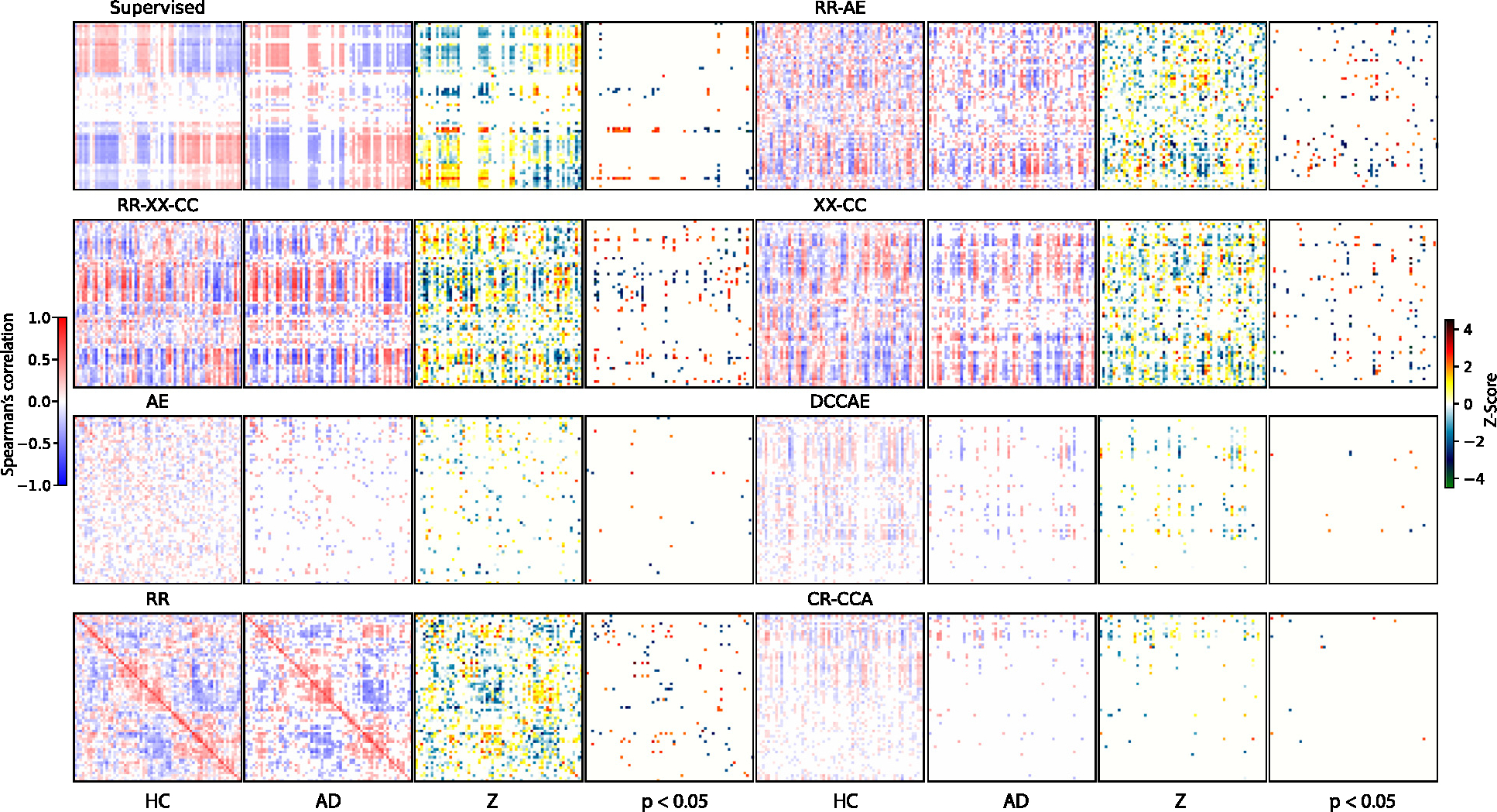
The multimodal latent structure of multiple models. The structure is computed by computing Spearman’s correlations between dimensions in the representation vector of T1 and fALFF across subjects separately for HC and AD. Each row and four consecutive columns correspond to a different model. The first column is a correlation for HC, the second — for AD, the third is a Z score from Z-test, and the fourth is the significant *p* < 0.05 correlation pairs based on Z-test. The correlation matrices of the HC subjects have been clustered using hierarchical (agglomerative) clustering using SciPy (Virtanen et al., 2020). Then the found linkage has been applied across correlations of the AD subjects.

We have computed cross-modal correlations between dimensions of the representation vector across subjects to compare learned multimodal latent structure. We computed these correlations separately for HC and AD subjects. The results are shown in Fig. 17.

Clearly, *AE*, *CR-CCA*, and *DCCAE* fail to capture the multimodal structure with the lowest correlations. We should not expect multimodal structure for unimodal *AE*, while for CCA-based multimodal models, we should expect it. The *Supervised* captures polarized structure.

The multimodal models *RR-XX-CC*, *XX-CC*, *RR*, and *RR-AE* capture much higher correlations and find more significant pairs of latent dimensions. Furthermore, higher correlation values agree with the higher CKA alignment measure shown in Fig. 8.

However, the multimodal structure differs in these models, which could explain the difference in the inductive biases. Specifically, *RR* has a diagonal structure. It might be the case that *RR* does not precisely capture the joint information we want to explore but assigns to each latent dimension in one modality a dimension from another as classification assigns a label to a representation. Hence it might be the reason why it fails to capture unique modality-specific information and not as strong as *RR-XX-CC*. The *RR-XX-CC* and *XX-CC* models share joint block structure behavior, which *XX* can explain as it estimates multimodal local–global relationships. The *RR-AE* shares some similarities with the block structure of the *RR-XX-CC* and *XX-CC* models. We hypothesize that it is primarily due to the autoencoder aspect of the *RR-AE* as it requires capturing local structure for reconstruction.

Notably, the proposed multimodal models *RR-XX-CC*, *XX-CC*, *RR*, and *RR-AE* find many highly correlated group-discriminative pairs compared to unimodal (*Supervised* and *AE*), and CCA-based multimodal models. We summarized the number of multimodal links and max positive and negative correlations in Table 11. We count only significant *p* < 0.05 multimodal links based on the Z test. The multimodal models *RRXX-CC* and *XX-CC* that use mutual information estimator show higher number of significant links and a higher positive and negative correlations. These result reinforce the benefit of multimodal coordination for interpretability due to the ability to explore the multimodal latent structure by having separate representation vectors.

The *RR-XX-CC* find the highest number of significant multimodal links 252.00 ± 15.72. The closest is *XX-CC* with 209.60 ± 23.33. Then *RR* and *RR-AE* with 121.40 ± 8.16 and 136.80 ± 11.41, respectively. While *Supervised* (62.40 ± 18.08) finds more than unimodal *AE* (22.80 ± 2.13) or CCA-based DCCAE (18.00 ± 2.59) and CR-CCA (17.40 ± 3.33). Based on the correlations, the *RR-XX-CC*, *XX-CC*, *RR*, and *RR-AE* achieve correlations above 0.70, while other four models achieve maximum 0.57. This evidence further supports the benefit of mutual information estimator and multimodal training.

### 5.2. Future work

The models we have constructed in this work do not disentangle representation into joint and unique modality-specific representations. The analysis between CKA and downstream performance shows the existence of a joint subspace between modalities, and a specific amount of joint information measured by CKA is important to learn representations valuable for downstream tasks and interpretability. Future work could consider models that explicitly represent factors of the joint and unique components. Some related ideas have been explored in work on natural images when disentangling content and style (Von Kügelgen et al., 2021; Lyu et al., 2021), similarly, for neural data with variational autoencoders (Liu et al., 2021).

In the current work, we have not considered the evaluation of the models on other MRI datasets that would introduce covariate shifts due to different scanning protocols or scanners. The standard strategy is to perform transfer learning or domain adaptation. However, fine-tuning to a different objective can lead to catastrophic forgetting (Kirkpatrick et al., 2017) and destroy the properties of the representations that we focus on achieving. That is one of the reasons why we use linear evaluation using features from a frozen encoder. Fine-tuning on the same dataset with a supervised objective can lead to losing the learned properties. Furthermore, the maximization of mutual information does not solve the covariate shift problem by default. Hence additional engineering (Ruan et al., 2021) would be required. Future methods should be able to deal with such covariate shifts.

Our analysis does not consider the family of multimodal generative variational models (Kingma and Welling, 2013). Volumetric variational models are computationally expensive, and the field is under active development. Including all possible models with all possible underlying technology was not precisely our goal and would make the already extensive list of models even harder to analyze. Future work may consider variational models under the same taxonomy for a fair comparison and detailed analysis of multimodal fusion applications.

There is more that can be done concerning the explainability of the models. Currently, a common choice to model neuroimaging data is to use a convolutional neural network (CNN) (Abrol et al., 2021). However, the simple application of CNNs leads to representation, where each dimension captures multiple ROIs. This effect creates difficulties in analyzing the cross-modal relationships between modalities. The multimodal links between ROIs can only be measured by the correlation between dimensions of a representation in different modalities. Thus the measured links do not represent the multimodal link between one ROI and another ROI but rather between dimensions. Future work may consider ROI-based representations.

In addition, as we want to focus on unsupervised models without using group labels, we used HC and AD groups to show group differences or identify ROIs in our analysis. However, the data may contain phenotypically small patient groups not represented by HC or AD groups. Doing group analysis in such a scenario will be hard because we do not have the labels. Thus future work can consider additional clustering of the representation for finding such subgroups that explainability methods can further analyze.

## 6. Conclusions

In this work, we presented a novel multi-scale coordinated framework for representation learning from multimodal neuroimaging data. We showed that self-supervised approaches can learn meaningful and useful representations that capture regions of interest with group differences without accessing group labels during pre-training. We developed evaluation methodologies to access the properties of representations learned by models within the family of models in downstream task analysis, measurements of joint subspace, and explainability evaluations.

One of our models, *RR-XX-CC* consistently outperformed previous unsupervised unimodal *AE* and multimodal *DCCAE* (Wang et al., 2015), *RR* (SimCLR Chen et al., 2020), *XX-CC* (AMDIM Bachman et al., 2019) models, achieved near-supervised performance (based on significance test) on both classification tasks, improved data-efficiency and outperformed *Supervised* model on fALFF. Further, our findings suggest the importance of multi-scale local-to-global multimodal relationships *XX* that considerably improve the performance over previous methods and within the proposed family of models. This result suggests that multi-scale relationships exist between local structure and global summary of the inputs in different modalities previously neglected in multimodal representation learning. While we also show that the objective *RR* is important to capture the joint information. In contrast, such common approach as CCA could not achieve high alignment between modalities according to the CKA measure.

The *RR-XX-CC* model, selected based on the best classification performance and higher joint information content via CKA, was able to capture important regions of interest related to Alzheimer’s disease such as the cuneus (Wu et al., 2021), subthalamus hypothalamus (Zhu et al., 2019; Ríos et al., 2022), thalamus (Coupé et al., 2019), insula (Philippi et al., 2020), hippocampus (Yang et al., 2022). Notably, the *RR-XX-CC* model can capture higher number of group-discriminative significant multimodal links that are supported by the literature such as subthalamus hypothalamus and posterior cingulate cortex (Laxton et al., 2010), posterior cingulate cortex and cerebellum (Zimny et al., 2011), precuneus and cerebellum (Parker et al., 2020), cerebellum CB 50 and thalamus 5 (Martí-Juan et al., 2023). Notably, the model did not have access to the labels during pre-training. Hence, such a model can be helpful for exploratory scientific data analyses.

The showcased benefits of applying a comprehensive approach, evaluating a taxonomy of methods, and performing extensive qualitative and quantitative evaluation suggest that the nascent multimodal representation learning is a field with significant potential in neuroimaging. Our work lays a foundation for future robust and increasingly more interpretable multimodal models.

## Figures and Tables

**Table 1:** The details of the dataset splits and usage.

Dataset: 826 subjects			

Splits	Stratified 5-fold cross-validation: 726 subjects		Hold-out: 100 subjects
		
Set	Training	Validation	Test

Number of samples	580–582 subjects (2828–2944 pairs)	144–146 subjects (653–769 pairs)	100 subjects (424 pairs)

**Table 2:** Mean and standard error of ROCAUC and CKA metrics for binary classification task on the T1 modality. The significance is computed with the Wilcoxon signed-rank test and additional Holm correction for multiple comparisons.

Model	Baseline	Multimodal	ROCAUC	CKA	Significance

**Supervised**	✓		**86.1 ± 1.0**	21.4 ± 1.4	N/A
**AE**	✓		**85.9 ± 0.3**	50.8 ± 0.4	
**RR-XX-CC**		✓	**84.0 ± 0.6**	76.6 ± 0.4	
**RR-AE**		✓	**84.0 ± 0.6**	68.2 ± 0.9	

CR-XX-CC		✓	83.2 ± 1.1	54.4 ± 1.4	.
CR-XX		✓	82.5 ± 1.3	52.7 ± 1.3	*
CR-CCA		✓	82.5 ± 0.9	21.7 ± 0.6	.
CR-CC		✓	81.8 ± 1.2	15.1 ± 0.6	.
RR-CR-XX		✓	81.3 ± 1.6	39.4 ± 1.1	.
XX		✓	80.5 ±2.4	65.2 ± 1.3	.
XX-CC	✓	✓	80.3 ± 1.6	67.4 ± 2.1	*
RR	✓	✓	80.1 ± 1.3	72.3 ± 0.9	*
RR-XX		✓	79.7 ± 1.0	73.1 ± 2.5	*
DCCAE	✓	✓	79.6 ± 1.7	21.2 ± 0.6	*
RR-CR		✓	79.4 ± 1.3	29.7 ± 0.4	*
RR-CC		✓	79.0 ± 1.5	72.8 ± 1.2	*
RR-CR-CC		✓	78.9 ± 1.2	27.3 ± 0.5	*
CR	✓		78.8 ± 1.9	15.1 ± 0.6	*
RR-CR-XX-CC		✓	78.2 ± 1.3	39.7 ± 1.6	*
CC		✓	77.3 ± 2.1	47.9 ± 1.6	*

The significance is denoted as follows:

*** *p* < 0.001

** *p* < 0.01

* *p* < 0.05

*p* < 0.1. Bold names are the best models based on ROCAUC and significance testing.

**Table 3:** Mean and standard error of ROCAUC and CKA metrics as mean ± SE for binary classification task on fALFF on a hold-out test set. The significance is computed with the Wilcoxon signed-rank test and additional Holm correction for multiple comparisons.

Model	Baseline	Multimodal	ROCAUC	CKA	Significance

**RR-XX-CC**		✓	**79.4 ± 1.3**	76.6 ± 0.4	N/A
**XX-CC**	✓	✓	**77.3 ± 1.4**	67.4 ± 2.1	
**RR**	✓	✓	**75.9 ± 1.8**	72.3 ± 0.9	

RR-CR-XX		✓	75.6 ± 1.1	39.4 ± 1.1	*
CR-XX-CC		✓	75.0 ± 0.6	54.4 ± 1.4	.
XX		✓	74.8 ± 1.0	65.2 ± 1.3	.
RR-CR-XX-CC		✓	74.6 ± 0.8	39.7 ± 1.6	*
RR-CC		✓	74.6 ± 1.8	72.8 ± 1.2	.
Supervised	✓		74.2 ± 1.1	21.4 ± 1.4	*
CR-XX		✓	73.1 ± 1.9	52.7 ± 1.3	.
RR-AE		✓	73.0 ± 1.8	68.2 ± 0.9	*
RR-XX		✓	72.7 ± 1.2	73.1 ± 2.5	*
RR-CR-CC		✓	72.5 ± 0.8	27.3 ± 0.5	*
RR-CR		✓	72.2 ± 0.8	29.7 ± 0.4	*
CR-CC		✓	71.8 ± 1.9	15.1 ± 0.6	.
DCCAE	✓	✓	71.5 ± 1.4	21.2 ± 0.6	*
CR-CCA		✓	71.1 ± 1.6	21.7 ± 0.6	*
AE	✓		70.9 ± 2.3	50.8 ± 0.4	*
CR	✓		68.6 ± 1.0	15.1 ± 0.6	*
CC		✓	65.5 ± 3.2	47.9 ± 1.6	*

The significance is denoted as follows:

*** *p* < 0.001

** *p* < 0.01

* *p* < 0.05

*p* < 0.1. Bold names are the best models based on ROCAUC and significance testing.

**Table 4:** Mean and standard error of OVO ROCAUC Macro and CKA metrics for ternary classification task for T1 on the hold-out test set. The significance is computed with the Wilcoxon signed-rank test and additional Holm correction for multiple comparisons.

Model	Baseline	Multimodal	OVO ROCAUC Macro	CKA	Significance

**Supervised**	✓		**73.9 ± 1.3**	21.9 ± 1.6	N/A
**RR-XX-CC**		✓	**70.2 ± 1.2**	73.9 ± 1.1	.

RR-CR-XX-CC		✓	68.6 ± 0.7	38.0 ± 1.3	*
RR-XX		✓	68.3 ± 1.3	73.0 ± 0.8	.
CR-XX-CC		✓	68.2 ± 1.1	39.8 ± 0.4	*
RR-CR-XX		✓	68.0 ± 1.5	37.0 ± 1.4	.
RR-CR-CC		✓	67.3 ± 0.8	30.3 ± 0.8	*
AE	✓		67.1 ± 1.5	48.6 ± 0.3	*
RR-CC		✓	67.1 ± 1.6	70.5 ± 0.7	.
CR	✓		66.8 ± 1.4	15.2 ± 0.2	*
CR-XX		✓	66.8 ± 1.4	30.5 ± 1.7	.
DCCAE	✓	✓	66.6 ± 1.4	15.0 ± 1.0	.
XX		✓	66.5 ± 1.5	65.2 ± 0.9	*
XX-CC	✓	✓	66.5 ± 0.9	66.2 ± 1.8	*
CR-CCA		✓	65.3 ± 2.2	21.5 ± 0.4	*
RR-AE		✓	64.4 ± 1.3	70.9 ± 1.1	*
RR-CR		✓	64.3 ± 1.8	27.9 ± 0.5	*
CR-CC		✓	63.8 ± 2.1	14.9 ± 0.7	*
CC		✓	63.7 ± 1.1	45.7 ± 1.3	*
RR	✓	✓	63.7 ± 1.3	69.5 ± 0.6	*

The significance is denoted as follows:

*** *p* < 0.001

** *p* < 0.01

* *p* < 0.05

*p* < 0.1. Bold names are the best models based on ROCAUC and significance testing.

**Table 5:** Mean and standard error of OVO ROCAUC Macro and CKA metrics for ternary classification task for fALFF on a hold-out test set. The significance is computed with the Wilcoxon signed-rank test and additional Holm correction for multiple comparisons.

Model	Baseline	Multimodal	OVO ROCAUC Macro	CKA	Significance

**RR-XX**		✓	**63.2 ± 2.2**	73.0 ± 0.8	N/A
**Supervised**	✓		**62.9 ± 3.2**	21.9 ± 1.6	
**XX**		✓	**62.5 ± 1.2**	65.2 ± 0.9	
**RR-XX-CC**		✓	**62.2 ± 1.6**	73.9 ± 1.1	

XX-CC	✓	✓	60.7 ± 1.9	66.2 ± 1.8	
RR-CR-CC		✓	59.2 ± 2.8	30.3 ± 0.8	
CR-XX-CC		✓	59.2 ± 1.0	39.8 ± 0.4	
CR-CCA		✓	59.0 ± 2.2	21.5 ± 0.4	*
DCCAE	✓	✓	59.0 ± 0.7	15.0 ± 1.0	
RR-CR-XX		✓	58.5 ± 0.6	37.0 ± 1.4	
CR-CC		✓	58.3 ± 1.7	14.9 ± 0.7	*
CR-XX		✓	58.1 ± 1.0	30.5 ± 1.7	.
RR-CC		✓	57.6 ± 1.7	70.5 ± 0.7	.
RR-CR-XX-CC		✓	57.4 ± 3.0	38.0 ± 1.3	
RR-AE		✓	57.3 ± 1.9	70.9 ± 1.1	
CR	✓		55.2 ± 2.3	15.2 ± 0.2	.
RR	✓	✓	55.0 ± 1.6	69.5 ± 0.6	.
AE	✓		53.9 ± 1.3	48.6 ± 0.3	*
RR-CR		✓	53.7 ± 1.8	27.9 ± 0.5	*
CC		✓	51.9 ± 1.0	45.7 ± 1.3	*

The significance is denoted as follows:

*** *p* < 0.001

** *p* < 0.01

* *p* < 0.05

*p* < 0.1. Bold names are the best models based on ROCAUC and significance testing.

**Table 6:** Label-efficiency on binary classification task on hold-out test for T1 measured as ROCAUC and reported as mean ± standard error.

Model	Baseline	Multimodal	Set	10%	25%	50%	75%	100%

Supervised	✓		**African American**	75.7 ± 4.04	**81.4 ± 1.26**	**85.0 ± 0.79**	**84.6 ± 0.72**	**85.2 ± 0.34**
			Non-Hispanic Caucasian	72.2 ± 3.46	81.4 ± 2.05	83.7 ± 1.75	84.6 ± 1.58	85.3 ± 1.22

AE	✓		**African American**	76.8 ± 5.93	77.9 ± 2.68	80.9 ± 1.79	79.4 ± 2.27	82.8 ± 1.01
			Non-Hispanic Caucasian	78.1 ± 3.09	80.4 ± 1.05	84.4 ± 0.73	84.2 ± 1.15 *	85.6 ± 0.53

DCCAE (Wang et al., 2015)	✓	✓	**African American**	74.0 ± 6.05	79.4 ± 2.87	81.2 ± 1.10	84.0 ± 0.36	83.1 ± 0.79
			Non-Hispanic Caucasian	74.9 ± 5.60	77.7 ± 0.96	79.1 ± 1.30	81.5 ± 0.98	81.1 ± 0.96

RR (Tian et al., 2019; Chen et al., 2020)	✓	✓	**African American**	70.9 ± 2.55	78.9 ± 1.13	78.4 ± 1.26	80.7 ± 1.27	80.6 ± 2.15
			Non-Hispanic Caucasian	75.9 ± 1.32	78.2 ± 1.40	76.7 ± 1.49	79.9 ± 0.82	80.8 ± 1.35

XX-CC (Bachman et al., 2019)	✓	✓	**African American**	**82.4 ± 2.01**	79.4 ± 0.74	82.5 ± 2.10	83.7 ± 1.54	**84.3 ± 1.14**
			Non-Hispanic Caucasian	77.2 ± 2.32	76.3 ± 4.62	79.9 ± 2.32	79.7 ± 2.17	80.5 ± 1.75

RR-XX-CC		✓	**African American**	**81.4 ± 2.37**	**83.4 ± 1.47**	**82.6 ± 2.34**	**84.2 ± 1.70**	83.9 ± 1.66
			Non-Hispanic Caucasian	78.7 ± 2.27	83.9 ± 1.32	81.8 ± 2.06	83.6 ± 1.55	84.4 ± 0.60

Bold text is for the model with the best performance on African American subjects, and non-bold is the best performance on Non-Hispanic Caucasians. The blue color is the best model, and the orange color is the second best model. The start * marks significant (*p* < 0.05) differences in the performance based on Wilcoxon signed-rank test with the alternative hypothesis that “the performance of Non-Hispanic Caucasian subjects is greater than that of African American subjects”.

**Table 7:** Label-efficiency on binary classification transfer task for fALFF measured as ROCAUC and reported as mean ± standard error.

Model	Baseline	Multimodal	Set	10%	25%	50%	75%	100%

Supervised	✓		**African American**	60.0 ± 2.71	63.4 ± 2.34	68.4 ± 1.32	**70.4 ± 0.85**	**71.5 ± 0.59**
			Non-Hispanic Caucasian	62.8 ± 4.03	68.2 ± 2.25	69.6 ± 2.20	72.8 ± 1.54	73.4 ± 0.86

AE	✓		**African American**	58.3 ± 1.56	54.5 ± 2.38	62.4 ± 0.97	64.2 ± 2.83	64.5 ± 1.35
			Non-Hispanic Caucasian	61.9 ± 4.90	67.9 ± 2.82 *	74.7 ± 2.41 *	68.3 ± 4.00 *	70.6 ± 2.53 *

DCCAE (Wang et al., 2015)	✓	✓	**African American**	58.5 ± 2.22	59.7 ± 2.07	63.3 ± 1.33	63.6 ± 0.98	66.0 ± 2.39
			Non-Hispanic Caucasian	66.7 ± 4.11	64.4 ± 2.88	70.3 ± 1.27 *	70.6 ± 1.65 *	71.6 ± 1.76 *

RR (Tian et al., 2019; Chen et al., 2020)	✓	✓	**African American**	60.3 ± 4.43	63.3 ± 5.76	**69.5 ± 2.59**	**71.1 ± 1.44**	**72.0 ± 2.17**
			Non-Hispanic Caucasian	64.8 ± 3.28	72.3 ± 3.38	74.2 ± 1.57	71.9 ± 1.20	74.3 ± 1.59

XX-CC (Bachman et al., 2019)	✓	✓	**African American**	**64.4 ± 2.48**	**66.8 ± 1.24**	68.6 ± 2.35	69.3 ± 1.36	70.3 ± 1.93
			Non-Hispanic Caucasian	75.7 ± 2.98 *	78.1 ± 2.76 *	78.3 ± 2.16	77.5 ± 2.34	78.1 ± 1.02 *

RR-XX-CC		✓	**African American**	**66.0 ± 2.27**	**69.1 ± 2.53**	**69.8 ± 2.64**	69.0 ± 2.32	71.2 ± 2.75
			Non-Hispanic Caucasian	74.4 ± 4.28	78.1 ± 0.78 *	77.6 ± 1.14 *	77.9 ± 1.23	78.3 ± 1.23

Bold text is for the model with the best performance on African American subjects, and non-bold is the best performance on Non-Hispanic Caucasians. The blue color is the best model, and the orange color is the second best model. The start * marks significant (*p* < 0.05) differences in the performance based on Wilcoxon signed-rank test with the alternative hypothesis that “the performance of Non-Hispanic Caucasian subjects is greater than that of African American subjects”.

**Table 8:** The table for the Fig. 12 provides detailed quantitative information on the near absolute maximum (quantile 0.999) Peak RBC values of the saliency for regions in the Neuromark atlas for the dimensions with highest and lowest importance (Betas in Logistic Regression), derived from the best fold on binary classification.

Model	Region	Supervised		RR-XX-CC		Literature
			
Modality		T1	fALFF	T1	fALFF	

0	1 Caudate	0.608	–	–	−0.262	Persson et al. (2018), Stein et al. (2011), Coupé et al. (2019)
1	2 Subthalamus hypothalamus	−0.564	**0.346**	–	0.294	Zhu et al. (2019), Rios et al. (2022)
2	3 Putamen	**−0.607**	−0.407	–	–	Coupé et al. (2019)
3	5 Thalamus	0.566	–	0.398	−0.248	Coupé et al. (2019)
4	7 Middle temporal gyrus MTG	0.552	–	−0.484	0.269	Berron et al. (2020)
5	8 Postcentral gyrus PoCG	–	–	–	0.252	Zhang et al. (2020)
6	9 Left postcentral gyrus L PoCG	–	–	0.442	–	Zhang et al. (2020)
7	10 Paracentral lobule ParaCL	–	–	**0.511**	−0.245	Jang et al. (2017)
8	12 Superior parietal lobule SPL	0.569	–	0.489	−0.283	Prawiroharjo et al. (2020), Corriveau-Lecavalier et al. (2020)
9	13 Paracentral lobule ParaCL	–	–	−0.397	−0.303	Jang et al. (2017)
10	14 Precentral gyrus PreCG	−0.604	–	0.425	0.290	Behfar et al. (2020)
11	15 Superior parietal lobule SPL	–	–	−0.422	0.283	Prawiroharjo et al. (2020), Corriveau-Lecavalier et al. (2020)
12	16 Postcentral gyrus PoCG	–	–	–	−0.272	Zhang et al. (2020)
13	17 Calcarine gyrus CalcarineG	–	–	0.489	0.244	Wingo et al. (2020)
14	18 Middle occipital gyrus MOG	–	–	–	0.299	Frisoni et al. (2022)
15	20 Cuneus	–	–	−0.410	**0.308**	Wu et al. (2021)
16	23 Inferior occipital gyrus IOG	–	–	–	−0.242	Feng et al. (2019a)
17	24 Lingual gyrus LingualG	–	–	–	0.249	Liu et al. (2017)
18	25 Middle temporal gyrus MTG	−0.551	–	–	−0.267	Berron et al. (2020)
19	27 Insula	−0.603	–	−0.488	0.262	Philippi et al. (2020)
20	29 Inferior frontal gyrus IFG	–	–	−0.466	−0.272	Hallam et al. (2020), Chen et al. (2023)
21	30 Right inferior frontal gyrus R IFG	**0.619**	−0.414	–	0.289	Chen et al. (2023), Hallam et al. (2020)
22	31 Middle frontal gyrus MiFG	–	**−0.415**	–	–	Chen et al. (2023)
23	32 Inferior parietal lobule IPL	0.582	−0.414	−0.455	–	Greene et al. (2010), Zhou et al. (2015)
24	33 Left inferior parietal lobule R IPL	0.602	–	–	−0.262	Greene et al. (2010), Zhou et al. (2015)
25	34 Supplementary motor area SMA	–	–	–	0.278	Ye et al. (2019)
26	35 Superior frontal gyrus SFG	–	–	−0.426	−0.264	Hirono et al. (1998)
27	36 Middle frontal gyrus MiFG	−0.568	−0.410	–	–	Cheung et al. (2021)
28	37 Hippocampus HiPP	–	**−0.415**	–	0.247	Yang et al. (2022)
29	40 Inferior frontal gyrus IFG	–	–	–	0.254	Hallam et al. (2020), Chen et al. (2023)
30	43 Precuneus	–	−0.411	0.431	–	Casula et al. (2023), Guennewig et al. (2021)
31	44 Precuneus	–	0.344	0.436	–	Casula et al. (2023), Guennewig et al. (2021)
32	45 Anterior cingulate cortex ACC	−0.593	–	0.451	–	Tekin et al. (2001)
33	46 Posterior cingulate cortex PCC	–	−0.411	0.427	–	Minoshima et al. (1994), Rahim et al. (2023)
34	47 Anterior cingulate cortex ACC	–	–	0.459	–	Tekin et al. (2001)
35	48 Precuneus	–	–	0.421	−0.255	Casula et al. (2023), Guennewig et al. (2021)
36	49 Posterior cingulate cortex PCC	−0.556	–	0.460	−0.258	Minoshima et al. (1994), Rahim et al. (2023)
37	50 Cerebellum CB	−0.551	–	**−0.508**	**−0.316**	Kolbeinsson et al. (2020)
38	51 Cerebellum CB	–	–	0.433	–	Kolbeinsson et al. (2020)
39	53 Cerebellum CB	–	–	0.470	−0.315	Kolbeinsson et al. (2020)

**Table 9:** The table for the Fig. 14 provides detailed quantitative information on the multimodal links for healthy controls between T1 and fALFF of ROIs in the Neuromark atlas for *RR-XX-CC* model.

	Correlation	ROI 1	ROI 2	Literature

0	0.509	Superior parietal lobule SPL 12	50 Cerebellum CB	Weiler et al. (2015)
1	0.562	Caudate 1	14 Precentral gyrus PreCG	Liang et al. (2021)
2	−0.396	Superior parietal lobule SPL 12	50 Cerebellum CB	Weiler et al. (2015)
3	0.480	Superior parietal lobule SPL 12	50 Cerebellum CB	Weiler et al. (2015)
4	0.540	Superior parietal lobule SPL 12	49 Posterior cingulate cortex PCC	Gaubert et al. (2021)
5	0.553	Precuneus 48	16 Postcentral gyrus PoCG	Tosun et al. (2014)
6	**0.689**	Subthalamus hypothalamus 2	49 Posterior cingulate cortex PCC	Laxton et al. (2010)
7	0.539	Precuneus 48	10 Paracentral lobule ParaCL	Zhang et al. (2022)
8	−0.544	Precuneus 48	5 Thalamus	Ryu et al. (2010)
9	−0.224	10 Paracentral lobule ParaCL	Superior parietal lobule SPL 12	Ekblad et al. (2020)
10	**−0.686**	49 Posterior cingulate cortex PCC	Cerebellum CB 50	Zimny et al. (2011)
11	0.103	17 Calcarine gyrus CalcarineG	Superior parietal lobule SPL 12	Hu et al. (2019b)
12	0.509	46 Posterior cingulate cortex PCC	Superior parietal lobule SPL 12	Gaubert et al. (2021)
13	0.513	49 Posterior cingulate cortex PCC	Precuneus 48	Walhovd et al. (2010)
14	0.328	50 Cerebellum CB	Posterior cingulate cortex PCC 49	Zimny et al. (2011)
15	0.092	53 Cerebellum CB	Precuneus 48	Parker et al. (2020)
16	−0.165	12 Superior parietal lobule SPL	Posterior cingulate cortex PCC 49	Gaubert et al. (2021)

**Table 10:** The table for the Fig. 15 provides detailed quantitative information on the multimodal links for AD between T1 and fALFF of ROIs in the Neuromark atlas for *RR-XX-CC* model.

	Correlation	ROI 1	ROI 2	Literature

0	−0.616	Right inferior frontal gyrus R IFG 30	9 Left postcentral gyrus L PoCG	Xie et al. (2020)
1	0.635	Superior parietal lobule SPL 12	49 Posterior cingulate cortex PCC	Gaubert et al. (2021)
2	0.628	Superior parietal lobule SPL 12	9 Left postcentral gyrus L PoCG	Sintini et al. (2019)
3	**−0.822**	Cerebellum CB 50	5 Thalamus	Martí-Juan et al. (2023)
4	−0.754	Superior parietal lobule SPL 12	15 Superior parietal lobule SPL	Gu and Zhang (2019)
5	−0.773	Superior parietal lobule SPL 12	16 Postcentral gyrus PoCG	Sintini et al. (2019)
6	−0.508	Precuneus 48	8 Postcentral gyrus PoCG	Tosun et al. (2014)
7	0.735	Precuneus 48	16 Postcentral gyrus PoCG	Tosun et al. (2014)
8	0.576	Posterior cingulate cortex PCC 49	9 Left postcentral gyrus L PoCG	Guerrier et al. (2018)
9	−0.653	Superior parietal lobule SPL 12	50 Cerebellum CB	Weiler et al. (2015)
10	0.441	Superior parietal lobule SPL 12	49 Posterior cingulate cortex PCC	Gaubert et al. (2021)
11	0.376	Precuneus 48	10 Paracentral lobule ParaCL	Zhang et al. (2022)
12	0.491	Posterior cingulate cortex PCC 49	9 Left postcentral gyrus L PoCG	Guerrier et al. (2018)
13	−0.518	Posterior cingulate cortex PCC 49	13 Paracentral lobule ParaCL	Bi et al. (2021)
14	0.632	Superior parietal lobule SPL 12	15 Superior parietal lobule SPL	Walhovd et al. (2009)
15	**0.797**	Precuneus 48	53 Cerebellum CB	Parker et al. (2020)
16	−0.314	27 Insula	Superior parietal lobule SPL 12	Chételat et al. (2017)
17	−0.344	50 Cerebellum CB	Superior parietal lobule SPL 12	Weiler et al. (2015)
18	0.638	49 Posterior cingulate cortex PCC	Precuneus 48	Walhovd et al. (2010)
19	−0.653	49 Posterior cingulate cortex PCC	Superior parietal lobule SPL 12	Gaubert et al. (2021)
20	0.429	51 Cerebellum CB	Superior parietal lobule SPL 12	Weiler et al. (2015)
21	−0.185	53 Cerebellum CB	Cerebellum CB 50	Ibañez et al. (2021)
22	0.217	50 Cerebellum CB	Superior parietal lobule SPL 12	Weiler et al. (2015)

**Table 11:** The table summarizes the number of significant *p* < 0.05 group discriminative multimodal links, maximum positive and negative correlations. The values are reported as Mean ± Standard Error, calculated across folds.

Model	Number of significant links	Max positive correlation	Max negative correlation

Supervised	62.40 ± 18.08	0.46 ± 0.01	−0.47 ± 0.01
AE	22.80 ± 2.13	0.57 ± 0.01	−0.56 ± 0.02
CR-CCA	17.40 ± 3.33	0.52 ± 0.04	−0.50 ± 0.03
DCCAE	18.00 ± 2.59	0.48 ± 0.03	−0.48 ± 0.01
RR	121.40 ± 8.16	**0.86 ± 0.01**	−0.73 ± 0.02
RR-AE	136.80 ± 11.41	0.72 ± 0.02	−0.70 ± 0.01
XX-CC	209.60 ± 23.33	0.77 ± 0.02	−0.79 ± 0.02
RR-XX-CC	**252.00 ± 15.72**	0.85 ± 0.01	**−0.84 ± 0.01**

**Fig. B.18.**
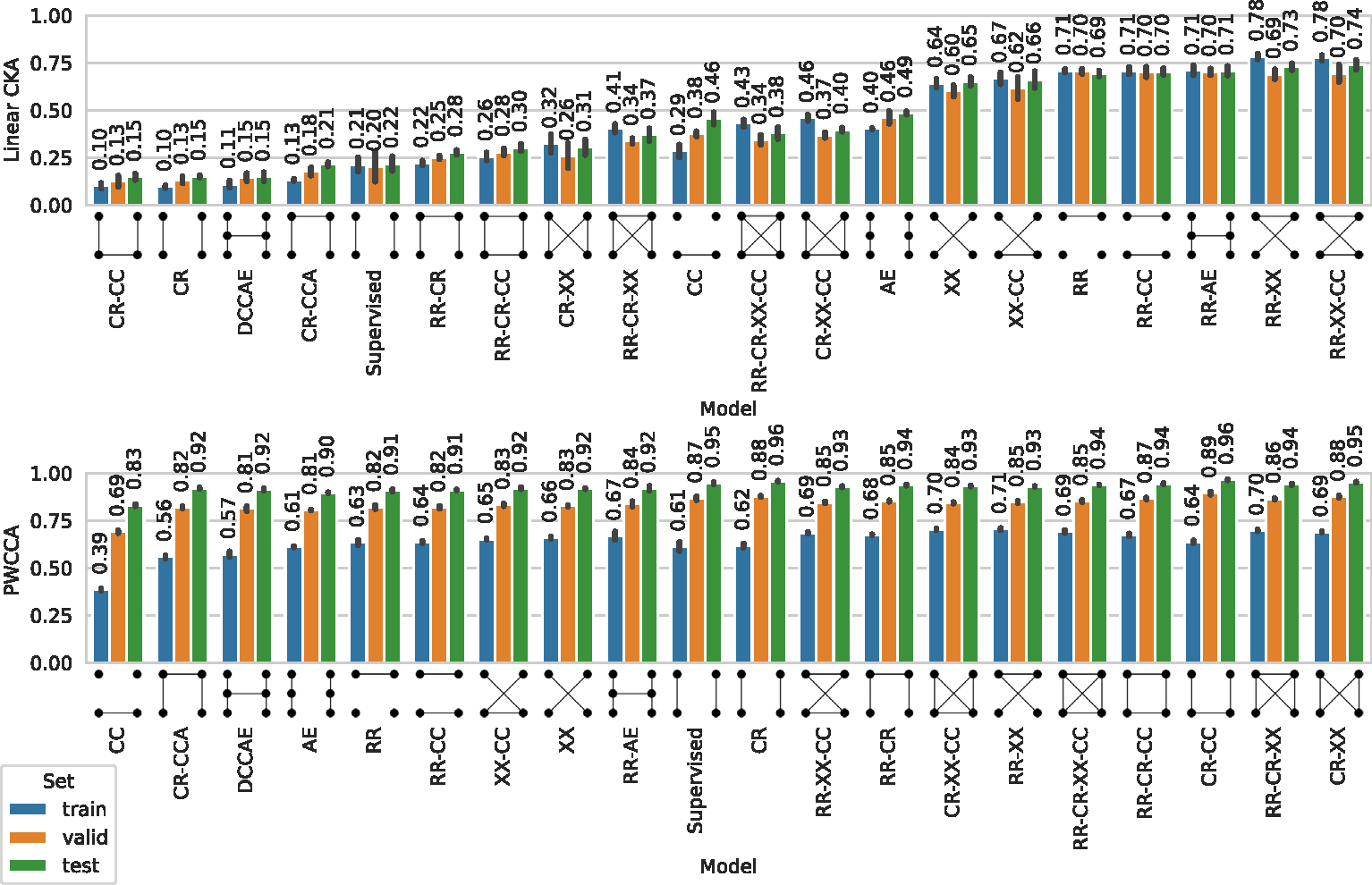
Ranking of the models based on Linear CKA and PWCCA similarity measures. The models are sorted based on the corresponding metric on the test set.

**Fig. D.19.**
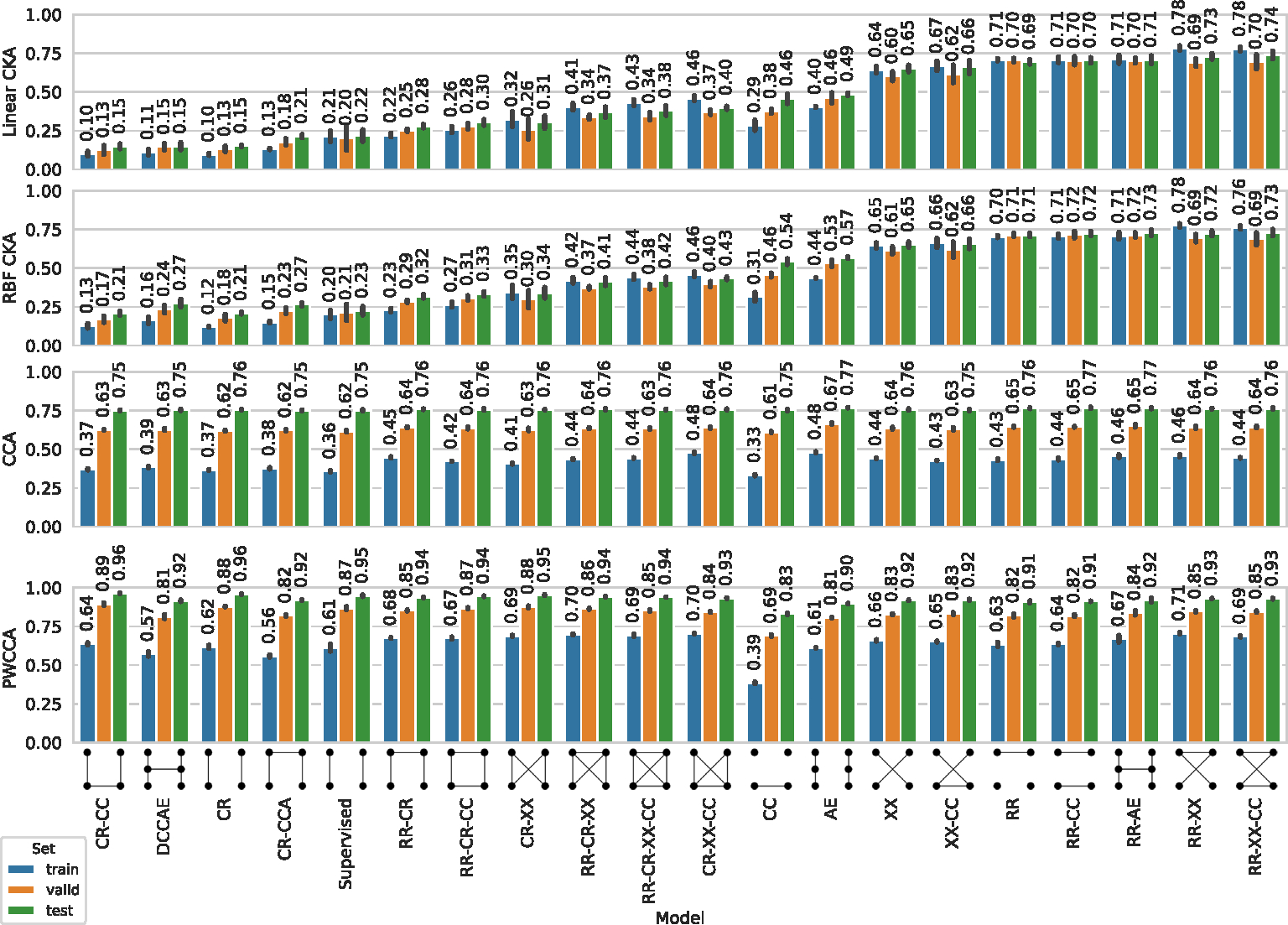
Comparison of the models with Linear CKA, RBF CKA, CCA and PWCCA. The models sorted based on CKA performance on the test set.

## Data Availability

This study does not introduce a new dataset and all datasets used in this study are properly referenced. The code for deep learning models is available at https://github.com/Entodi/fusion.

## Appendix A. Experiments on multi-view and multi-domain datasets with natural images

**Table A.12:** Accuracy on Two-View MNIST dataset. Ranking is based on the median of the Rotated accuracy.

Model	Rotated_ACC_Median	Rotated_ACC_IQR_25	Noisy_ACC_Median	Noisy ACC IQR 25

Supervised	0.9906	0.0000	0.9862	0.0000
XX	0.9773	0.0000	0.9811	0.0000
CR-CCA	0.9752	0.0000	0.9786	0.0000
XX-CC	0.9747	0.0000	0.9818	0.0001
RR-XX-CC	0.9727	0.0000	0.9762	0.0000
XX-RR	0.9716	0.0000	0.9775	0.0000
DCCAE	0.9688	0.0000	0.9662	0.0000
RR-CR-XX	0.9643	0.0000	0.9665	0.0000
RR-CR-XX-CC	0.9631	0.0000	0.9680	0.0000
CR-XX-CC	0.9483	0.0000	0.9434	0.0000
RR-CR	0.9420	0.0001	0.9432	0.0000
RR-CR-CC	0.9418	0.0000	0.9435	0.0000
RR-CC	0.9359	0.0000	0.9483	0.0000
RR	0.9353	0.0000	0.9509	0.0000
RR-AE	0.9335	0.0000	0.9462	0.0000
CR-XX	0.9322	0.0000	0.9189	0.0000
CR-CC	0.8230	0.0013	0.9182	0.0032
AE	0.8175	0.0000	0.8274	0.0000
CR	0.7869	0.0003	0.6704	0.0000
CC	0.7838	0.0000	0.7928	0.0000

See Tables A.12 and A.13.

### A.1. Two-view MNIST

The first dataset used in our experiments is the Two-View MNIST dataset (Wang et al., 2015). This dataset serves as a simple case for the multi-modal scenario, where data is represented as a pair of corrupted images derived from a single source. Initially, image intensities are rescaled to a unit interval and resized to 32 × 32 to fit the DCGAN architecture. The first view is generated by randomly rotating the images at an angle uniformly sampled from $\left[-\frac{\pi }{4},\;\frac{\pi }{4}\right]$. The second view is generated by adding [0,1]-uniform noise to the image and performing an additional intensity rescaling to a unit interval. A cross-validation dataset was created using a stratified 5-fold split from the original 60K training MNIST set, resulting in 48K and 12K images in the training and validation sets, respectively. The original 10K MNIST test set was preserved as a hold-out set.

Table A.12 presents the accuracy (ACC) on the multi-view Two-View MNIST dataset, based on the linear evaluation protocol. The best-performing model is the supervised model. Self-supervised unimodal models AE and CR underperform compared to multimodal objectives, with the exception of CC. CCA-based objectives are clearly outperformed by objectives based on mutual information estimation but still very close by performance. The best-performing models are multimodal: XX and XX-CC, - that are decoder-free and use the mutual information estimator.

### A.2. MNIST-SVHN

The second dataset employed is the multi-domain MNIST-SVHN, proposed by the authors of MMVAE (Shi et al., 2019). Each sample in this dataset represents a single digit as a pair of images sampled from MNIST and SVHN. The dataset generation follows the process described in the MMVAE code (Shi et al., 2019). However, there are two differences from the original work. First, MNIST images are resized to 32 × 32 to fit the DCGAN architecture. Second, the original training set is used to generate cross-validation folds using a stratified 5-fold split.

Table A.13 presents the accuracy (ACC) on the multi-domain MNIST-SVHN dataset, based on the linear evaluation protocol. In this case, the supervised model is outperformed by multiple multimodal objectives: XX-CC, RR-AE, RR, RR-XX-CC, XX-RR, RR-CR-XX, RR-CC, RR-CR-XX-CC, RR-CR, RR-CR-CC, DCCAE. Among these multimodal objectives, XX-CC performs best on the more complicated SVHN dataset than MNIST.

### A.3. Training details

The architecture of the models is similar to the OASIS3 setup, with the only difference being that we use a DCGAN with one less layer, as the input size of images is 32 × 32. The models are trained using RAdam (Liu et al., 2019) with a learning rate lr = 4e−4 and the OneCy-cleLR scheduler (Smith and Topin, 2019) with max_lr = 0.01. The batch size is chosen from 64, 128, and 256 based on best performance on the validation set. The pretraining step in our framework is performed for 50 epochs with the Two-View MNIST and MNIST-SVHN datasets. The linear evaluation step is carried out for 50 epochs.

## Appendix B. Joint linear classification on OASIS3

In this section, we explore the joint linear classification by training a Logistic Regression on the concatenated learned embeddings from both modalities, also known as late fusion (Rahaman et al., 2022). The experiments follow precisely the same strategy as unimodal linear classification.

**Table A.13:** Accuracy on MNIST-SVHN dataset. Ranking is based on the median of the SVHN accuracy as it is more challenging dataset.

Model	MNIST ACC Median	MNIST ACC IQR 25	SVHN ACC Median	SVHN ACC IQR 25

XX-CC	0.9872	0.0040	0.8734	0.0132
XX	0.9883	0.0000	0.8695	0.0000
RR-AE	0.9905	0.0000	0.8677	0.0000
RR	0.9901	0.0000	0.8668	0.0001
RR-XX-CC	0.9912	0.0000	0.8635	0.0000
XX-RR	0.9889	0.0000	0.8627	0.0000
RR-CR-XX	0.9894	0.0000	0.8602	0.0001
RR-CC	0.9916	0.0000	0.8592	0.0000
RR-CR-XX-CC	0.9898	0.0000	0.8558	0.0000
RR-CR	0.9887	0.0000	0.8551	0.0000
RR-CR-CC	0.9892	0.0000	0.8544	0.0000
DCCAE	0.9792	0.0001	0.8423	0.0001
Supervised	0.9889	0.0000	0.8381	0.0000
CR-CCA	0.9819	0.0000	0.8149	0.0000
CR-XX-CC	0.9370	0.0000	0.6354	0.0004
CR-XX	0.9373	0.0000	0.5888	0.0000
CC	0.9217	0.0000	0.3173	0.0000
CR-CC	0.9313	0.0001	0.3158	0.0002
CR	0.9199	0.0000	0.2898	0.0001
AE	0.8977	0.0000	0.1967	0.0000

**Table A.14:** Mean and standard error of ROCAUC for binary joint classification task on a hold-out test set. The significance is computed with the Wilcoxon signed-rank test and additional Holm correction for multiple comparisons.

Model	Baseline	Multimodal	ROCAUC	Significance

**Supervised**	✓		87.3 ± 0.9	N/A

AE	✓		84.5 ± 1.1	*
RR-AE		✓	84.3 ± 0.6	.
CR-XX-CC		✓	84.1 ± 0.5	*
RR-XX		✓	83.5 ± 1.0	*
CR-CCA		✓	82.9 ± 1.2	*
RR-CR-XX		✓	82.5 ± 1.2	*
RR-XX-CC		✓	82.0 ± 1.2	*
RR	✓	✓	81.8 ± 1.0	*
CR-CC		✓	81.8 ± 1.1	*
RR-CC		✓	81.4 ± 1.2	*
DCCAE	✓	✓	81.2 ± 0.6	*
CR-XX		✓	81.1 ± 2.0	*
RR-CR-XX-CC		✓	81.0 ± 1.5	*
RR-CR-CC		✓	79.5 ± 1.8	*
RR-CR		✓	79.4 ± 2.1	*
CR	✓		79.4 ± 1.7	*
XX		✓	79.3 ± 1.0	*
XX-CC	✓	✓	79.1 ± 1.2	*
CC		✓	76.8 ± 2.1	*

The significance is denoted as follows:

*** *p* < 0.001

** *p* < 0.01

* *p* < 0.05

*p* < 0.1. Bold names are the best models based on ROCAUC and significance testing.

The joint classification analysis is also known as fusion in the literature (Liang et al., 2022). There are three types of fusion: early, mid, and late. Early fusion assumes combining modalities at the input level, mid fusion — combination after encoding with initial unimodal nonlinear layers, and combining at the end of the encoder — currently, the state-of-the-art results achieved by mid fusion (Rahaman et al., 2022). In contrast, in our work, we focus on the coordination approach (Liang et al., 2022) with separate encoders. The coordination approach does not mix multiple modalities but coordinates them via joint constraints that we enforced via mutual information maximization or CCA. Hence, we perform only late fusion within our framework. By construction, we should underperform compared to mid-fusion (Rahaman et al., 2022) due to a less expressive model. However, our framework is more interpretable and can efficiently deal with missing modalities.

The results are shown in Table A.14 for binary classification and Table A.15 for ternary classification. Overall, the models preserved relative ranking. Importantly, the proposed models still outperform baselines. However, joint classification did not improve the classification performance drastically.

However, there are two downsides of late fusion by concatenation with Logistic Regression. The aligned representation can have a higher correlations. Hence, it will lead to multicollinearity (see structure of the compared models in Fig. 17). The other problem is that the unimodal logistic regression will only have 64-dimensional features, while multimodal logistic regression will have 128-dimensional features (2 modalities times 64 dimensions). Thus, it will be harder to fit multimodal logistic regression, requiring more data due to complexity. Hence, this topic would require extensive further research that is beyond the scope of this work.

## Appendix C. Ablation for the multimodal alignment

To explore the similarity between multimodal representations, we proposed to use CKA over CCA. It has been shown (Kornblith et al., 2019) that the Canonical Correlation Analysis (CCA) method does not allow reliable identification of the correspondence between the representations of different neural networks or layers. To further support our choice for alignment measure, we compare CKA with projection-weighted CCA (PWCCA) (Morcos et al., 2018). The PWCCA is shown to improve over standard CCA by weighting canonical coefficients to improve the estimation of the joint content.

**Table A.15:** Mean and standard error of OVO ROCAUC Macro for ternary joint classification task on a hold-out test set. The significance is computed with the Wilcoxon signed-rank test and additional Holm correction for multiple comparisons.

Model	Baseline	Multimodal	OVO ROCAUC Macro	Significance

**Supervised**	✓		71.6 ± 2.2	N/A
**CR-XX**		✓	67.4 ± 1.5	
**RR-XX**		✓	67.3 ± 2.1	
**CR-XX-CC**		✓	67.2 ± 1.9	
**RR-XX-CC**		✓	67.1 ± 1.7	.

AE	✓		66.9 ± 1.6	
RR-CR-XX-CC		✓	66.5 ± 2.3	
RR-AE		✓	66.1 ± 1.1	.
XX		✓	66.0 ± 2.1	.
RR-CR-XX		✓	64.9 ± 2.6	.
DCCAE	✓	✓	64.7 ± 0.6	*
RR-CR		✓	63.9 ± 1.4	*
RR-CC		✓	63.7 ± 0.9	*
CC		✓	63.5 ± 1.0	*
RR-CR-CC		✓	62.5 ± 1.7	*
RR	✓	✓	62.3 ± 1.1	*
CR-CCA		✓	62.2 ± 2.6	*
XX-CC	✓	✓	62.0 ± 3.5	*
CR-CC		✓	61.0 ± 2.2	*
CR	✓		59.9 ± 2.5	*

The significance is denoted as follows:

*** *p* < 0.001

** *p* < 0.01

* *p* < 0.05

*p* < 0.1. Bold names are the best models based on ROCAUC and significance testing.

**Table E.16:** The table for the Fig. E.20 provides detailed quantitative information on the near absolute maximum (quantile 0.999) Peak RBC values of the saliency for regions in the Neuromark atlas for the dimensions with non-zero importance (Betas in Logistic Regression), derived from the best fold on binary classification.

Model	Region	Supervised		RR-XX-CC	
		
Modality		T1	fALFF	T1	fALFF

0	1 Caudate	0.61	0.44	–	−0.28
1	2 Subthalamus hypothalamus	0.64	0.43	–	−0.30
2	3 Putamen	−0.61	0.44	–	0.29
3	4 Caudate	–	0.44	–	−0.28
4	5 Thalamus	0.60	0.43	0.43	−0.28
5	6 Superior temporal gyrus STG	–	−0.42	−0.47	−0.29
6	7 Middle temporal gyrus MTG	−0.67	−0.34	0.53	0.27
7	8 Postcentral gyrus PoCG	−0.63	0.43	0.45	0.27
8	9 Left postcentral gyrus L PoCG	0.61	−0.34	−0.56	−0.30
9	10 Paracentral lobule ParaCL	−0.65	−0.39	0.51	−0.29
10	11 Right postcentral gyrus R PoCG	0.55	−0.43	–	0.29
11	12 Superior parietal lobule SPL	0.67	−0.34	−0.54	0.34
12	13 Paracentral lobule ParaCL	0.55	−0.33	0.48	−0.34
13	14 Precentral gyrus PreCG	0.63	−0.30	−0.50	−0.34
14	15 Superior parietal lobule SPL	−0.53	–	0.51	0.29
15	16 Postcentral gyrus PoCG	−0.56	0.35	0.43	−0.32
16	17 Calcarine gyrus CalcarineG	0.67	−0.36	0.55	0.26
17	18 Middle occipital gyrus MOG	0.52	−0.33	−0.45	0.33
18	19 Middle temporal gyrus MTG	−0.54	–	–	−0.26
19	20 Cuneus	−0.57	−0.32	0.48	−0.32
20	21 Right middle occipital gyrus R MOG	−0.52	0.34	–	0.25
21	22 Fusiform gyrus	0.53	−0.35	–	−0.30
22	23 Inferior occipital gyrus IOG	0.53	0.32	0.46	−0.31
23	24 Lingual gyrus LingualG	0.61	0.39	0.50	−0.32
24	25 Middle temporal gyrus MTG	−0.60	−0.33	0.45	−0.30
25	26 Inferior parietal lobule IPL	−0.59	−0.34	–	0.25
26	27 Insula	0.62	−0.33	−0.57	−0.32
27	28 Superior medial frontal gyrus SMFG	–	0.39	−0.45	–
28	29 Inferior frontal gyrus IFG	−0.58	0.43	0.52	0.29
29	30 Right inferior frontal gyrus R IFG	0.62	−0.42	–	0.29
30	31 Middle frontal gyrus MiFG	0.55	−0.44	0.44	−0.26
31	32 Inferior parietal lobule IPL	0.59	0.44	0.47	0.28
32	33 Left inferior parietal lobue R IPL	0.60	0.39	0.43	−0.33
33	34 Supplementary motor area SMA	0.55	−0.40	–	0.32
34	35 Superior frontal gyrus SFG	–	0.39	−0.46	−0.32
35	36 Middle frontal gyrus MiFG	−0.57	−0.43	–	0.25
36	37 Hippocampus HiPP	−0.58	−0.42	0.45	−0.27
37	38 Left inferior parietal lobule L IPL	0.55	0.33	−0.45	–
38	39 Middle cingulate cortex MCC	0.55	−0.40	–	0.29
39	40 Inferior frontal gyrus IFG	−0.58	−0.40	0.43	−0.28
40	41 Middle frontal gyrus MiFG	–	–	–	0.30
41	42 Hippocampus HiPP	–	–	–	−0.22
42	43 Precuneus	0.55	0.43	−0.47	0.26
43	44 Precuneus	0.56	0.45	0.44	−0.28
44	45 Anterior cingulate cortex ACC	−0.59	0.45	0.45	−0.28
45	46 Posterior cingulate cortex PCC	0.57	0.43	−0.47	−0.26
46	47 Anterior cingulate cortex ACC	−0.63	−0.38	0.49	−0.26
47	48 Precuneus	−0.64	−0.40	0.49	−0.31
48	49 Posterior cingulate cortex PCC	−0.66	−0.41	0.55	0.33
49	50 Cerebellum CB	0.63	−0.32	0.55	−0.35
50	51 Cerebellum CB	0.66	–	−0.54	0.32
51	52 Cerebellum CB	0.67	0.44	0.49	−0.27
52	53 Cerebellum CB	0.59	−0.38	0.51	−0.33

The ablations results are shown in Fig. B.18. The models are sorted based on the corresponding metric. Overall, both PWCCA and Linear CKA agree that the proposed methodologies capture higher content of the joint information between modalities. However, the CKA emphasizes the divergent inductive biases of each model compared to PWCCA. Additionally, PWCCA lacks stability when applied to different subsets of the data. Hence, based on this exploration, we suggest using CKA to measure alignment between modalities.

Full results with Linear CKA, RBF CKA, CCA, and PWCCA are shown in Fig. D.19.

## Appendix D. Explaining group differences between HC and AD

In Fig. 12 for Supervised vs. RR-XX-CC, we explored only the two most significant (highest positive and lowest negative) dimensions from 64 available for the sake of showcasing the interpretability. However, if we use all 64 except dimensions with zero importance and select peak RBC by absolute value, the annulus looks as in Fig. E.20 with supporting Table E.16. We capture hippocampus (37) and the default mode network more consistently across all models.

## Appendix E. Comparing self-supervised models on group discriminative regions

Similarly, in Fig. 16, we explored only the two most significant (highest positive and lowest negative) dimensions from 64 available for the sake of showcasing the interpretability. However, if we use all 64 except dimensions with zero importance and select peak RBC by absolute value, the annulus looks as in Fig. F.21. We capture hippocampus (37) and the default mode network more consistently across all models.

## References

[R1] Abrol A, Fu Z, Salman M, Silva R, Du Y, Plis S, Calhoun V, 2021. Deep learning encodes robust discriminative neuroimaging representations to outperform standard machine learning. Nat. Commun. 12 (1), 1–17. 33441557 10.1038/s41467-020-20655-6 PMC7806588

[R2] Agosta F, Rocca MA, Pagani E, Absinta M, Magnani G, Marcone A, Falautano M, Comi G, Gorno-Tempini ML, Filippi M, 2010. Sensorimotor network rewiring in mild cognitive impairment and alzheimer’s disease. Hum. Brain Mapp. 31 (4), 515–525. 19777557 10.1002/hbm.20883 PMC6871105

[R3] Akiba T, Sano S, Yanase T, Ohta T, Koyama M, 2019. Optuna: A next-generation hyperparameter optimization framework. In: ICKDM.

[R4] Alain G, Bengio Y, 2016. Understanding intermediate layers using linear classifier probes. arXiv preprint arXiv:1610.01644.

[R5] Alayrac J-B, Recasens A, Schneider R, Arandjelović R, Ramapuram J, De Fauw J, Smaira L, Dieleman S, Zisserman A, 2020. Self-supervised multimodal versatile networks. Adv. Neural Inf. Process. Syst. 33, 25–37.

[R6] Anand A, Racah E, Ozair S, Bengio Y, Côté M, Hjelm D, 2019. Unsupervised state representation learning in atari. In: NeurIPS.

[R7] Andrew G, Arora R, Bilmes J, Livescu K, 2013. Deep canonical correlation analysis. In: International Conference on Machine Learning. PMLR, pp. 1247–1255.

[R8] Araujo A, Norris W, Sim J, 2019. Computing receptive fields of convolutional neural networks. Distill http://dx.doi.org/10.23915/distill.00021, https://distill.pub/2019/computing-receptive-fields.

[R9] Arpit D, Jastrzębski S, Ballas N, Krueger D, Bengio E, Kanwal MS, Maharaj T, Fischer A, Courville A, Bengio Y, , 2017. A closer look at memorization in deep networks. In: International Conference on Machine Learning. PMLR, pp. 233–242.

[R10] Bachman P, Hjelm RD, Buchwalter W, 2019. Learning representations by maximizing mutual information across views. In: Wallach H, Larochelle H, Beygelzimer A, d’Alché Buc F, Fox E, Garnett R (Eds.), Advances in Neural Information Processing Systems. 32, Curran Associates, Inc..

[R11] Baltrušaitis T, Ahuja C, Morency L-P, 2018. Multimodal machine learning: A survey and taxonomy. IEEE Trans. Pattern Anal. Mach. Intell. 41 (2), 423–443. 29994351 10.1109/TPAMI.2018.2798607

[R12] Behfar Q, Behfar SK, Von Reutern B, Richter N, Dronse J, Fassbender R, Fink GR, Onur OA, 2020. Graph theory analysis reveals resting-state compensatory mechanisms in healthy aging and prodromal alzheimer’s disease. Front. Aging Neurosci. 12, 576627. 33192468 10.3389/fnagi.2020.576627 PMC7642892

[R13] Berron D, van Westen D, Ossenkoppele R, Strandberg O, Hansson O, 2020. Medial temporal lobe connectivity and its associations with cognition in early alzheimer’s disease. Brain 143 (4), 1233–1248. 32252068 10.1093/brain/awaa068 PMC7174043

[R14] Bi X. a., Wu H, Xie Y, Zhang L, Luo X, Fu Y, Alzheimer’s Disease Neuroimaging Initiative, 2021. The exploration of parkinson’s disease: a multi-modal data analysis of resting functional magnetic resonance imaging and gene data. Brain Imag. Behav. 15, 1986–1996. 10.1007/s11682-020-00392-6 32990896

[R15] Calhoun VD, Sui J, 2016. Multimodal fusion of brain imaging data: a key to finding the missing link (s) in complex mental illness. Biol. Psychiatry Cogn. Neurosc. Neuroimag. 1 (3), 230–244. 10.1016/j.bpsc.2015.12.005 PMC4917230 27347565

[R16] Caron M, Misra I, Mairal J, Goyal P, Bojanowski P, Joulin A, 2020. Unsupervised learning of visual features by contrasting cluster assignments. In: Larochelle H, Ranzato M, Hadsell R, Balcan MF, Lin H (Eds.), Advances in Neural Information Processing Systems, Vol. 33. Curran Associates, Inc., pp. 9912–9924.

[R17] Casula EP, Borghi I, Maiella M, Pellicciari MC, Bonnì S, Mencarelli L, Assogna M, D’Acunto A, Di Lorenzo F, Spampinato DA, , 2023. Regional precuneus cortical hyperexcitability in Alzheimer’s disease patients. Ann. Neurol. 93 (2), 371–383. 36134540 10.1002/ana.26514 PMC10092632

[R18] Chen T, Kornblith S, Norouzi M, Hinton G, 2020. A simple framework for contrastive learning of visual representations. In: International Conference on Machine Learning. PMLR, pp. 1597–1607.

[R19] Chen P, Zhao K, Zhang H, Wei Y, Wang P, Wang D, Song C, Yang H, Zhang Z, Yao H, , 2023. Altered global signal topography in Alzheimer’s disease. Ebiomedicine 89. 10.1016/j.ebiom.2023.104455 PMC9941064 36758481

[R20] Chételat G, Mézenge F, Tomadesso C, Landeau B, Arenaza-Urquijo E, Rauchs G, André C, De Flores R, Egret S, Gonneaud J, , 2017. Reduced age-associated brain changes in expert meditators: a multimodal neuroimaging pilot study. Sci. Rep. 7 (1), 10160. 28860449 10.1038/s41598-017-07764-x PMC5578985

[R21] Cheung EY, Shea Y, Chiu PK, Kwan JS, Mak HK, 2021. Diagnostic efficacy of voxel-mirrored homotopic connectivity in vascular dementia as compared to alzheimer’s related neurodegenerative diseases—A resting state fMRI study. Life 11 (10), 1108. 34685479 10.3390/life11101108 PMC8538280

[R22] Comon P, 1994. Independent component analysis, a new concept? Signal Process. 36 (3), 287–314.

[R23] Corriveau-Lecavalier N, Duchesne S, Gauthier S, Hudon C, Kergoat M-J, Mellah S, Belleville S, Consortium for the Early Identification of Alzheimer’s Disease-Quebec (CIMA-Q), 2020. A quadratic function of activation in individuals at risk of alzheimer’s disease. Alzheimer’s Dementia Diagn. Assess. Disease Monitor. 12 (1), e12139. 10.1002/dad2.12139 PMC7817778 33521234

[R24] Coupé P, Manjón JV, Lanuza E, Catheline G, 2019. Lifespan changes of the human brain in Alzheimer’s disease. Sci. Rep. 9 (1), 3998. 30850617 10.1038/s41598-019-39809-8 PMC6408544

[R25] Defazio A, Bach F, Lacoste-Julien S, 2014. SAGA: A fast incremental gradient method with support for non-strongly convex composite objectives. arXiv preprint arXiv:1407.0202.

[R26] Doersch C, Gupta A, Efros AA, 2015. Unsupervised visual representation learning by context prediction. In: Proceedings of the IEEE International Conference on Computer Vision. pp. 1422–1430.

[R27] Dosovitskiy A, Springenberg JT, Riedmiller M, Brox T, 2014. Discriminative unsupervised feature learning with convolutional neural networks. Adv. Neural Inf. Process. Syst 27. 10.1109/TPAMI.2015.2496141 26540673

[R28] Du Y, Fu Z, Sui J, Gao S, Xing Y, Lin D, Salman M, Abrol A, Rahaman MA, Chen J, , 2020. NeuroMark: An automated and adaptive ICA based pipeline to identify reproducible fMRI markers of brain disorders. NeuroImage: Clin. 28, 102375. 32961402 10.1016/j.nicl.2020.102375 PMC7509081

[R29] Duan K, Calhoun VD, Liu J, Silva RF, 2020. aNy-way independent component analysis. In: 2020 42nd Annual International Conference of the IEEE Engineering in Medicine & Biology Society. EMBC, IEEE, pp. 1770–1774. 10.1109/EMBC44109.2020.9175277 PMC8258844 33018341

[R30] Ekblad LL, Visser PJ, Tijms BM, 2020. Biological substrates of cortical atrophy in prodromal AD: A CSF proteomic study: Biomarkers (non-neuroimaging)/multi-modal comparisons. Alzheimer’s Dement. 16, e042894.

[R31] Fedorov A, Hjelm RD, Abrol A, Fu Z, Du Y, Plis S, Calhoun VD, 2019. Prediction of progression to alzheimer’s disease with deep infomax. In: 2019 IEEE EMBS International Conference on Biomedical & Health Informatics. BHI, IEEE, pp. 1–5.

[R32] Fedorov A, Johnson J, Damaraju E, Ozerin A, Calhoun V, Plis S, 2017. End-to-end learning of brain tissue segmentation from imperfect labeling. In: 2017 International Joint Conference on Neural Networks. IJCNN, IEEE, pp. 3785–3792.

[R33] Fedorov A, Sylvain T, Geenjaar E, Luck M, Wu L, DeRamus TP, Kirilin A, Bleklov D, Calhoun VD, Plis SM, 2021. Self-supervised multimodal domino: in search of biomarkers for alzheimer’s disease. In: 2021 IEEE 9th International Conference on Healthcare Informatics. ICHI, IEEE, pp. 23–30.

[R34] Feng C, Elazab A, Yang P, Wang T, Zhou F, Hu H, Xiao X, Lei B, 2019a. Deep learning framework for Alzheimer’s disease diagnosis via 3D-CNN and FSBi-LSTM. IEEE Access 7, 63605–63618.

[R35] Feng Z, Xu C, Tao D, 2019b. Self-supervised representation learning from multi-domain data. In: Proceedings of the IEEE/CVF International Conference on Computer Vision. pp. 3245–3255.

[R36] Filippi M, Basaia S, Canu E, Imperiale F, Magnani G, Falautano M, Comi G, Falini A, Agosta F, 2020. Changes in functional and structural brain connectome along the alzheimer’s disease continuum. Mol Psychiatry 25 (1), 230–239. 29743583 10.1038/s41380-018-0067-8

[R37] Frisoni GB, Altomare D, Thal DR, Ribaldi F, van der Kant R, Ossenkoppele R, Blennow K, Cummings J, van Duijn C, Nilsson PM, , 2022. The probabilistic model of alzheimer disease: the amyloid hypothesis revised. Nat. Rev. Neurosci. 23 (1), 53–66. 34815562 10.1038/s41583-021-00533-w PMC8840505

[R38] Frisoni GB, Ganzola R, Canu E, Rüb U, Pizzini FB, Alessandrini F, Zoccatelli G, Beltramello A, Caltagirone C, Thompson PM, 2008. Mapping local hippocampal changes in alzheimer’s disease and normal ageing with MRI at 3 Tesla. Brain 131 (12), 3266–3276. 18988639 10.1093/brain/awn280

[R39] Frome A, Corrado GS, Shlens J, Bengio S, Dean J, Ranzato M, Mikolov T, 2013. Devise: A deep visual-semantic embedding model. Adv. Neural Inf. Process. Syst 26.

[R40] Gaubert M, Lange C, Garnier-Crussard A, Köbe T, Bougacha S, Gonneaud J, de Flores R, Tomadesso C, Mézenge F, Landeau B, , 2021. Topographic patterns of white matter hyperintensities are associated with multimodal neuroimaging biomarkers of alzheimer’s disease. Alzheimer’s Res. Therapy 13, 1–11. 10.1186/s13195-020-00759-3 PMC7814451 33461618

[R41] Geirhos R, Jacobsen J, Michaelis C, Zemel R, Brendel W, Bethge M, Wichmann F, 2020. Shortcut learning in deep neural networks. arXiv preprint arXiv: 2004.07780.

[R42] Gidaris S, Singh P, Komodakis N, 2018. Unsupervised representation learning by predicting image rotations. arXiv preprint arXiv:1803.07728.

[R43] Glorot X, Bengio Y, 2010. Understanding the difficulty of training deep feedforward neural networks. In: Proceedings of the Thirteenth International Conference on Artificial Intelligence and Statistics. JMLR Workshop and Conference Proceedings, pp. 249–256.

[R44] Greene SJ, Killiany RJ, Alzheimer’s Disease Neuroimaging Initiative, , 2010. Subregions of the inferior parietal lobule are affected in the progression to alzheimer’s disease. Neurobiol. Aging 31 (8), 1304–1311. 20570398 10.1016/j.neurobiolaging.2010.04.026 PMC2907057

[R45] Greicius MD, Srivastava G, Reiss AL, Menon V, 2004. Default-mode network activity distinguishes alzheimer’s disease from healthy aging: evidence from functional MRI. Proc. Natl. Acad. Sci. 101 (13), 4637–4642. 15070770 10.1073/pnas.0308627101 PMC384799

[R46] Gretton A, Bousquet O, Smola A, Schölkopf B, 2005. Measuring statistical dependence with Hilbert-Schmidt norms. In: International Conference on Algorithmic Learning Theory. Springer, pp. 63–77.

[R47] Gu L, Zhang Z, 2019. Exploring structural and functional brain changes in mild cognitive impairment: a whole brain ALE meta-analysis for multimodal MRI. ACS Chem. Neurosci. 10 (6), 2823–2829. 30808171 10.1021/acschemneuro.9b00045

[R48] Guennewig B, Lim J, Marshall L, McCorkindale AN, Paasila PJ, Patrick E, Kril JJ, Halliday GM, Cooper AA, Sutherland GT, 2021. Defining early changes in alzheimer’s disease from RNA sequencing of brain regions differentially affected by pathology. Sci. Rep. 11 (1), 4865. 33649380 10.1038/s41598-021-83872-z PMC7921390

[R49] Guerrier L, Le Men J, Gane A, Planton M, Salabert A-S, Payoux P, Dumas H, Bonneville F, Péran P, Pariente J, 2018. Involvement of the cingulate cortex in anosognosia: A multimodal neuroimaging study in alzheimer’s disease patients. J. Alzheimer’s Dis. 65 (2), 443–453. 30056422 10.3233/JAD-180324 PMC6130407

[R50] Hallam B, Chan J, Costafreda SG, Bhome R, Huntley J, 2020. What are the neural correlates of meta-cognition and anosognosia in alzheimer’s disease? A systematic review. Neurobiol. Aging 94, 250–264. 32679396 10.1016/j.neurobiolaging.2020.06.011 PMC7903321

[R51] Hand DJ, Till RJ, 2001. A simple generalisation of the area under the ROC curve for multiple class classification problems. Mach. Learn. 45 (2), 171–186.

[R52] He K, Fan H, Wu Y, Xie S, Girshick R, 2020. Momentum contrast for unsupervised visual representation learning. In: Proceedings of the IEEE/CVF Conference on Computer Vision and Pattern Recognition. pp. 9729–9738.

[R53] He Y, Wang L, Zang Y, Tian L, Zhang X, Li K, Jiang T, 2007. Regional coherence changes in the early stages of alzheimer’s disease: a combined structural and resting-state functional MRI study. Neuroimage 35 (2), 488–500. 17254803 10.1016/j.neuroimage.2006.11.042

[R54] Hénaff OJ, Srinivas A, De Fauw J, Razavi A, Doersch C, Eslami S, Oord A.v.d., 2020. Data-efficient image recognition with contrastive predictive coding. In: International Conference on Machine Learning. PMLR, pp. 4182–4192.

[R55] Hendrycks D, Mazeika M, Kadavath S, Song D, 2019. Using self-supervised learning can improve model robustness and uncertainty. In: Wallach H, Larochelle H, Beygelzimer A, d’Alché Buc F, Fox E, Garnett R (Eds.), Advances in Neural Information Processing Systems, Vol. 32. Curran Associates, Inc..

[R56] Henschel L, Conjeti S, Estrada S, Diers K, Fischl B, Reuter M, 2020. Fastsurfer-a fast and accurate deep learning based neuroimaging pipeline. NeuroImage 219, 117012. 32526386 10.1016/j.neuroimage.2020.117012 PMC7898243

[R57] Hermans A, Beyer L, Leibe B, 2017. In defense of the triplet loss for person re-identification. arXiv preprint arXiv:1703.07737.

[R58] Hinkle DE, Wiersma W, Jurs SG, 1988. Solutions Manual: Applied Statistics for the Behavioral Sciences. Houghton Mifflin.

[R59] Hirono N, Mori E, Ishii K, Ikejiri Y, Imamura T, Shimomura T, Hashimoto M, Yamashita H, Sasaki M, 1998. Frontal lobe hypometabolism and depression in alzheimer’s disease. Neurology 50 (2), 380–383. 9484357 10.1212/wnl.50.2.380

[R60] Hjelm RD, Calhoun VD, Salakhutdinov R, Allen EA, Adali T, Plis SM, 2014. Restricted Boltzmann machines for neuroimaging: an application in identifying intrinsic networks. NeuroImage 96, 245–260. 24680869 10.1016/j.neuroimage.2014.03.048 PMC4348021

[R61] Hjelm RD, Fedorov A, Lavoie-Marchildon S, Grewal K, Bachman P, Trischler A, Bengio Y, 2019. Learning deep representations by mutual information estimation and maximization. In: International Conference on Learning Representations.

[R62] Hotelling H, 1992. Relations between two sets of variates. In: Breakthroughs in Statistics. Springer, pp. 162–190.

[R63] Hu W, Cai B, Zhang A, Calhoun VD, Wang Y-P, 2019a. Deep collaborative learning with application to the study of multimodal brain development. IEEE Trans. Biomed. Eng. 66 (12), 3346–3359. 30872216 10.1109/TBME.2019.2904301 PMC8177041

[R64] Hu B, Yu Y, Dai Y. j., Feng J. h., Yan L-F, Sun Q, Zhang J, Yang Y, Hu Y-C, Nan H-Y, , 2019b. Multi-modal MRI reveals the neurovascular coupling dysfunction in chronic migraine. Neuroscience 419, 72–82. 31682827 10.1016/j.neuroscience.2019.09.022

[R65] Ibañez A, Fittipaldi S, Trujillo C, Jaramillo T, Torres A, Cardona JF, Rivera R, Slachevsky A, García A, Bertoux M, , 2021. Predicting and characterizing neurodegenerative subtypes with multimodal neurocognitive signatures of social and cognitive processes. J. Alzheimer’s Dis. 83 (1), 227–248. 34275897 10.3233/JAD-210163 PMC8461708

[R66] Jang H, Kwon H, Yang J-J, Hong J, Kim Y, Kim KW, Lee JS, Jang YK, Kim ST, Lee KH, , 2017. Correlations between gray matter and white matter degeneration in pure alzheimer’s disease, pure subcortical vascular dementia, and mixed dementia. Sci. Rep. 7 (1), 9541. 28842654 10.1038/s41598-017-10074-x PMC5573310

[R67] Jenkinson M, Bannister P, Brady M, Smith S, 2002. Improved optimization for the robust and accurate linear registration and motion correction of brain images. Neuroimage 17 (2), 825–841. 12377157 10.1016/s1053-8119(02)91132-8

[R68] Jin C, Guo Z, Lin Y, Luo L, Chen H, 2023. Label-efficient deep learning in medical image analysis: Challenges and future directions. arXiv preprint arXiv:2303.12484.

[R69] Kim J, Kim Y-H, Lee J-H, 2013. Hippocampus–precuneus functional connectivity as an early sign of alzheimer’s disease: A preliminary study using structural and functional magnetic resonance imaging data. Brain Res. 1495, 18–29. 23247063 10.1016/j.brainres.2012.12.011

[R70] Kingma DP, Welling M, 2013. Auto-encoding variational bayes. arXiv preprint arXiv:1312.6114.

[R71] Kirkpatrick J, Pascanu R, Rabinowitz N, Veness J, Desjardins G, Rusu AA, Milan K, Quan J, Ramalho T, Grabska-Barwinska A, , 2017. Overcoming catastrophic forgetting in neural networks. Proc. Natl Acad Sci 114 (13), 3521–3526. 28292907 10.1073/pnas.1611835114 PMC5380101

[R72] Kolbeinsson A, Filippi S, Panagakis Y, Matthews PM, Elliott P, Dehghan A, Tzoulaki I, 2020. Accelerated MRI-predicted brain ageing and its associations with cardiometabolic and brain disorders. Sci. Rep. 10 (1), 19940. 33203906 10.1038/s41598-020-76518-z PMC7672070

[R73] Kolesnikov S, 2018. Accelerated deep learning R&D. https://github.com/catalyst-team/catalyst.

[R74] Kornblith S, Norouzi M, Lee H, Hinton G, 2019. Similarity of neural network representations revisited. In: International Conference on Machine Learning. PMLR, pp. 3519–3529.

[R75] LaMontagne PJ, Benzinger TL, Morris JC, Keefe S, Hornbeck R, Xiong C, Grant E, Hassenstab J, Moulder K, Vlassenko AG, Raichle ME, Cruchaga C, Marcus D, 2019. OASIS-3: Longitudinal neuroimaging, clinical, and cognitive dataset for normal aging and alzheimer disease. 10.1101/2019.12.13.19014902, medRxiv, 10.1101/2019.12.13.19014902.

[R76] Laxton AW, Tang-Wai DF, McAndrews MP, Zumsteg D, Wennberg R, Keren R, Wherrett J, Naglie G, Hamani C, Smith GS, , 2010. A phase I trial of deep brain stimulation of memory circuits in alzheimer’s disease. Ann. Neurol. 68 (4), 521–534. 20687206 10.1002/ana.22089

[R77] Li C, Yang J, Zhang P, Gao M, Xiao B, Dai X, Yuan L, Gao J, 2021. Efficient self-supervised vision transformers for representation learning. arXiv preprint arXiv: 2106.09785.

[R78] Liang L, Chen Z, Wei Y, Tang F, Nong X, Li C, Yu B, Duan G, Su J, Mai W, , 2021. Fusion analysis of gray matter and white matter in subjective cognitive decline and mild cognitive impairment by multimodal CCA-joint ICA. NeuroImage: Clin. 32, 102874. 10.1016/j.nicl.2021.102874 PMC8605254 34911186

[R79] Liang PP, Zadeh A, Morency L-P, 2022. Foundations and recent trends in multimodal machine learning: Principles, challenges, and open questions. arXiv preprint arXiv:2209.03430.

[R80] Liu R, Azabou M, Dabagia M, Lin C-H, Gheshlaghi Azar M, Hengen K, Valko M, Dyer E, 2021. Drop, swap, and generate: A self-supervised approach for generating neural activity. Adv. Neural Inf. Process. Syst 34. PMC9713686 36467015

[R81] Liu X, Chen W, Hou H, Chen X, Zhang J, Liu J, Guo Z, Bai G, 2017. Decreased functional connectivity between the dorsal anterior cingulate cortex and lingual gyrus in Alzheimer’s disease patients with depression. Behav. Brain Res. 326, 132–138. 28126471 10.1016/j.bbr.2017.01.037

[R82] Liu L, Jiang H, He P, Chen W, Liu X, Gao J, Han J, 2019. On the variance of the adaptive learning rate and beyond. arXiv preprint arXiv:1908.03265.

[R83] Liu J, Pearlson G, Windemuth A, Ruano G, Perrone-Bizzozero NI, Calhoun V, 2009. Combining fMRI and SNP data to investigate connections between brain function and genetics using parallel ICA. Hum. Brain Mapp. 30 (1), 241–255. 18072279 10.1002/hbm.20508 PMC2668960

[R84] Liu L, Wang T, Du X, Zhang X, Xue C, Ma Y, Wang D, 2022. Concurrent structural and functional patterns in patients with amnestic mild cognitive impairment. Front. Aging Neurosci. 14, 838161. 35663572 10.3389/fnagi.2022.838161 PMC9161636

[R85] Löwe S, O’Connor P, Veeling B, 2019. Putting an end to end-to-end: Gradient-isolated learning of representations. Adv. Neural Inf. Process. Syst 32.

[R86] Lyu Q, Fu X, Wang W, Lu S, 2021. Understanding latent correlation-based multi-view learning and self-supervision: An identifiability perspective. In: International Conference on Learning Representations.

[R87] Mahmood U, Rahman MM, Fedorov A, Fu Z, Plis S, 2019. Transfer learning of fMRI dynamics. arXiv preprint arXiv:1911.06813.

[R88] Mahmood U, Rahman MM, Fedorov A, Lewis N, Fu Z, Calhoun VD, Plis SM, 2020. Whole MILC: generalizing learned dynamics across tasks, datasets, and populations. In: International Conference on Medical Image Computing and Computer-Assisted Intervention. Springer, pp. 407–417.

[R89] Martí-Juan G, Lorenzi M, Piella G, Alzheimer’s Disease Neuroimaging Initiative, , 2023. MC-RVAE: Multi-channel recurrent variational autoencoder for multimodal alzheimer’s disease progression modelling. NeuroImage 268, 119892. 36682509 10.1016/j.neuroimage.2023.119892

[R90] Miech A, Alayrac J-B, Smaira L, Laptev I, Sivic J, Zisserman A, 2020. End-to-end learning of visual representations from uncurated instructional videos. In: Proceedings of the IEEE/CVF Conference on Computer Vision and Pattern Recognition. pp. 9879–9889.

[R91] Minoshima S, Foster NL, Kuhl DE, 1994. Posterior cingulate cortex in Alzheimer’s disease. Elsevier. 10.1016/s0140-6736(94)92871-1 7916431

[R92] Misra I, Maaten L.v.d., 2020. Self-supervised learning of pretext-invariant representations. In: Proceedings of the IEEE/CVF Conference on Computer Vision and Pattern Recognition. pp. 6707–6717.

[R93] Moosmann M, Eichele T, Nordby H, Hugdahl K, Calhoun VD, 2008. Joint independent component analysis for simultaneous EEG–fMRI: principle and simulation. Int. J. Psychophysiol 67 (3), 212–221. 17688965 10.1016/j.ijpsycho.2007.05.016 PMC2649876

[R94] Morcos AS, Raghu M, Bengio S, 2018. Insights on representational similarity in neural networks with canonical correlation. In: NeurIPS. pp. 5732–5741.

[R95] Nguyen T, Raghu M, Kornblith S, 2021. Do wide and deep networks learn the same things? Uncovering how neural network representations vary with width and depth. In: International Conference on Learning Representations.

[R96] Oord A, Li Y, Vinyals O, 2018. Representation learning with contrastive predictive coding. arXiv preprint arXiv:1807.03748.

[R97] Parker AF, Smart CM, Scarapicchia V, Gawryluk JR, Alzheimer’s Disease Neuroimaging Initiative, , 2020. Identification of earlier biomarkers for Alzheimer’s disease: a multimodal neuroimaging study of individuals with subjective cognitive decline. J. Alzheimer’s Dis. 77 (3), 1067–1076. 32804127 10.3233/JAD-200299

[R98] Paszke A, Gross S, Massa F, Lerer A, Bradbury J, Chanan G, Killeen T, Lin Z, Gimelshein N, Antiga L, , 2019. Pytorch: An imperative style, high-performance deep learning library. Adv. Neural Inf. Process. Syst. 32, 8026–8037.

[R99] Pechenizkiy M, Tsymbal A, Puuronen S, Pechenizkiy O, 2006. Class noise and supervised learning in medical domains: The effect of feature extraction. In: 19th IEEE Symposium on Computer-Based Medical Systems. CBMS’06, IEEE, pp. 708–713.

[R100] Pedregosa F, Varoquaux G, Gramfort A, Michel V, Thirion B, Grisel O, Blondel M, Prettenhofer P, Weiss R, Dubourg V, Vanderplas J, Passos A, Cournapeau D, Brucher M, Perrot M, Duchesnay E, 2011. Scikit-learn: Machine learning in python. J. Mach. Learn. Res. 12, 2825–2830.

[R101] Persson K, Bohbot V, Bogdanovic N, Selbæk G, Brækhus A, Engedal K, 2018. Finding of increased caudate nucleus in patients with alzheimer’s disease. Acta Neurol. Scand. 137 (2), 224–232. 28741672 10.1111/ane.12800

[R102] Philippi N, Noblet V, Hamdaoui M, Soulier D, Botzung A, Ehrhard E, Cretin B, Blanc F, 2020. The insula, a grey matter of tastes: a volumetric MRI study in dementia with lewy bodies. Alzheimer’s Res. Therapy 12 (1), 1–11. 10.1186/s13195-020-00645-y PMC7336457 32631425

[R103] Plis SM, Hjelm DR, Salakhutdinov R, Allen EA, Bockholt HJ, Long JD, Johnson HJ, Paulsen JS, Turner JA, Calhoun VD, 2014. Deep learning for neuroimaging: a validation study. Front. Neurosci. 8, 229. 25191215 10.3389/fnins.2014.00229 PMC4138493

[R104] Plis SM, Weisend MP, Damaraju E, Eichele T, Mayer A, Clark VP, Lane T, Calhoun VD, 2011. Effective connectivity analysis of fMRI and MEG data collected under identical paradigms. Comput. Biol. Med. 41 (12), 1156–1165. 21592468 10.1016/j.compbiomed.2011.04.011 PMC3174276

[R105] Prawiroharjo P, Yamashita K. i., Yamashita K, Togao O, Hiwatashi A, Yamasaki R, Kira J. i., 2020. Disconnection of the right superior parietal lobule from the precuneus is associated with memory impairment in oldest-old alzheimer’s disease patients. Heliyon 6 (7). 10.1016/j.heliyon.2020.e04516 PMC7381702 32728647

[R106] Radford A, Kim JW, Hallacy C, Ramesh A, Goh G, Agarwal S, Sastry G, Askell A, Mishkin P, Clark J, Krueger G, Sutskever I, 2021. Learning transferable visual models from natural language supervision. In: ICML.

[R107] Radford A, Metz L, Chintala S, 2015. Unsupervised representation learning with deep convolutional generative adversarial networks. arXiv preprint arXiv:1511. 06434.

[R108] Raghu M, Gilmer J, Yosinski J, Sohl-Dickstein J, 2017. SVCCA: Singular vector canonical correlation analysis for deep learning dynamics and interpretability. In: Guyon I, Luxburg UV, Bengio S, Wallach H, Fergus R, Vishwanathan S, Garnett R (Eds.), Advances in Neural Information Processing Systems, Vol. 30. Curran Associates, Inc..

[R109] Rahaman MA, Garg Y, Iraj A, Fu Z, Chen J, Calhoun V, 2022. Two-dimensional attentive fusion for multi-modal learning of neuroimaging and genomics data. In: 2022 IEEE 32nd International Workshop on Machine Learning for Signal Processing. MLSP, IEEE, pp. 1–6.

[R110] Rahim F, Khalafi M, Davoodi M, Shirbandi K, 2023. Metabolite changes in the posterior cingulate cortex could be a signature for early detection of alzheimer’s disease: a systematic review and meta-analysis study based on 1H-NMR. Egypt. J. Neurol. Psychiatry Neurosurg. 59 (1), 60.

[R111] Ríos AS, Oxenford S, Neudorfer C, Butenko K, Li N, Rajamani N, Boutet A, Elias GJ, Germann J, Loh A, , 2022. Optimal deep brain stimulation sites and networks for stimulation of the fornix in alzheimer’s disease. Nat. Commun. 13 (1), 7707. 36517479 10.1038/s41467-022-34510-3 PMC9751139

[R112] Rokham H, Pearlson G, Abrol A, Falakshahi H, Plis S, Calhoun VD, 2020. Addressing inaccurate nosology in mental health: A multilabel data cleansing approach for detecting label noise from structural magnetic resonance imaging data in mood and psychosis disorders. Biol. Psychiatry Cogn. Neurosc. Neuroimag. 5 (8), 819–832. 10.1016/j.bpsc.2020.05.008 PMC7760893 32771180

[R113] Ronneberger O, Fischer P, Brox T, 2015. U-net: Convolutional networks for biomedical image segmentation. In: International Conference on Medical Image Computing and Computer-Assisted Intervention. Springer, pp. 234–241.

[R114] Ruan Y, Dubois Y, Maddison CJ, 2021. Optimal representations for covariate shift. arXiv preprint arXiv:2201.00057.

[R115] Ryu S-Y, Kwon MJ, Lee S-B, Yang DW, Kim T-W, Song I-U, Yang PS, Kim HJ, Lee AY, 2010. Measurement of precuneal and hippocampal volumes using magnetic resonance volumetry in Alzheimer’s disease. J. Clin. Neurol. 6 (4), 196–203. 21264200 10.3988/jcn.2010.6.4.196 PMC3024524

[R116] Saito K, Mukuta Y, Ushiku Y, Harada T, 2016. Demian: Deep modality invariant adversarial network. arXiv preprint arXiv:1612.07976.

[R117] Sendi MS, Zendehrouh E, Miller RL, Fu Z, Du Y, Liu J, Mormino EC, Salat DH, Calhoun VD, 2021. Alzheimer’s disease projection from normal to mild dementia reflected in functional network connectivity: a longitudinal study. Front. Neural Circ. 14, 87. 10.3389/fncir.2020.593263 PMC7859281 33551754

[R118] Sheng C, Xia M, Yu H, Huang Y, Lu Y, Liu F, He Y, Han Y, 2017. Abnormal global functional network connectivity and its relationship to medial temporal atrophy in patients with amnestic mild cognitive impairment. PLoS One 12 (6), e0179823. 28650994 10.1371/journal.pone.0179823 PMC5484500

[R119] Shi Y, Siddharth N, Paige B, Torr P, 2019. Variational mixture-of-experts autoen-coders for multi-modal deep generative models. In: Wallach H, Larochelle H, Beygelzimer A, d’Alché Buc F, Fox E, Garnett R (Eds.), Advances in Neural Information Processing Systems, Vol. 32. Curran Associates, Inc..

[R120] Sintini I, Schwarz CG, Martin PR, Graff-Radford J, Machulda MM, Senjem ML, Reid RI, Spychalla AJ, Drubach DA, Lowe VJ, , 2019. Regional multimodal relationships between tau, hypometabolism, atrophy, and fractional anisotropy in atypical alzheimer’s disease. Hum. Brain Mapp. 40 (5), 1618–1631. 30549156 10.1002/hbm.24473 PMC6615561

[R121] Smith L, Topin N, 2019. Super-convergence: Very fast training of neural networks using large learning rates. In: AI/ML MDO. SPIE.

[R122] Song X-W, Dong Z-Y, Long X-Y, Li S-F, Zuo X-N, Zhu C-Z, He Y, Yan C-G, Zang Y-F, 2011. REST: a toolkit for resting-state functional magnetic resonance imaging data processing. PLoS One 6 (9), e25031. 21949842 10.1371/journal.pone.0025031 PMC3176805

[R123] Srivastava N, Salakhutdinov R, 2012a. Learning representations for multimodal data with deep belief nets. In: International Conference on Machine Learning Workshop, Vol. 79. p. 3.

[R124] Srivastava N, Salakhutdinov RR, 2012b. Multimodal learning with deep boltzmann machines. Adv. Neural Inf. Process. Syst 25.

[R125] Stein JL, Hibar DP, Madsen SK, Khamis M, McMahon KL, de Zubicaray GI, Hansell NK, Montgomery GW, Martin NG, Wright MJ, , 2011. Discovery and replication of dopamine-related gene effects on caudate volume in young and elderly populations (N=1198) using genome-wide search. Molecul. Psychiatry 16 (9), 927–937. 10.1038/mp.2011.32 PMC3140560 21502949

[R126] Sui J, Adali T, Pearlson G, Yang H, Sponheim SR, White T, Calhoun VD, 2010. A CCA+ ICA based model for multi-task brain imaging data fusion and its application to schizophrenia. Neuroimage 51 (1), 123–134. 20114081 10.1016/j.neuroimage.2010.01.069 PMC2847043

[R127] Sui J, Pearlson G, Caprihan A, Adali T, Kiehl KA, Liu J, Yamamoto J, Calhoun VD, 2011. Discriminating schizophrenia and bipolar disorder by fusing fMRI and DTI in a multimodal CCA+ joint ICA model. Neuroimage 57 (3), 839–855. 21640835 10.1016/j.neuroimage.2011.05.055 PMC3129373

[R128] Suk H-I, Lee S-W, Shen D, 2014. Hierarchical feature representation and multi-modal fusion with deep learning for AD/MCI diagnosis. NeuroImage 101, 569–582. 10.1016/j.neuroimage.2014.06.077. 25042445 PMC4165842

[R129] Sundararajan M, Taly A, Yan Q, 2017. Axiomatic attribution for deep networks. In: International Conference on Machine Learning. PMLR, pp. 3319–3328.

[R130] Sylvain T, Dutil F, Berthier T, Di Jorio L, Luck M, Hjelm D, Bengio Y, 2020a. Cross-modal information maximization for medical imaging: Cmim. arXiv preprint arXiv:2010.10593.

[R131] Sylvain T, Petrini L, Hjelm D, 2019. Locality and compositionality in zero-shot learning. arXiv preprint arXiv:1912.12179.

[R132] Sylvain T, Petrini L, Hjelm D, 2020b. Zero-shot learning from scratch (ZFS): leveraging local compositional representations. arXiv:2010.13320.

[R133] Taleb A, Loetzsch W, Danz N, Severin J, Gaertner T, Bergner B, Lippert C, 2020. 3D self-supervised methods for medical imaging. In: Larochelle H, Ranzato M, Hadsell R, Balcan M, Lin H (Eds.), Advances in Neural Information Processing Systems, Vol. 33. Curran Associates, Inc., pp. 18158–18172, URL https://proceedings.neurips.cc/paper/2020/file/d2dc6368837861b42020ee72b0896182-Paper.pdf.

[R134] Tekin S, Mega MS, Masterman DM, Chow T, Garakian J, Vinters HV, Cummings JL, 2001. Orbitofrontal and anterior cingulate cortex neurofibrillary tangle burden is associated with agitation in alzheimer disease. Ann. Neurol. 49 (3), 355–361. 11261510

[R135] Tian Y, Krishnan D, Isola P, 2019. Contrastive multiview coding. arXiv preprint arXiv:1906.05849.

[R136] Tosun D, Joshi S, Weiner MW, Alzheimer’s Disease Neuroimaging Initiative, 2014. Multimodal MRI-based imputation of the A*β*+ in early mild cognitive impairment. Ann. Clin. Transl. Neurol. 1 (3), 160–170. 24729983 10.1002/acn3.40 PMC3981105

[R137] Tschannen M, Djolonga J, Rubenstein PK, Gelly S, Lucic M, 2020. On mutual information maximization for representation learning. In: International Conference on Learning Representations.

[R138] Virtanen P, Gommers R, Oliphant TE, Haberland M, Reddy T, Cournapeau D, Burovski E, Peterson P, Weckesser W, Bright J, van der Walt SJ, Brett M, Wilson J, Millman KJ, Mayorov N, Nelson ARJ, Jones E, Kern R, Larson E, Carey CJ, Polat İ, Feng Y, Moore EW, VanderPlas J, Laxalde D, Perktold J, Cimrman R, Henriksen I, Quintero EA, Harris CR, Archibald AM, Ribeiro AH, Pedregosa F, van Mulbregt P, SciPy 1.0 Contributors, 2020. SciPy 1.0: Fundamental Algorithms for Scientific Computing in Python. Nature Methods 17, 261–272. 10.1038/s41592-019-0686-2. 32015543 PMC7056644

[R139] Von Kügelgen J, Sharma Y, Gresele L, Brendel W, Schölkopf B, Besserve M, Locatello F, 2021. Self-supervised learning with data augmentations provably isolates content from style. Adv. Neural Inf. Process. Syst 34.

[R140] Walhovd KB, Fjell AM, Amlien I, Grambaite R, Stenset V, Bjørnerud A, Reinvang I, Gjerstad L, Cappelen T, Due-Tønnessen P, , 2009. Multimodal imaging in mild cognitive impairment: Metabolism, morphometry and diffusion of the temporal–parietal memory network. Neuroimage 45 (1), 215–223. 19056499 10.1016/j.neuroimage.2008.10.053

[R141] Walhovd K, Fjell A, Dale A, McEvoy L, Brewer J, Karow D, Salmon D, Fennema-Notestine C, Alzheimer’s Disease Neuroimaging Initiative, , 2010. Multi-modal imaging predicts memory performance in normal aging and cognitive decline. Neurobiology of aging 31 (7), 1107–1121. 18838195 10.1016/j.neurobiolaging.2008.08.013 PMC3947581

[R142] Wang W, Arora R, Livescu K, Bilmes J, 2015. On deep multi-view representation learning. In: International Conference on Machine Learning. PMLR, pp. 1083–1092.

[R143] Wang C, Shen Z, Huang P, Yu H, Qian W, Guan X, Gu Q, Yang Y, Zhang M, 2017. Altered spontaneous brain activity in chronic smokers revealed by fractional ramplitude of low-frequency fluctuation analysis: a preliminary study. Sci. Rep. 7 (1), 1–7. 28336919 10.1038/s41598-017-00463-7 PMC5428464

[R144] Weiler M, Agosta F, Canu E, Copetti M, Magnani G, Marcone A, Pagani E, Balthazar MLF, Comi G, Falini A, , 2015. Following the spreading of brain structural changes in alzheimer’s disease: a longitudinal, multimodal MRI study. J. Alzheimer’s Dis. 47 (4), 995–1007. 26401778 10.3233/JAD-150196

[R145] Wingo AP, Fan W, Duong DM, Gerasimov ES, Dammer EB, Liu Y, Harerimana NV, White B, Thambisetty M, Troncoso JC, , 2020. Shared proteomic effects of cerebral atherosclerosis and alzheimer’s disease on the human brain. Nature Neurosci. 23 (6), 696–700. 32424284 10.1038/s41593-020-0635-5 PMC7269838

[R146] Wu Z, Xiong Y, Yu SX, Lin D, 2018. Unsupervised feature learning via non-parametric instance discrimination. In: Proceedings of the IEEE Conference on Computer Vision and Pattern Recognition. pp. 3733–3742.

[R147] Wu B-S, Zhang Y-R, Li H-Q, Kuo K, Chen S-D, Dong Q, Liu Y, Yu J-T, 2021. Cortical structure and the risk for alzheimer’s disease: a bidirectional mendelian randomization study. Transl Psychiatry 11 (1), 476. 34526483 10.1038/s41398-021-01599-x PMC8443658

[R148] Xie C, Bai F, Yuan B, Yu H, Shi Y, Yuan Y, Wu D, Zhang Z-S, Zhang Z-J, 2015. Joint effects of gray matter atrophy and altered functional connectivity on cognitive deficits in amnestic mild cognitive impairment patients. Psychol. Med. 45 (9), 1799–1810. 25511078 10.1017/S0033291714002876

[R149] Xie T, Zhang X, Wang L, Xie H, Zhang J, Xu B, Wang H, Fu L, 2020. Relationships between tau, atrophy, regional brain activity and connectivity in Alzheimer’s disease: a PET/MRI multimodal study. Soc Nuclear Med.

[R150] Yang AC, Vest RT, Kern F, Lee DP, Agam M, Maat CA, Losada PM, Chen MB, Schaum N, Khoury N, , 2022. A human brain vascular atlas reveals diverse mediators of alzheimer’s risk. Nature 603 (7903), 885–892. 35165441 10.1038/s41586-021-04369-3 PMC9635042

[R151] Ye C, Mori S, Chan P, Ma T, 2019. Connectome-wide network analysis of white matter connectivity in alzheimer’s disease. NeuroImage: Clin. 22, 101690. 30825712 10.1016/j.nicl.2019.101690 PMC6396432

[R152] Zhang M, Huang X, Li B, Shang H, Yang J, 2022. Gray matter structural and functional alterations in idiopathic blepharospasm: A multimodal meta-analysis of VBM and functional neuroimaging studies. Front. Neurol. 13, 889714. 35734475 10.3389/fneur.2022.889714 PMC9207395

[R153] Zhang R, Isola P, Efros AA, 2016. Colorful image colorization. In: European Conference on Computer Vision. Springer, pp. 649–666.

[R154] Zhang L, Zuo X-N, Ng KK, Chong JSX, Shim HY, Ong MQW, Loke YM, Choo BL, Chong EJY, Wong ZX, , 2020. Distinct BOLD variability changes in the default mode and salience networks in Alzheimer’s disease spectrum and associations with cognitive decline. Sci. Rep. 10 (1), 6457. 32296093 10.1038/s41598-020-63540-4 PMC7160203

[R155] Zhou Y, Yu F, Duong TQ, Alzheimer’s Disease Neuroimaging Initiative, 2015. White matter lesion load is associated with resting state functional MRI activity and amyloid PET but not FDG in mild cognitive impairment and early alzheimer’s disease patients. J. Magn. Reson. Imaging 41 (1), 102–109. 24382798 10.1002/jmri.24550 PMC4097981

[R156] Zhu K, Wang X, Sun B, Wu J, Lu H, Zhang X, Liang H, Zhang D, Liu C, 2019. Primary age-related tauopathy in human subcortical nuclei. Front. Neurosci. 13, 529. 31191227 10.3389/fnins.2019.00529 PMC6549797

[R157] Zimny A, Szewczyk P, Trypka E, Wojtynska R, Noga L, Leszek J, Sasiadek M, 2011. Multimodal imaging in diagnosis of alzheimer’s disease and amnestic mild cognitive impairment: value of magnetic resonance spectroscopy, perfusion, and diffusion tensor imaging of the posterior cingulate region. J. Alzheimer’s Dis. 27 (3), 591–601. 21841260 10.3233/JAD-2011-110254

[R158] Zou Q-H, Zhu C-Z, Yang Y, Zuo X-N, Long X-Y, Cao Q-J, Wang Y-F, Zang Y-F, 2008. An improved approach to detection of amplitude of low-frequency fluctuation (ALFF) for resting-state fMRI: fractional ALFF. J. Neurosci. Methods 172 (1), 137–141. 18501969 10.1016/j.jneumeth.2008.04.012 PMC3902859

